# An effective approach for annotation of protein families with low sequence similarity and conserved motifs: identifying GDSL hydrolases across the plant kingdom

**DOI:** 10.1186/s12859-016-0919-7

**Published:** 2016-02-18

**Authors:** Ivan Vujaklija, Ana Bielen, Tina Paradžik, Siniša Biđin, Pavle Goldstein, Dušica Vujaklija

**Affiliations:** Faculty of Electrical Engineering and Computing, Unska 3, Zagreb, 10000 Croatia; Department of Biochemical Engineering, Faculty of Food Technology and Biotechnology, University of Zagreb, Pierrotijeva 6, Zagreb, 10000 Croatia; Division of Molecular Biology, Ruđer Bošković Institute, Bijenička 54, Zagreb, 10000 Croatia; Department of Mathematics, Faculty of Science, University of Zagreb, Bijenička 30, Zagreb, 10000 Croatia

**Keywords:** motif-HMM, Annotation errors, Low sequence similarity, GDSL family, Plant kingdom

## Abstract

**Background:**

The massive accumulation of protein sequences arising from the rapid development of high-throughput sequencing, coupled with automatic annotation, results in high levels of incorrect annotations. In this study, we describe an approach to decrease annotation errors of protein families characterized by low overall sequence similarity. The GDSL lipolytic family comprises proteins with multifunctional properties and high potential for pharmaceutical and industrial applications. The number of proteins assigned to this family has increased rapidly over the last few years. In particular, the natural abundance of GDSL enzymes reported recently in plants indicates that they could be a good source of novel GDSL enzymes. We noticed that a significant proportion of annotated sequences lack specific GDSL motif(s) or catalytic residue(s). Here, we applied motif-based sequence analyses to identify enzymes possessing conserved GDSL motifs in selected proteomes across the plant kingdom.

**Results:**

Motif-based HMM scanning (Viterbi decoding-VD and posterior decoding-PD) and the here described PD/VD protocol were successfully applied on 12 selected plant proteomes to identify sequences with GDSL motifs. A significant number of identified GDSL sequences were novel. Moreover, our scanning approach successfully detected protein sequences lacking at least one of the essential motifs (171/820) annotated by Pfam profile search (PfamA) as GDSL. Based on these analyses we provide a curated list of GDSL enzymes from the selected plants. CLANS clustering and phylogenetic analysis helped us to gain a better insight into the evolutionary relationship of all identified GDSL sequences. Three novel GDSL subfamilies as well as unreported variations in GDSL motifs were discovered in this study. In addition, analyses of selected proteomes showed a remarkable expansion of GDSL enzymes in the lycophyte, *Selaginella moellendorffii*. Finally, we provide a general motif-HMM scanner which is easily accessible through the graphical user interface (http://compbio.math.hr/).

**Conclusions:**

Our results show that scanning with a carefully parameterized motif-HMM is an effective approach for annotation of protein families with low sequence similarity and conserved motifs. The results of this study expand current knowledge and provide new insights into the evolution of the large GDSL-lipase family in land plants.

**Electronic supplementary material:**

The online version of this article (doi:10.1186/s12859-016-0919-7) contains supplementary material, which is available to authorized users.

## Background

The rapid development of enzyme technology has enabled the wide application of hydrolytic enzymes in industry and environmental management [1, 2]. The search for novel enzymes with beneficial functions has great potential in the GDSL lipolytic family. These enzymes have five consensus sequence Blocks (I-V), among which Blocks I, III and V show higher conservation. In addition, they possess four invariant catalytic residues, Ser, Gly, Asn, and His, found in Blocks I, II, III and V, respectively [3, 4]. A distinctive feature of the GDSL esterases/lipases is a flexible catalytic site that changes its conformation in the presence of different substrates [5–8]. This might explain their catalytic multifunctionality, which makes them an attractive research topic. GDSL hydrolases are found throughout all kingdoms of life and recent analyses revealed their abundance in some land plants [9, 10]. Although they participate in many cellular processes, such as plant development [11, 12], morphogenesis [13], and defense from pathogens and stress [14, 15], they are still poorly studied. The reported multifunctionality of GDSL enzymes indicates that plants could be a good source of highly promising enzymes for use in the hydrolysis and synthesis of important ester compounds of biotechnological interest [5, 16]. Therefore searching for new GDSL enzymes across the plant kingdom is of general interest. Since these enzymes exhibit low overall sequence similarity [5], motif scanning [17] is an appropriate method for *in silico* annotation of GDSL proteins. Considering that protein motifs accumulate mutations over time the task of motif scanning differs substantially from the simple problem of exact string matching and requires application of more sophisticated methods. The standard method for motif scanning is scanning by Position Specific Scoring Matrix (PSSM). This method is simply a “window sliding algorithm” where a “PSSM window” slides along the target sequence and scores each residue position according to the corresponding PSSM column scores. These scores represents the relative frequency of residues in one position in the given motif alignment [18, 19]. Another possible approach is motif-HMM scanning, which is rarely used. As the name suggests, it is based on the HMM probabilistic framework which has been widely and successfully applied in many areas of bioinformatics, such as protein structure modelling, gene finding, phylogenetic analysis, modelling coding and noncoding regions of DNA, and protein family and subfamily modelling [20]. Unlike the standard profile-HMM, which models a set of sequences (e.g. a protein family), motif-HMM models only motif(s), while protein regions between the motifs are modelled by a single self-looping insert state (Additional file 1). Thus the compositions of regions between the motifs are not important for the decoding algorithm [17]. The major difference between PSSM and motif-HMM, is that the latter explicitly models insertions and deletions as well as distances between different motifs (if multiple motifs are present) in a natural and straightforward way. A potential drawback of this is the introduction of additional parameters, which increases model complexity. This probably explains why motif-HMMs are so rarely used. However, in the multiple motif scanning setting, and in particular when additional parameters can be estimated based either on training examples (i.e. seed dataset) or on expert knowledge, motif-HMM should be a model of choice. The standard decoding algorithm within the HMM framework is Viterbi decoding (VD) which finds the most probable path through the model (i.e. HMM) assuming that the given HMM has generated the analyzed sequence. Another possible approach within the HMM framework is posterior decoding (PD) which maximizes the posterior probability of assigning an HMM state to the given residue, over all possible states. Although VD and PD will in most cases yield similar results, when different paths through the HMM have a similar probability as the most probable path, PD and VD might give different results. Moreover, in such cases PD might be preferable to Viterbi decoding since in posterior decoding all paths that contribute to a given assignment are taken into account [21–23].

In this study we introduce a simple modification of the PD algorithm which enables its application to the motif-HMM framework. Next, we applied and compared the performance of VD, PD and PSSM in identifying GDSL enzymes in proteomes selected from across the plant kingdom. Our results show that motif-HMM in the multiple motif scanning setting can outperform motif scanning algorithms based on matrix models. Since we started from a very small sample of experimentally confirmed enzymes, this required a careful computation of model parameters. Here we show, as an example, how well adjusted motif-HMM parameterization, in particular emission probabilities, can provide a good discrimination between positives and negatives, taking a Viterbi score of zero as a natural threshold. Based on this, we developed a PD/VD protocol which was useful for the elimination of false positives. Our protocol could be successfully applied for the automatic annotation of GDSL enzymes in a large dataset (e.g. metagenomic data sets). In summary, we scanned 12 proteomes across the plant kingdom for GDSL enzymes and obtained GDSL sequences distributed either in the previously reported [9] or in the three distinct and here discovered GDSL subfamilies. We also provide a list of so far undescribed variations in the GDSL motifs. Finally, a remarkable expansion of this protein family was found in a lycophyte, indicating an important role for GDSL enzymes in the early evolution of vascular plants.

## Methods

### Datasets collection

Protein sequences of euphyllophytes: *Arabidopsis thaliana -****At***; *Oryza sativa* - ***Os***; *Populus trichocarpa -****Pt***; *Sorghum bicolor -****Sb*** and *Vitis vinifera –****Vv***; moss, *Physcomitrella patens –****Pp***; algae: *Chlamydomonas reinhardtii -****Cr***; *Coccomyxa subellipsoidea* C-169 - ***Cs***; *Micromonas pusilla* RCC299 - ***Mp***; *Ostreococcus lucimarinus -****Ol***; *Volvox carteri -****Vc****,* and bryophyte, *Selaginella moellendorffi -****Sm***, were retrieved from NCBI RefSeq Genome Projects. Proteomes of algae and bryophyte were retrieved from Phytozome v9.1 [24].

### Annotation criteria

Motifs characteristic for GDSL enzymes and positions of catalytic residues were reported previously [4, 5]. In accordance with that, our criteria for GDSL annotation were: (**i**) presence and correct order of specific motifs (Blocks I, III and V) (**ii**) presence of catalytically important Ser (Block I) and His (Block V) (Additional file 2: Figure S2), and (**iii**) minimal distances between predicted blocks of at least 20 residues.

### Seed dataset

Seed dataset used for model parameterization consisted of 23 protein sequences of biochemically characterized and evolutionary divergent GDSL enzymes (Additional file 2: Table S1) that shared on average 11 % identity, ranging from 4 to 75 %. Multiple sequence alignment (MSA) generated by PROMALS [25] and MAFFT [26] predicted the same motifs. MSA displayed three conserved motifs and long gaps between them. (Additional file 2: Figure S1). We selected Blocks I, III and V consisting of 10, 9, 9 residues, respectively (Additional file 2: Figure S2). The corresponding motif columns were used to parameterize HMM and PSSM.

### Emission probabilities

Probability of emitting a residue from the HMM-match state was computed as follows. Let **P** be a mutation probability matrix, constructed from the BLOSUM 50 substitution matrix [27, 28]. We denote the *l-th* row of this matrix with **P**_*l*_ = [*p*_*ls*_], where *p*_*ls*_ is the probability of amino acid *l* mutating into amino acid *s* after a certain amount of evolutionary time. Given a motif of length *n*, let **F**_*R*_^*j*^ = [*f*_*l*_^*j*^] be a vector of amino acid relative frequencies in the *j* − *th* column of the motif alignment, where *j* ∈ {1 … … *n*} and *l* ∈ {1, … … 20}.

Considering a very small size of the seed dataset, we added a small amount of flat pseudo-counts – 10^-2^ for each amino acid *l* – to each relative frequency vector, getting vectors ${\widehat{\mathbf{F}}}_R^j=\left[{\widehat{f}}_l^j\right]$ where

$$ {\widehat{f}}_l^j=\frac{f_l^j+0.01}{1.2} $$

We then define emission probability of amino acid *k* in the *j* − *th* column as

$$ {e}^j(k)={\displaystyle \sum_{l=1}^{20}}{p}_{lk} \cdot {\widehat{f}}_l^j\kern1.75em l,k\in \left\{1,\dots \dots 20\right\} $$

Since *j* − *th* column of the motif alignment is modelled by the HMM match state M_j_ this is also the emission probability of amino acid *k* in the match state *j*, *e*_*j*_(*k*). Note that we made a slight change of notation here in order to distinguish between the PSSM and HMM emission probabilities. Emission probabilities constructed in this way combine the vector of relative frequencies with mutation probability vectors, as well as background distribution. Emission probabilities defined in this way are “loose” in the sense that even residues that are completely conserved in the seed sequences are assigned relatively low emission probabilities (e. g. completely conserved Ser in Block I and His in Block V were assigned emission probabilities of 0.64 and 0.93 respectively. While using a model of evolution of amino acids is a standard practice when computing emission probabilities, the details of our implementation are specific.

Insert states were assigned a flat emission probability vector, i.e. ${e}_{I_j}(k)=0.05$ where *j* ∈ {1 … … *n*} and *k* ∈ {1, … … 20}.

Same emission probabilities for all amino acids were used for all three algorithms.

### Transition probabilities

Since the seed alignment contains no insertions or deletions, transition probabilities to and from delete and insert states could not be estimated from the sample and needed to be inferred. These values have to be small (i.e. match-to-match transitions should clearly dominate) because insertions and deletions in GDSL motifs are not expected. On the other hand, although unlikely, we wanted to accommodate for the possibility of insertion or deletion within the GDSL blocks. Therefore, we tested transition probabilities of 0.90, 0.95, and 0.99 and used transition probabilities of 0.99 between neighboring match states as the best choice. Transition probabilities to and from delete/insert states were assigned accordingly to 0.005:

**Table:** 

${t}_{M_i{M}_{i+1}} = 0.99$	${t}_{M_i{I}_i}=0.005$	${t}_{M_i{D}_{i+1}}=0.005$
${t}_{I_i{M}_{i+1}}\kern0.5em =0.99$	${t}_{I_i{I}_i}\kern0.5em =0.01$	${t}_{I_i{D}_{i+1}}\kern0.5em =0$
${t}_{D_i{M}_{i+1}} = 0.99$	${t}_{D_i{I}_i} = 0$	${t}_{D_i{D}_{i+1}} = 0.01$

Here M, I and D denote the match, insert and delete HMM states, respectively. For example, ${t}_{M_i{M}_{i+1}}$ denotes the probability of transition from match state M_i_ to match state M_i + 1_. Schematic representation of the model is shown in Additional file 1. Same transition probabilities were used for VD and PD.

### Scanning by Position-specific scoring matrix (PSSM)

Algorithm inputs are target sequence and PSSM which represents motif alignment. Namely, given a motif alignment of width *n*, PSSM is a 20 × *n* matrix, where each matrix column *j ∈* {1,.. *n*} is a vector of logarithms of emission probabilities *ln*[*e*^*j*^(*k*)] where *k* ∈ {1, … … 20} is an amino-acid index.

The PSSM score of the predicted motif starting at position *i* in the target sequence is defined as the sum of scores of amino acids at sequence positions *i* to *i* + *n* − 1, matched with the corresponding 1 to *n* columns of the PSSM. Therefore in the target sequence of length *m*, there are *m* − *n* + 1 possible motif starting positions (i.e. possible motif predictions). For a given sequence, PSSM scanning algorithm returns a motif prediction, in terms of residue positions, with the highest PSSM score [18].

Since we aimed to identify sequences containing three GDSL motifs (Blocks I, III and V), each sequence was scanned with three different PSSMs. The scans were performed independently, meaning that each PSSM returned the best motif prediction irrespective of the other two. PSSM column vector emissions were set to logarithms of emission probabilities of the corresponding HMM match state. As in the case of PD, we defined the total score as the sum of the three motif scores. All scanned sequences were subsequently ranked according to their respective sequence score.

### Viterbi decoding (VD)

Although Viterbi decoding is standard and widely used HMM algorithm we describe it here in detail, for the sake of clarity and completeness.

Given the observed sequence **X**, and the state path **Π**, the most probable path through the model is defined by, ${\boldsymbol{\Pi}}^{\mathrm{V}}= \arg \underset{\ \boldsymbol{\Pi}}{ \max }P\left(\mathbf{X},\ \boldsymbol{\Pi}\ \Big|\ M\right)$

Let *v*_*k*_(*i*) denote the probability of the most probable path **Π** which ends in state *k* after emitting *x*_1_ … … *x*_*i*_. Given the following recursions [21],

$$ {v}_k(i)=\left\{\begin{array}{c}\hfill 1\kern0.5em \mathrm{f}\mathrm{o}\mathrm{r}\kern0.75em i=0\kern0.75em \mathrm{and}\kern0.5em k=\mathrm{BEGIN}\kern0.75em \hfill \\ {}\hfill 0\kern0.5em \mathrm{f}\mathrm{o}\mathrm{r}\kern0.75em i=0\kern0.75em \mathrm{and}\kern0.5em k\ne \mathrm{BEGIN}\kern0.75em \hfill \\ {}\hfill {e}_k\left({x}_i\right)\cdot \underset{k-1}{ \max }\ \left\{{v}_{k-1}\left(i-1\right)\cdot {t}_{k-1,\ k}\right\}\hfill \\ {}\hfill {\mathrm{pointer}}_i(k)=\underset{k-1}{\mathrm{argmax}}\ \left\{\ {v}_{k-1}\left(i-1\right)\cdot {t}_{k-1,k}\ \right\}\hfill \end{array}\right. $$

probability of the most probable path though the model is [21]

$$ P\left(x,{\pi}^V\right)=\underset{k}{ \max }\ \left\{{v}_k(L)\cdot {t}_{k,\  END}\right\} $$

The path itself can be found by backtracking [21]

$$ \begin{array}{c}\hfill \kern1em \hfill \\ {}\hfill\ \hfill \\ {}\hfill {\pi}_L^V={\mathrm{argmax}}_k\left\{\ {v}_k(L)\cdot {t}_{k, END\ }\right\}\hfill \\ {}\hfill\ \hfill \\ {}\hfill \kern2.5em {\pi}_{i-1}^V={\mathrm{pointer}}_i\left({\pi}_i^V\right)\kern1.25em for\ i=L,\dots ..1\hfill \end{array} $$

Note that Viterbi decoding amounts to registering symbols generated by Viterbi path. These are residues or - possibly - gaps. As already mentioned, in this work we have used a motif-HMM instead of the standardly used profile-HMM (Additional file 1). Viterbi assignments were scored with the (log -) probability of their path, normalized for the length (hence, log – odds score).

### Posterior decoding (PD) for motif-HMM

In this subsection we describe the standard posterior decoding and the introduced modification. Given a sequence and HMM model, probability that the sequence was generated by this particular HMM can be found either by forward or by backward recursion. More formally, let **X** = (*x*_1_, … … *x*_*L*_) be a sequence of amino acid symbols (i.e. residues) of length *L* and **Π** = (*π*_1_ …. *π*_*L*_) a sequence of HMM emitting states (i.e. match and insert states) that generate **X**. Hence, *π*_*i*_ represents the HMM state which generates *x*_*i*_. Note that **Π** is not necessarily a path through the HMM since it doesn’t contain delete states. However, any path (i.e. valid path) has a unique representation in terms of **Π** and vice-versa each **Π** defines a unique HMM path. Therefore we use **Π** as a symbol for the respective HMM path as well. Forward and backward probabilities [21] are defined as:

$$ \begin{array}{l}{f}_k(i)=P\left({x}_{1,}\dots .{x}_i,\kern0.5em {\pi}_i=k\right),\\ {}{b}_k(i)=P\left({x}_{i+1}{x}_L\ \Big|\ {\pi}_i=k\right),\end{array} $$

where *k* denotes an emitting HMM state i.e. *k* ∈ {M_k_, I_k_}. Forward and backward probabilities can be found by the following recursions [21]:

$$ \begin{array}{l}{f}_{k+1}(i) = \left\{\begin{array}{c}\hfill \kern1.5em 1\kern0.5em \mathrm{f}\mathrm{o}\mathrm{r}\kern0.75em i=0\kern0.75em \mathrm{and}\kern0.5em k+1=\mathrm{BEGIN}\kern0.75em \hfill \\ {}\hfill \kern1em 0\kern0.5em \mathrm{f}\mathrm{o}\mathrm{r}\ i=0\kern0.5em \mathrm{and}\kern0.5em k+1\ne \mathrm{BEGIN}\hfill \\ {}\hfill \kern0.5em \hfill \\ {}\hfill \kern1em {e}_{k+1}\left({x}_i\right){\displaystyle \sum_k}{f}_k\left(i-1\right)\cdot {t}_{k,k+1}\kern0.5em \mathrm{o}\mathrm{therwise},\kern0.5em i=\left(1\dots \mathrm{L}\right)\ \hfill \end{array}\right.\\ {}{b}_k(i)=\left\{\begin{array}{c}\hfill {b}_k(L)={t}_{k, END}\kern1.25em i=L,\kern0.75em \mathrm{f}\mathrm{o}\mathrm{r}\ \mathrm{all}\ \mathrm{states}\kern0.75em k\hfill \\ {}\hfill \kern1em {\displaystyle \sum_{k+1}}{t}_{k,k+1}\cdot {e}_{k+1}\left({x}_{i+1}\right)\cdot {b}_{k+1}\left(i+1\right)\kern1em i=\left(L-1\dots .1\right)\hfill \end{array}\right.\ \end{array} $$

where *e*_*k*_(*x*_*i*_) is probability of emitting residue *x*_*i*_ when in state *k*, and *t*_*k*,*k* + 1_ is probability of transition from state *k* to state *k* + 1. Probability of observing the sequence **X,** given the model *M* is defined by,

$$ P\left(\mathbf{X}\Big|M\right)={\displaystyle \sum_{\boldsymbol{\Pi}}}P\left(\mathbf{X},\boldsymbol{\Pi}\ \Big|M\right) $$

and can be found either by forward or backward recursion [21]:

$$ \begin{array}{l}P\left(\mathbf{X}\Big|M\right) = {\displaystyle \sum_k}{f}_k(L)\cdot {t}_{k, END}\\ {}P\left(\mathbf{X}\Big|M\right) = {\displaystyle \sum_k}{b}_k(1)\cdot {e}_k\left({x}_1\right)\cdot {t}_{BEGIN,k}\end{array} $$

Conditional probability that symbol *x*_i_ was emitted from the state *k*, given sequence **X** is defined by

$$ P\left({\pi}_i=k\Big|\mathbf{X},M\right)=\frac{{\displaystyle {\sum}_{\left(\boldsymbol{\Pi} \Big|{\pi}_i=k\right)}}P\left(\mathbf{X},\boldsymbol{\Pi} \Big|M\right)\ }{{\displaystyle {\sum}_{\boldsymbol{\Pi}}}P\left(\mathbf{X},\boldsymbol{\Pi} \Big|M\right)}\kern1.5em k\in\ \left\{{\mathrm{M}}_{\mathrm{k}},{\mathrm{I}}_{\mathrm{k}}\right\} $$

Thus, *P*(*π*_*i*_ = *k*|**X**,*M*) is the sum of probabilities of all paths through the model which emit *x*_*i*_ from the state *k*, divided by the sum of probabilities of all paths through the model (regardless of whether they emit *x*_*i*_ in the state *k* or not). Furthermore, the posterior probability can easily be found by [21]

$$ P\left({\pi}_i=k\Big|\mathbf{X},M\right)=\frac{f_k(i)\cdot {b}_k(i)}{P\left(\mathbf{X}\Big|M\right)} $$

Conventional posterior decoding maximizes posterior probability of assigning the HMM state *k* to the residue *x*_*i*_ over all states *k*. However, in the case of motif-HMM, match states representing the motif are known. Thus, in this case it is more meaningful to find the assignment of residues to the given state *M*_*k*_. Therefore, for a given match state *M*_*k*_, we maximized posterior probability over all possible emissions *x*_*i*_. Namely, let *s*_*k*_ be the maximum posterior probability for the match state *M*_*k*_ over all possible emissions *x*_*i*_, i.e.

$$ {s}_k=\underset{i}{ \max }P\left({\pi}_i={M}_k\Big|\mathbf{X},M\right)\kern2em i\in \left\{1\dots \dots L\right\} $$

Let the residue that maximizes posterior probability at *M*_*k*_ be *x*_*j*(*k*)_ where

$$ j(k) = \underset{i}{\mathrm{argmax}}P\left({\pi}_i={M}_k\Big|\mathbf{X},\kern-.2em M\right) $$

After finding *s*_*k*_ and *x*_*j*(*k*)_ for all *M*_*k*_ in the motif, we defined the score of the motif as

$$ S={s}_1\cdot \dots \cdot {s}_n $$

where *n* is the length of the motif. Such scoring where posterior probabilities are multiplied is intuitively appealing, because one is looking for a joint probability of events (namely, that *x*_*i*_ was emitted by *M*_*k*_ AND *x*_*i* + 1_ by *M*_*k* + 1_ AND etc.). Taking the product of posterior probabilities is an approximation of the probability of the joint event since *s*_*k*_ do not correspond to independent events. However, when posterior probabilities are large enough, taking the product becomes a good approximation of the total probability (i.e. total motif score). On the other hand, low values of posterior probabilities will correspond to assignments that will be discarded in any case.

If there is more than one motif present one can score the sequence either by summing or by multiplying individual motif scores. We have observed that summing individual motif scores produced slightly better ranking of GDSL sequences in the test-proteome (*A. thaliana*). This is due to the fact that summing individual motif scores compensates for lower scoring motifs. Namely, if the target sequence is composed of one low and two high scoring motifs, this sequence might be a positive with unusual mutations in one motif. Summing the scores of individual motifs instead of multiplying them penalizes such sequences less. Therefore, we defined the total score *S*_*T*_ as,

$$ {S}_T={\displaystyle \sum_{m=1}^3}{S}^m={\displaystyle \sum_{m=1}^3}\left({s}_1^m\cdot \dots \cdot {s}_{n(m)}^m\right) $$

where *m* ∈ {1, 2, 3} is index of the motif and *n*(*m*) is the corresponding length of the motif *m* and *s*_*k*_^*m*^ is the maximal posterior probability at state *M*_*k*_ in the motif *m*. In our case these were *n*(1) = 10, *n*(2) = 9, *n*(3) = 9 for Blocks I, III and V respectively (Additional file 1 and Additional file 2: Figure S2). Algorithm ranks sequences by the highest total score *S*_*T*_, and returns posterior probabilities *s*_*k*_^*m*^ along with the corresponding residue positions *j*^*m*^(*k*).

### PD/VD protocol

PD/VD protocol was developed to decrease the false discovery proportion of GDSL sequences. This protocol discards all sequences which do not have “approximately the same” (see criteria below) predicted motif positions by PD and VD, for all three motifs.

**Fig. 1:**
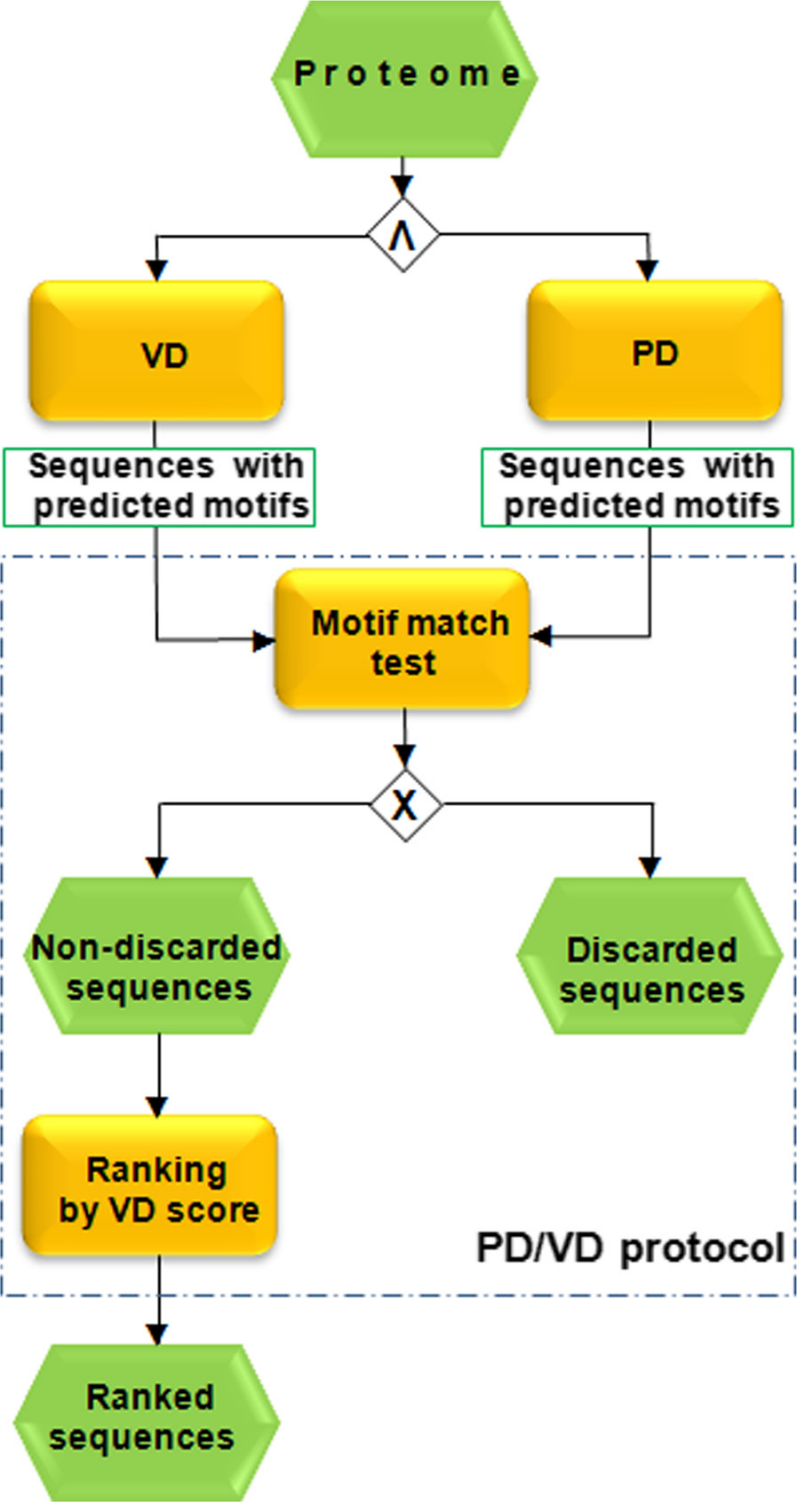
Flowchart of the PD/VD protocol

Unlike VD, PD does not necessarily return a sequence of consecutive motif-residue positions. To illustrate, motif-residue positions returned by PD, might be consecutive such as 40-41-42-43-44-45, “almost consecutive” as in 40-41-41-43-44-45, or separated by long intervals such as 40-41-42-70-71-72. If the motif-residue positions, returned by PD were consecutive or almost consecutive and were also the same as those returned by VD, for all three motifs, this was considered as match (PD/VD match). Otherwise, sequence was discarded from the further analyses (Fig. 1). More precisely, if for the two corresponding motifs predicted by VD and PD criteria stated below were satisfied this was considered to be a PD/VD match:

(i) Absolute value of the difference of arithmetic means of motif-residue positions returned by PD and those returned by VD had to be < 3, for all three motifs.
  For example, if motif-residue positions returned by VD and PD were 40-41-42-43-44-45 and 40-41-42-70-71-72 respectively, this would not be considered as a match, since the absolute value of the difference between arithmetic means is > 3. Namely, the arithmetic mean of the first sequence is 42.5, the second 56 and |56-42.5| > 3.
(ii) Distance between two neighbouring motif residue positions predicted by PD is not greater than two. For example 44-45-47-48-49-50 would be allowed but 44-47-48-49-50-51 would not. This was introduced since deletions of more than two residues in the GDSL blocks are highly unlikely. Note that this condition also discards unlikely but theoretically possible cases where the difference between the two means is < 3 but is nevertheless biologically meaningless. For example, if PD returned 20-21-22-61-62-64 (arithmetic mean 42) and VD returned 40-41-42-43-44-45 (arithmetic mean 43) this would not be considered as a PD/VD match.
  All sequences which passed the filter were subsequently ranked by VD score.

### CLANS and phylogenetic analyses

Cluster Analysis of Sequences (CLANS) is Java software that runs BLAST on given sequences, all-against-all, and clusters them in 2D or 3D according to their similarity [29]. In this study CLANS was used to visualize similarities of GDSL sequences identified by our motif scan from 12 plant proteomes (533 protein sequences). CLANS analysis was performed using a P-value cut-off of 10^−15^.

Protein sequences for phylogenetic analysis were randomly chosen from GDSL subgroups identified by CLANS (Fig. 7): A (56), B (35), C (6), D (12), E (3) and F (3). In total 115 GDSL sequences are listed in Additional file 6. These sequences represented all taxa found in each group and their number was proportional to the group size. MSA of GDSL protein sequences was obtained using E-INS-i alignment strategy, as suggested by the MAFFT server documentation for cases < 200 sequences with multiple conserved domains and long gaps [26]. GBlocks [30] was used to select the conserved regions in MSA by applying the following parameters: (**i**) minimum number of sequences for a conserved position = 58; (**ii**) minimum number of sequences for a flank position = 58; (**iii**) maximum number of contiguous non conserved positions = 100; (**iv**) minimum length of a block = 4; (**v**) allowed gap positions = with half. Finally, 106 aligned columns were selected for further phylogenetic analysis. LG + I + G was selected as the optimal model of protein evolution using PROTTEST 2.4 [31]. Maximum likelihood (ML) phylogenetic tree was constructed by PhyML 3.0 [32]. Nodal supports were tested by approximate likelihood-ratio test, aLRT [33].

## Results and discussion

Over the past ten years we have been studying GDSL lipolytic enzymes of bacterial origin [34–37]. Here we aimed to scan for the presence of GDSL motifs in proteomes selected across the plant kingdom. While analyzing previously reported protein sequences annotated as GDSL enzymes we noticed that numerous sequences were lacking specific block(s) and/or catalytic residues known to be essential for GDSL enzyme activities. Therefore, we have developed and applied a motif scanning approach to identify all sequences possessing the most conserved GDSL Blocks (I, III and V). To examine the possible benefits of motif scanning for the identification of GDSL sequences, we firstly applied PSSM, VD and PD on the well-curated *A. thaliana* proteome [9]. Note that VD and PSSM are widely used, whereas PD is not a standard motif scanning method [21–23].

### Evaluation of different scanning methods on *A. thaliana* proteome

**Fig. 2:**
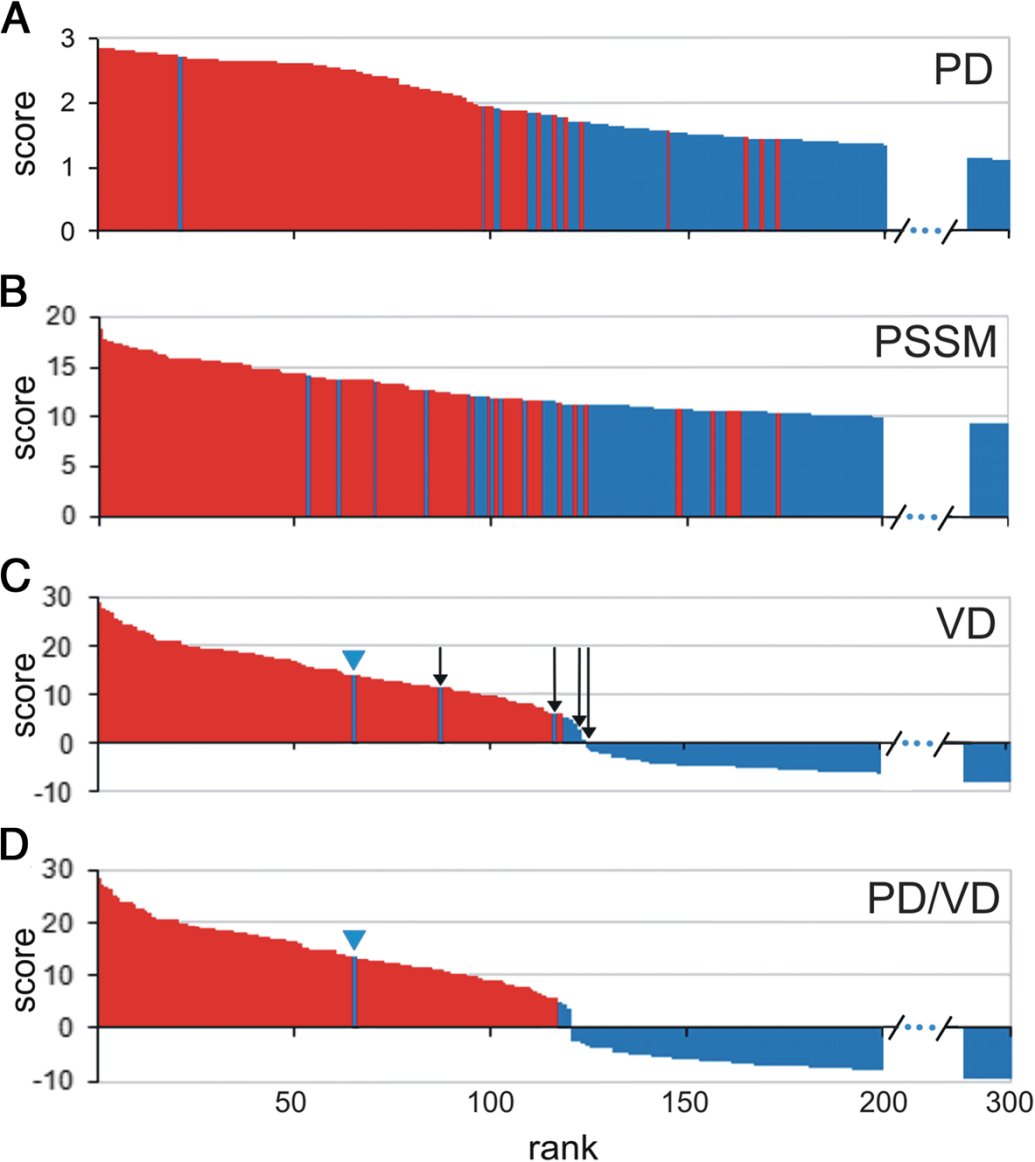
Top 300 *A. thaliana* protein sequences ranked by different algorithms. **a** Posterior decoding; **b** Position-specific scoring matrix; **c** Viterbi decoding; **d** PD/VD protocol. Positives (GDSL sequences) - red bars; negatives – blue bars. Negative sequences (not GDSL) with positive VD scores which were removed by PD/VD protocol are marked with the black arrows and sequence At1g54030, in detail described in the text is marked with the blue triangle

Since the *A. thaliana* proteome has been extensively analyzed, we used it to test the relative performance of PD, PSSM and VD. The results are shown in Fig. 2. Visual inspection confirmed that all three methods ranked GDSL enzymes (red bars - Fig. 2) at the top of 35,176 protein sequences present in the *A. thaliana* proteome (top ranked 300 sequences are shown in Fig. 2). VD displayed the best ranking, followed by PD and PSSM. Note that in the VD output list all sequences with GDSL motifs (116) obtained positive scores while sequences without the required GDSL blocks obtained scores lower than GDSL sequences, with three exceptions (blue bars - Fig. 2c). The reasons for the high ranking of these sequences lies in the fact that the sequence ranked as 66th [TAIR: At1g54030] possesses well conserved required motifs with only one but essential mutation in catalytic site in Block I (Ser/Gly) (Fig. 2c, At1g54030 is marked with the blue triangle). The other two sequences ranked as 88^th^ and 117^th^ [TAIR: At3g4355 and At4g16230 respectively] (Fig. 2c) although lacking Block V possess two highly conserved motifs, Block I and III that contributed significantly to their scores. Noteworthy, At1g54030 was shown to lack lipase activity [38–40]. Its retention in the genome should be ascribed to the fact that although preserving high overall similarity with the GDSL family this enzyme acquired a novel cellular function [41] and became a very good example of a pseudoenzyme. In any case one would not expect a high number of such sequences in the proteome. As shown in *A. thaliana* only 3 out of 116 GDSL sequences represent a group of dead enzymes. Therefore sequences with similar properties should not constrain the overall performance of our method.

Next, we compared our VD output list (116 *A. thaliana* sequences with GDSL motifs, Additional file 3, *A. thaliana* sheet, Table A) with Volokita et al [9]. By applying iterative PsiBlast this group recently reported only 100 GDSL sequences in *A. thaliana.* Out of these, 97 sequences were present in our list while the remaining three were omitted from our list since these sequences were lacking Block I or V (Additional file 3, *A. thaliana* sheet, Table B). This first test indicated that our motif scanning method could be a very good option for annotation of GDSL sequences in a given data set.

### Additional scanning for GDSL lipases in selected plant proteomes

**Fig. 3:**
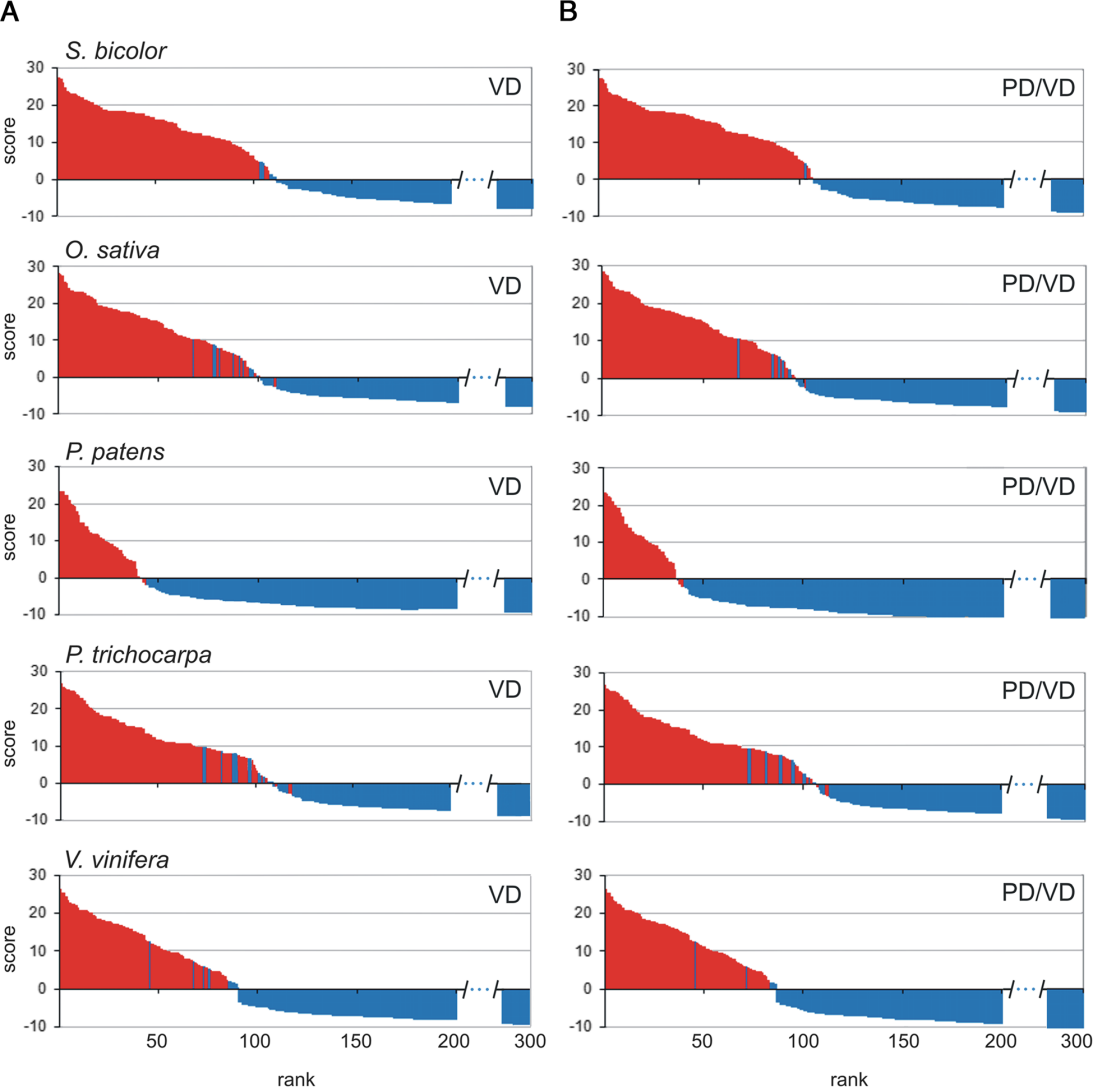
Top 300 sequences from the selected proteomes ranked by different algorithms. **a** Viterbi decoding; **b** PD/VD protocol. Positives (GDSL sequences) - red bars; negatives (not GDSL sequences) – blue bars

We scanned five additional land plant proteomes (*S. bicolor, O. sativa, P. patens, P. trichocarpa* and *V. vinifera*) with our GDSL motif–HMM. According to previous analysis, approximately 100 GDSL sequences should be expected in each analyzed proteome [9, 10]. We decided to inspect visually 300 of the best ranked sequences to classify all sequences with conserved GDSL blocks (positives) and without GDSL blocks (negatives). We used VD as a motif scanning method, since it showed the best results on the A*. thaliana* proteome. The results of these analyses are shown graphically in Fig. 3a. As in the case of *A. thaliana* the same trend of ranking GDSL sequences at the top of the output list was noticed. Namely, all identified GDSL sequences in all five analyzed proteomes were ranked at the top (within 0.2 %-0.6 %) of the proteome output list. Moreover, almost all GDSL sequences obtained a positive score (Fig. 3a) and therefore zero was used as a threshold score to separate positives from negatives in further analyses. Next, we compared our results with a previously reported GDSL list [9]. As can be seen, in each proteome we identified novel sequences (138 in total, Additional file 3, Tables A – rows labelled grey). It should be pointed out that during this analysis we noticed discrepancies in the data sets used in these two independent studies. Therefore we have built a novel and comprehensive list of GDSL sequences with all important details. Description of GDSL revised list is provided in the next paragraph.

Taken together, the presented results show that our parameterization although not standard was very effective. Namely, starting from a very small number of seed sequences (23) that were experimentally confirmed to possess lipolytic activities (Additional file 2: Table S1) and using a combination of flat and evolutionary pseudo counts, we have built a model that effectively scanned for GDSL motifs in six plant proteomes.

As in the case of *A. thaliana* we inspected all protein sequences in the VD output lists that, although lacking required GDSL motifs, obtained positive scores and *vice versa* all sequences that although possessing GDSL motifs obtained negative scores, i.e. false positives (FP) and false negatives (FN) respectively. In total, when taking zero as a threshold, 49 sequences from the six proteomes (208,298 sequences) were classified as FP and seven as FN. It should be emphasized that a zero-threshold is a very stringent criterion. Moreover, taking into account an average expected number of GDSL sequences (around 100 per proteome), the fact that only seven FN were identified shows a very high sensitivity (98.7 %) for this method. Note also that all FN obtained negative scores close to zero. Therefore, sequences around zero should be carefully examined. Analyses of FPs (49) showed that the majority of these proteins (46/49) possess GDSL motifs with mutated residue(s). Out of these 46 sequences, five were mutated in catalytic sites (3 carrying 1, and 2 carrying 2 mutations), whereas 41 were missing one of the Blocks (25 - Block V, 15 - Block I, 1 - Block III) but the other two were highly conserved thus contributing to the high score. These sequences most likely represent pseudoenzymes as described previously in *A. thaliana* and as such could be of interest for further studies. Note that only 3/49 FPs found in a total of 208,298 sequences did not display any global similarity to GDSL members, but the HMM-based motif scan found short stretches of amino acids which resemble GDSL motifs.

### Revised list of GDSL sequences

As stated above, the results of the motif scanning analyses of selected proteomes and their comparison with previously reported GDSL sequences [9] led us to create a revised list of GDSL sequences (Additional file 3, Tables A, B and C). We discriminate three categories:

(A) In this study identified GDSL sequences from the RefSeq proteomes. As depicted in Additional file 3, Table A, GDSL sequences not reported previously are shown in grey.
(B) Previously reported GDSL sequences not identified in our study but traced in RefSeq via provided gene loci [9] were analyzed and are listed in Additional file 3, Table B. As shown, all of these sequences were lacking one or two required motifs and were therefore omitted from our list. On the other hand, all sequences provided in [9] were also analyzed and divided into two subgroups: those which are GDSL or those lacking the required motif(s).
(C) Previously reported GDSL enzymes [9] that could not be traced since standard gene identifier was not provided were also categorized based on the presence or absence of GDSL motifs (Additional file 3, Table C).

As a general conclusion it should be pointed out that comparison with previously reported GDSL lists [9] was rather difficult in some cases since proteome databases have undergone many changes and many protein sequences have been revised. Inconsistencies in protein databases as well as differences between protein identifiers observed in this study are a well known problem in proteomics [42]. The rationale for using RefSeq as a data source for proteomes analyzed in this study was based on a recent publication [43], its widespread use and the fact that it is manually curated. The best match with previous results [9] was obtained for *A. thaliana*, thus indicating a carefully curated proteome (Additional file 3, sheet *A. thaliana*). On the contrary, for other analyzed proteomes we used gene loci to track all sequences which were not 100 % identical with those reported previously. For example, in the *S. bicolor* proteome we have identified 105 GDSL sequences; 102/105 matched previously reported sequences [9], whereas three were novel. An additional 28 sequences were found by Volokita et al [9] (Additional file 3, sheet *S. bicolor*, Tables B, C). However 10/28 sequences were not present in RefSeq and therefore could not be identified here while 18/28 of these reported sequences [9] were lacking at least one of the conserved motifs (Additional file 3, sheet *S. bicolor*, Table B) and therefore were not identified as GDSL enzymes in this study. A similar analysis was performed for *O. sativa* (Additional file 3, sheet *O. sativa*). For the additional three proteomes, *P. patens, V. vinifera* and *P. trichocarpa* comparison using protein identifiers was not possible since the majority of those provided in [9] could not be matched with the NCBI accession numbers. Altogether, in these three proteomes 101 GDSL sequences were not reported previously, whereas 120 sequences matched (100 % identity) the previous GDSL list (Additional file 3, sheets *P. patens, V. vinifera* and *P. trichocarpa*).

Regardless of the differences in proteome data sources our results demonstrate the benefit of the motif scan approach since in well defined data sets we identified novel sequences and found many previously reported GDSL sequences which lack GDSL conserved block(s) (Additional file 3, Tables B, C) and therefore should not be annotated as GDSL enzymes. All protein sequences possessing the required GDSL blocks, reported previously but not found here (since not present in RefSeq) obtained high scores in approximately the same range as GDSL sequences in Additional file 3, Tables A. This clearly shows that these sequences could have been identified if present in our data set.

**Fig. 4:**
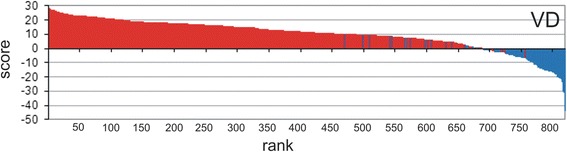
VD scores of selected plant proteomes from Pfam database - Lipase_GDSL (PF00657). Out of 820 sequences, 649 possessed all three conserved motifs (red bars) whereas 171 did not (blue bars)

The fact that each proteome contained some proportion of GDSL sequences displaying a family profile similarity but lacking specific motif(s) prompted us to perform an additional analysis. We used the GDSL sequences from the Pfam database since Pfam collects the sequences related by overall sequence similarity, i.e. sequence family profile [44]. We selected the Pfam database Lipase_GDSL (PF00657) family to retrieve all GDSL sequences from the species analyzed in this study. An additional two Pfam families were also checked (PF13472, Lipase_GDSL_2 and PF14606, Lipase_GDSL_3), but they did not contain any sequences from the here selected plant proteomes and therefore were not analyzed. In total 820 protein sequences were analyzed by motif scan. Out of 820 sequences, 171 (21 %) were lacking one or more required blocks. This number was unexpectedly high, especially considering that PfamA is a high quality and manually curated database. In addition, note that Pfam used 65 sequences as a seed for their model, whereas our model was parameterized with 23 seed sequences. As shown graphically in Fig. 4 this additional analysis underlined the good performance of our model since the majority of these sequences obtained negative VD scores. PF00657 sequences annotated as GDSL by Pfam are divided into two groups, according to the presence or absence of required blocks (Additional file 4). These results show that the combination of motif scan and profile search provides an excellent tool for eliminating protein sequences that preserve protein family profile but lack specific motif(s). Additionally, our results indicate that the motif-based HMM parameterization used here could be successfully applied for the automatic annotation of GDSL enzymes in different plant proteomes or possibly in other large sequence datasets (e.g. metaproteomic datasets). Taking into account the economic importance of lipolytic enzymes in general and the need for fast identification over very large datasets we think that our model will be of general interest to scientists working in this field. Moreover, we believe that our motif scan approach could be successfully applied to the identification of the members of other protein families which exhibit low sequence similarity but possess conserved motifs. Therefore, we provide a web-based motif HMM scan application which uses a graphical interface and where users can choose between Viterbi decoding and Posterior decoding (http://compbio.math.hr).

### PD/VD combination for reduction of false positives

While analyzing predicted GDSL blocks we observed that for the great majority of identified GDSL sequences PD and VD predicted the same motifs (residue positions). In contrast, for the majority of negatives these two methods differed in predicted motifs. This could be ascribed to the fact that PD usually did not return a set of consecutive residues if GDSL motifs were not present (Methods). Starting with the assumption that PD and VD in combination might decrease the number of false positives, we developed the PD/VD protocol. As described in Methods, sequences in which VD and PD did not predict the same positions for all three motifs were removed from the output (Fig. 1). For example, by applying PD/VD protocol to the *A. thaliana* proteome some of the highly ranked sequences which did not possess all three required Blocks were removed, without losing any positive sequences (Fig. 2c, sequences marked with black arrows). Other combinations (VD/PSSM and PSSM/PD) did not perform as well and were not used in further analyses.

**Fig. 5:**
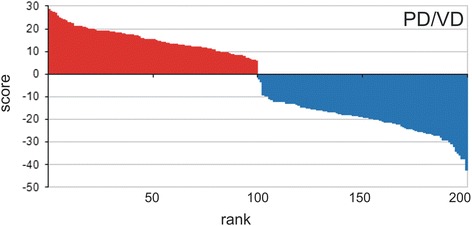
PD/VD performance on the test sample. All 100 plant GDSL sequences (red bars) obtained positive PD/VD scores (100 % sensitivity), while 100 randomly selected negatives (blue bars) obtained negative scores (100 % specificity)

Starting with this observation, we firstly evaluated PD/VD performance using a simple test sample consisting of 100 GDSL sequences identified in various land plant proteomes and 100 protein sequences without GDSL blocks randomly selected from the Swiss-Prot list of manually annotated and reviewed proteins. The PD/VD protocol achieved 100 % sensitivity and specificity taking again the Viterbi score zero as a threshold (Fig. 5). As a proof of concept, this result showed that the combination of PD and VD could potentially be used to decrease the number of false positives form VD output lists of large data sets.

Secondly, to test our protocol in a more realistic setting, PD/VD was applied to the previously analyzed proteomes (*A. thaliana*, *O. sativa, S. bicolor, P. patens, V. vinifera* and *P. trichocarpa).* Output lists were visually validated and results are shown in Figs. 2d and 3b. In order to compare relative performance of PD/VD against VD alone we have defined two additional terms: true positives (TP) and true negatives (TN), in addition to FP and FN, taking the Viterbi zero score as a threshold. Namely, all sequences with the three required motifs (here labelled positives) which obtained positive Viterbi scores were classified as TP; sequences lacking the required motifs (here labelled negatives) with negative scores as TN; whereas positives with negative scores as FN and *vice versa* negatives with positive scores as FP. The results of these analyses are shown in Table 1. We calculated, for VD and PD/VD, false discovery proportion (FDP) - the proportion of false positives among all sequences which obtained positive scores, FDP = FP/(FP + TP); and sensitivity (S) - the proportion of correctly identified positive sequences, S = TP/(TP + FN). In this setting, where the overwhelming majority of sequences are negative, specificity is not a suitable measure, since the ratio TN/N is only marginally affected by the number of FPs. In such cases FDP can be used as an appropriate measure instead of specificity. As shown in Table 1, PD/VD decreased FDP in all cases and retained very high sensitivity which remained equal in 4/6 cases and only slightly decreased in two cases. Altogether, when PD/VD was applied, 41 % of all FPs were eliminated (29/49), i.e. FDP was decreased from 8.5 to 5.3 %. On the other hand, sensitivity decreased slightly, from 98.7 to 97.9 % (Table 1). The presented results show that in our setting the PD/VD protocol was successful in the elimination of false positives while slightly decreasing sensitivity. However, it has to be taken into account that the benefit of the PD/VD will strongly depend on the model parameterization and these results should be viewed as a proof of concept.

**Table 1:** Comparison of VD and PD/VD performance based on visual inspection of 300 of the top ranked sequences for each proteome

Organism	Method	TP	FP	FN	FDP (%)	S (%)
*A. thaliana*	VD	116	8	0	6.5	100.0
	PD/VD	116	4	0	3.3	100.0
*S. bicolor*	VD	105	6	0	5.4	100.0
	PD/VD	104	2	1	1.9	99.0
*O. sativa*	VD	90	11	1	10.9	98.9
	PD/VD	90	6	1	6.3	98.9
*P. patens*	VD	40	0	3	0.0	93.0
	PD/VD	37	0	6	0.0	86.0
*P. trichocarpa*	VD	95	14	3	12.8	96.9
	PD/VD	95	11	3	10.4	96.9
*V. vinifera*	VD	80	10	0	11.1	100.0
	PD/VD	80	6	0	7.0	100.0
**Σ208,298 sequences**	**VD**	**526**	**49**	**7**	**8.5**	**98.7**
	**PD/VD**	**522**	**29**	**11**	**5.3**	**97.9**

The total number of entries in six proteomes and the numbers of GDSL identified by VD and PD/VD as well as respective statistical measures are shown in bold. *TP* true positive, *FP* false positive, *FN* false negative, *S* sensitivity [S = TP/(TP + FN)], *FDP* false discovery proportion [FDP = FP/(FP + TP)]; TPs that were eliminated by PD/VD protocol were added to the FN group

### Scanning for GDSL motifs in lower plants

**Fig. 6:**
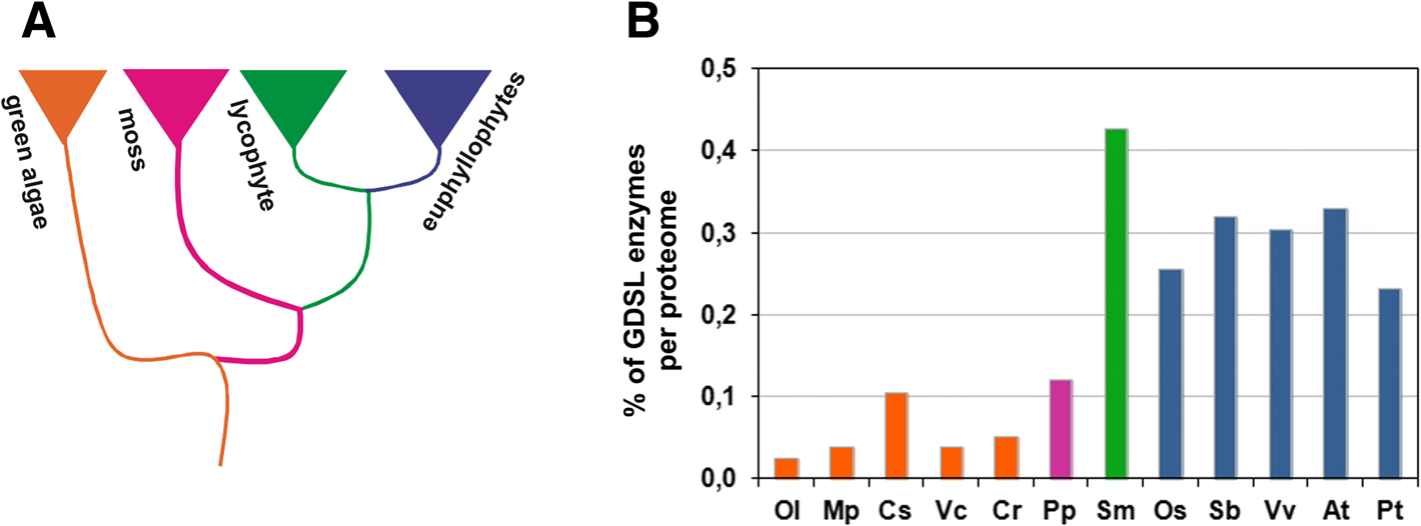
**a** Schematic representation of the evolution of plants. Green algae - orange, bryophyte - purple, lycophyte - green, euphyllophytes - dark blue. **b** Percentage of identified GDSL enzymes in analyzed plant proteomes. Ol - *Ostreococcus lucimarinus*; Mp - *Micromonas pusilla* RCC299; Cs - *Coccomyxa subellipsoidea* C-169; Vc - *Volvox carteri*; Cr - *Chlamydomonas reinhardtii*; Pp - *Physcomitrella patens*; Sm - *Selaginella moellendorffi*; Os - *Oryza sativa*; Sb - *Sorghum bicolor*; Vv - *Vitis vinifera*; Pt - *Populus trichocarpa*; At - *Arabidopsis thaliana*

We applied our motif-based HMM to analyze selected proteomes of lower plants. As described in the Background section, members of the GDSL protein family have been identified throughout the life kingdoms [4, 5, 9, 10, 34]. A significantly higher number (~10 X) of GDLS enzymes was observed in land plants in comparison to so far examined bacterial genomes (manuscript in preparation). The only analyzed representative of lower plants, *P. patens*, displayed approximately 50 % reduction in GDSL enzymes although its proteome size is comparable to those of euphyllophytes. Therefore we extended the GDSL motif scan to the only available lycophyte, *S. moellendorffii*. This organism represents an ancient lineage that diverged shortly after land plants evolved vascular tissues [45] (Fig. 6a). In addition, we analyzed five proteomes of green algae (*C. reinhardtii, C. subellipsoidea*, *M. pusilla*, *O. lucimarinus*, *V. carteri*) also known as “primitive plants” [46]. Due to the observed discrepancies in databases we have carefully examined proteomes from RefSeq and also from Phytozome. Since the latest versions of proteomes were still not released to RefSeq (S. Prochnik, jgi-phytozome, 2014, personal communication) here we show results of motif scanning performed on proteomes obtained from Phytozome (Additional file 5). As presented, the absolute number of GDSL enzymes is significantly lower in green algae (11 or less per proteome) than in land plants. However, in the lycophyte *S. moellendorffii* the number of GDSL enzymes was comparable to that of land plants (95). All output lists obtained in these analyses were examined carefully. Taking into account scores of positives from the previous section (Figs. 2 and 3) we have examined visually all sequences with a score around zero. In accordance with previous results all GDSL sequences from the six newly analyzed proteomes were ranked at the top of the output lists. Note that ten sequences from *S. moellendorffii*, although highly ranked, had a wrong prediction for one of the GDSL motifs positioned close to the beginning or end of the protein sequence (Block I or Block V). The reason that VD failed to identify the correct motif lies in the fact that the active site Ser in Block I (8/10) and His in Block V (2/10) were unexpectedly close to termini (3 residues from the N- or C- terminus respectively), whereas in our HMM the active site Ser in Block I is represented by the 6th match state and His in block V is followed by five match states (Additional file 2: Figure S2). Thus, it is not surprising that VD has missed these truncated blocks. Such cases were not observed during analysis of the other land plants and algae. Therefore, these sequences could be ascribed to an incomplete cDNA.

The percentages of GDSL of GDSL proteins identified by our motif scanning are shown in Fig. 6b. As shown, the only presently available lycophyte proteome suggests that the GDSL hydrolytic family expanded during the transition from bryophytes to vascular plants. We propose that this remarkable expansion of GDSL enzymes found in the lycophyte, *S. moellendorffii* with proteome size comparable to algae might have been important in the early evolution of developmental and metabolic processes unique to vascular plants [45].

### Phylogenetic analysis of GDSL enzymes

**Fig. 7:**
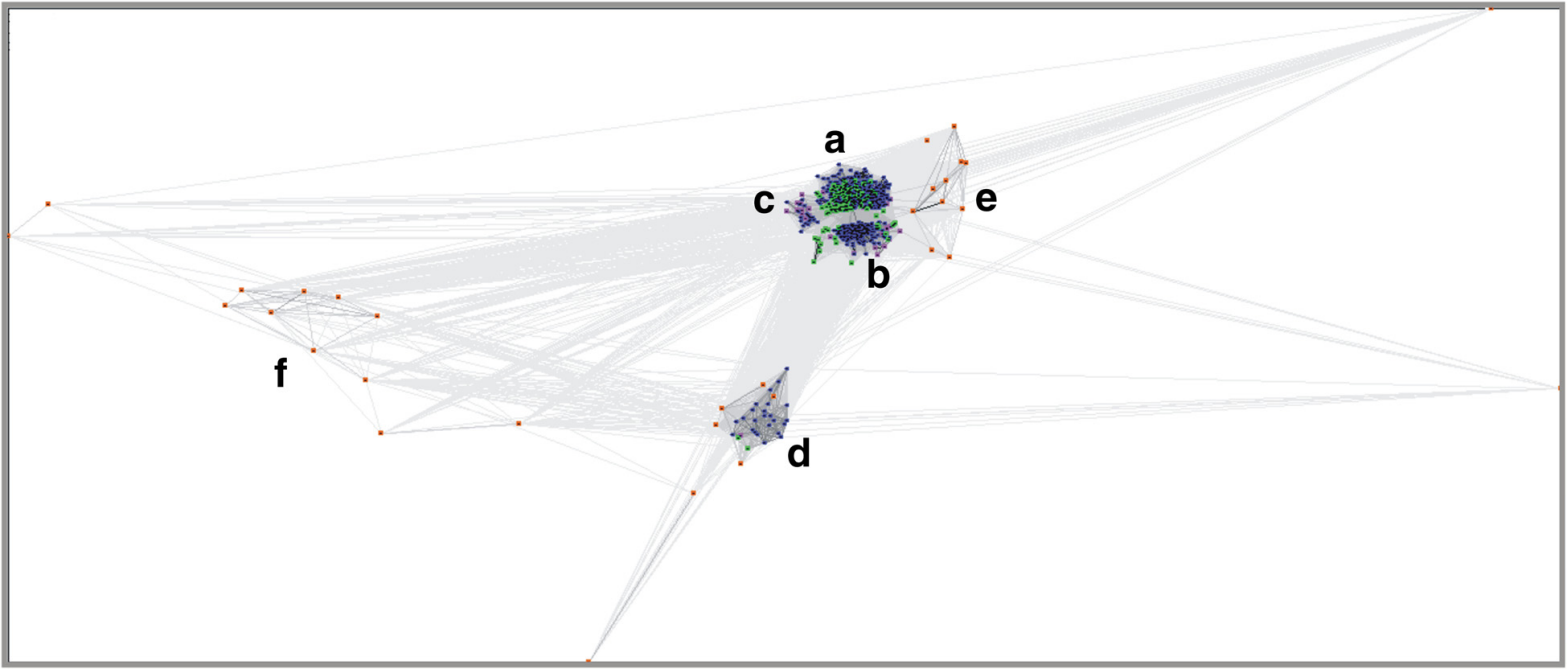
Cluster map of identified GDSL proteins. The CLANS analysis of all GDSL proteins displayed six distinct subfamilies, **a**-**f**. GDSL proteins from green algae are shown as orange dots, from bryophyte as purple dots, from lycophyte as green dots and from euphyllophytes as dark blue dots

To find evolutionary links between all GDSL sequences identified in this study, we additionally performed a phylogenetic analysis. Previous phylogenetic analysis of GDSL lipases from land plants showed the existence of two major subfamilies (A and B) and one minor subfamily (C) [9]. In this study we used our expanded list of GDSL enzymes from the land plants and from the lower plants. GDSL sequences were firstly analyzed by the Cluster Analysis of Sequences program (CLANS) [29]. As depicted in Fig. 7 all GDSL sequences from land plants were clustered into four well defined groups A, B, C, and a previously unreported group D. Two additional groups, E and F which we report here arise only from algal sequences. They are presently represented by a small number of sequences but accumulation of new algal genomes should eventually enrich these groups. As shown in Fig. 7, group E is positioned closer to groups A, B, and C that are formed only of GDSL sequences from land plants (moss, lycophytes and euphyllophytes). The positions of groups F and D in the cluster map indicate a distant relationship to other groups. Noteworthy, only group D contains GDSL proteins from all 12 analyzed plants, thus possibly indicating that they contribute to essential and common cellular function(s).

**Fig. 8:**
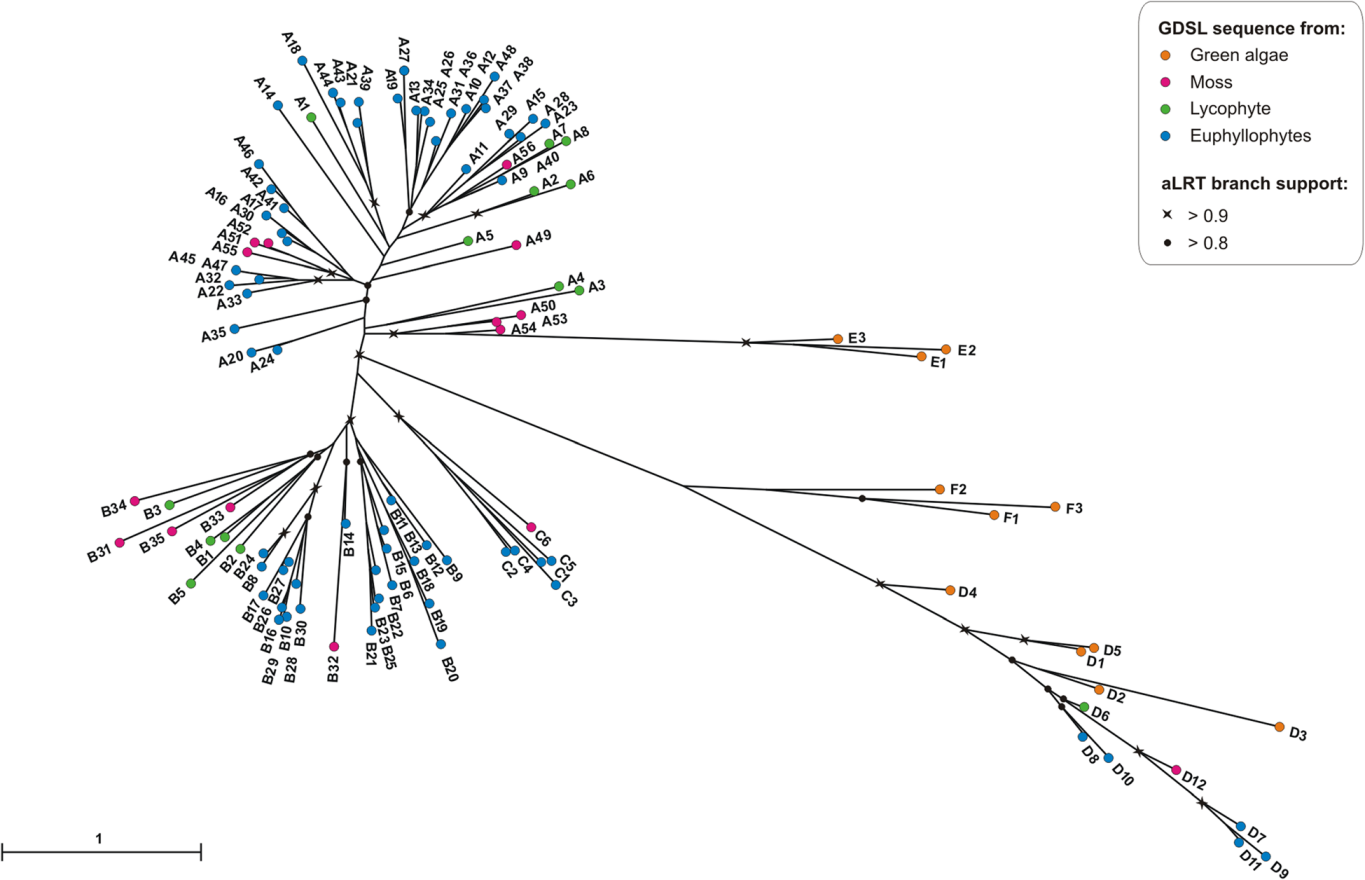
Unrooted ML tree of selected plant GDSL lipases

Phylogenetic analysis was performed with the number of sequences proportional to the CLANS group size (Additional file 6), as described in Methods. In accordance with the CLANS result the ML tree (Fig. 8) showed a clear separation of groups A, B, C, D, E, and F and provided a better insight into their evolutionary relationships. Most of the GDSL sequences found in land plants (~100 per proteome) fell into two main phylogenetic groups (A or B). As depicted in Fig. 8 group A is polyphyletic whereas groups B - F are monophyletic. Groups D, E and F identified in this study formed a well supported but divergent clades. GDSL sequences belonging to the algal group F are most closely related to group D, while algal sequences from the group E form a clade related to the A group.

As described, the highest proportion of GDSL enzymes per proteome was observed for *S. moellendorffii* (Fig. 6b)*.* Its GDSL sequences were clustered within groups A, B, and D. However, this lycophyte is lacking GDSL sequences belonging to group C. In plant evolution mosses diverged earlier from the common ancestor of the lycophytes and euphyllophytes. Since group C contains GDSL sequences from the moss and euphyllophytes we assume that these genes were lost from the lycophyte lineage. Further conclusions on the early evolution of subfamilies A – D can be drawn from the distribution of GDSL sequences of the moss, *P. patens.* Their presence in these subfamilies implies that the observed subfamilies A - D already existed before the moss lineage diverged from the ancestor of vascular plants. Thus, the subsequent increase in the number of GDSL proteins occurred in the already existing subfamilies, as described above, predominantly A and B. As proposed earlier, the expansion of GDSL sequences in land plants could be explained by gene duplication and their retention by functional specialization [9].

### Analysis of GDSL motifs

**Fig. 9:**
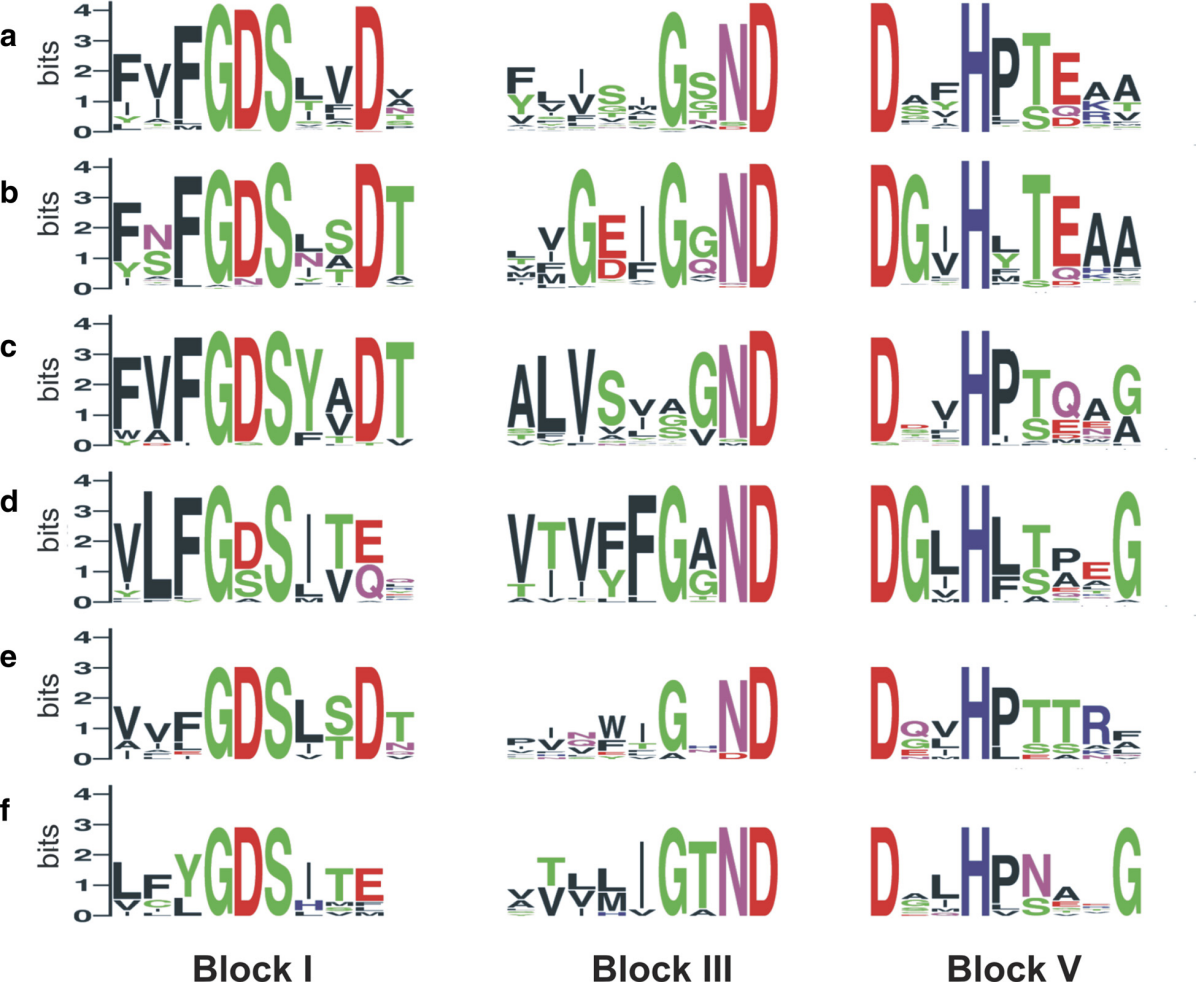
Conserved Blocks from plant GDSL subfamilies **a**-**f**. Blocks were obtained from MSA of 374, 197, 27, 32, 11 and 10 GDSL sequences for subfamilies (**a**), (**b**), (**c**), (**d**), (**e**) and (**f**), respectively, and visualized by WebLogo [53]. Polar amino acids G, S, T, Y and C are green, neutral Q and N are purple, basic K, R and H are blue, acidic D and E are red, and hydrophobic A, V, L, I, P, W, F and M are black

We have aligned all sequences belonging to particular CLANS groups and have visualized group-specific motifs by WebLogo (Fig. 9). When compared to the blocks of seed sequences (Additional file 2: Figure S2) we found previously undescribed variations in all three motifs.

As shown in Fig. 9, motif sequences exhibit specific variations that correlate well with the depicted phylogenetic groups. For example, related subfamilies A, B, C and E, possess in the Block I a highly conserved residue **D** at position 9 whereas Block I from the more divergent subfamilies D and F most often contains the sequence **GDSITE**. Additional variations between closely related subfamilies were observed. An interesting deviation in Block I, namely **GSS** instead of the **GDS** has often been observed in group D (11/32 sequences). Some poorly represented variations of Block I (not visible on WebLogo) were also found in groups A, B, C, and D (e.g. GNS, GAS, ADS, etc.). **GSS** and **GNS** motifs were reported recently in GDSL hydrolases from two closely related bacteria, *Leptospira borgpetersenii* and *L. interrogans* [47]. We have also found over 60 GDSL sequences possessing the **GSS** motif in other plants (data not shown). A broad distribution of these particular motifs in bacteria and plants possibly implies their biological importance and functional specialization. Other blocks also exhibited group specific variations. The most common sequence in Block III is **G**x**ND**, however each group displayed characteristic amino acid residues at specific positions (Fig. 9). Similarly, Block V contains a conserved **D**xx**H** sequence, whereas other positions show much higher variations. Although Asp (**D**) is highly conserved in plants, analyses of bacterial GDSL family showed that this residue could often be substituted with W, N or E [34, 35, 48]. In conclusion, each phylogenetic group reported here shows some motif preferences. So far, only a few structural studies of GDSL hydrolases have been reported [8, 48–51]. In spite of low sequence similarity the overall fold of GDSL enzymes proved to be conserved. However, diversity of loop conformations in the substrate binding domains are found to be important in defining geometry of the active site pocket which consequently determines the substrate specificities of GDSL enzymes [8, 52]. We applied the Ali2D program to analyze secondary structure topology of all representatives used in the phylogenetic analyses. Globally the results suggest that secondary structure content is preserved although variations in α helices and β strands among the groups could be observed. The relative location of the motifs possessing the key amino acid residues is rather conserved in all analyzed GDSL enzymes (Additional file 7). However, occurrence of the insertions/gaps between predicted secondary structure elements suggests that variations in the binding pockets could be expected even for members of the same group. Therefore, the extent by which these motif variations influence the enzymes’ structural and biochemical properties remains a major challenge for future studies.

Altogether, phylogenetic analyses, presented motif variations and secondary structure predictions underscore the correct identification of GDSL sequences, thus indicating the high sensitivity and specificity (i.e. FDP) of our motif-HMM model.

## Conclusions

In this study we successfully applied an HMM-based motif scanning approach as a solution for the improved annotation of sequences belonging to the protein family with members sharing low sequence similarity. Our revised list of GDSL enzymes enabled new insight to the phylogenetic relations of GDSL family members in the plant kingdom. Novel subfamilies and corresponding motif-variations were found. We expect that our approach could also be applied to other families with low overall sequence similarity and multiple conserved motifs, thereby reducing the number of wrongly annotated sequences in the era of massive high-throughput technologies.

## Additional files

Additional file 1:
**Schematic representation of the motif-HMM used in this study.** (DOCX 101 kb) Additional file 2: Seed dataset. **Table S1**, Experimentally characterized GDSL sequences used for seed alignment. **Figure S1**, Alignment of seed sequences. **Figure S2**, Conserved Blocks derived from the seed sequences. (DOCX 603 kb) Additional file 3:
**Revised list of GDSL sequences.** Analyses of GDSL sequences from different proteomes are given in separate sheets. In each sheet, table A shows GDSL sequences from the RefSeq proteomes identified in this study; table B shows previously reported GDSL sequences not identified in our study but traced in RefSeq via provided gene loci [9]; and table C shows previously reported GDSL sequences [9] that could not be traced since standard gene identifiers were not provided. (XLSX 59 kb) Additional file 4:
**List of sequences from Pfam family PF00657.** GDSL lipases are shown in red while sequences missing one or more blocks are in blue. (XLSX 31 kb) Additional file 5:
**List of GDSL sequences from lycophyte** ***S. moellendorffii*** **and selected green algae identified here by motif scanning methods.** Lists of GDSL sequences from different proteomes are given in separate sheets. (XLSX 49 kb) Additional file 6:
**List of GDSL sequences used for the phylogenetic analysis.** (XLSX 15 kb) Additional file 7:
**Secondary structure predictions of selected GDSL hydrolases belonging to CLANS groups A-F.** Ali2D program (http://toolkit.tuebingen.mpg.de/ali2d) was used for alignment-based secondary structure predictions. WebLogo was used to visualize positions of conserved Blocks I, III and V with catalytic residues. (PDF 437 kb)
