# Supplementary figures and images for: An effective approach for annotation of protein families with low sequence similarity and conserved motifs: identifying GDSL hydrolases across the plant kingdom
Source: BMC Bioinformatics. 2016 Feb 18;17:91. doi: 10.1186/s12859-016-0919-7 (PMC4757993; doi:10.1186/s12859-016-0919-7)

**
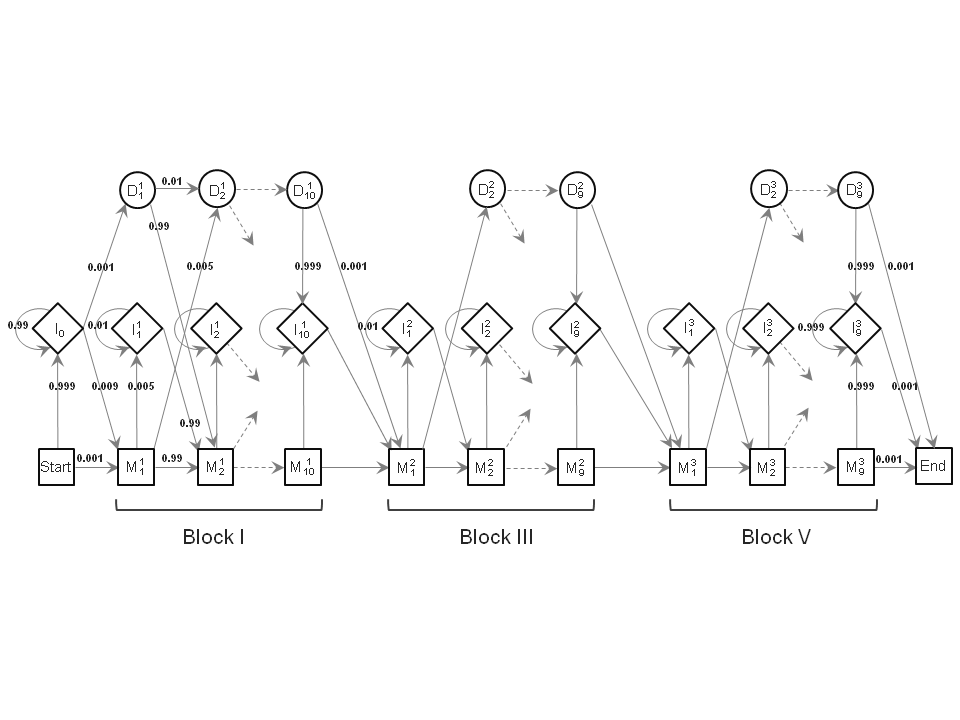
**

**The motif-HMM used in this study.** Transition probabilities are shown.

Supplement: Additional file 1: — Schematic representation of the motif-HMM used in this study. (DOCX 101 kb) [file 12859_2016_919_MOESM1_ESM.docx]
