# Supplementary material for: An effective approach for annotation of protein families with low sequence similarity and conserved motifs: identifying GDSL hydrolases across the plant kingdom
Source: BMC Bioinformatics. 2016 Feb 18;17:91. doi: 10.1186/s12859-016-0919-7 (PMC4757993; doi:10.1186/s12859-016-0919-7)
Supplement: Additional file 2: — Seed dataset. Table S1, Experimentally characterized GDSL sequences used for seed alignment. Figure S1, Alignment of seed sequences. Figure S2, Conserved Blocks derived from the seed sequences. (DOCX 603 kb) [file 12859_2016_919_MOESM2_ESM.docx]

**Table S1** **Experimentally characterized GDSL sequences used for seed alignment**

|  | Enzyme | UniProt number | Sequence | Reference |
| --- | --- | --- | --- | --- |
| 1. | Lipase from *Streptomyces rimosus* (LIP_STRRM) | Q93MW7 | MRLSRRAATASALLLTPALALFGASAAVSAPRIQATDYVALGDSYSSGVGAGSYDSSSGSCKRSTKSYPALWAASHTGTRFNFTACSGARTGDVLAKQLTPVNSGTDLVSITIGGNDAGFADTMTTCNLQGESACLARIAKARAYIQQTLPAQLDQVYDAIDSRAPAAQVVVLGYPRFYKLGGSCAVGLSEKSRAAINAAADDINAVTAKRAADHGFAFGDVNTTFAGHELCSGAPWLHSVTLPVENSYHPTANGQSKGYLPVLNSAT | [[1](#_ENREF_1)] |
| 2. | Esterase for *Streptomyces scabies* (ESTA_STRSC) | P22266 | MSSAMRKTTNSPVVRRLTAAAVALGSCLALAGPAGSAGAAPADPVPTVFFGDSYTANFGIAPVTNQDSERGWCFQAKENYPAVATRSLADKGITLDVQADVSCGGALIHHFWEKQELPFGAGELPPQQDALKQDTQLTVGSLGGNTLGFNRILKQCSDELRKPSLLPGDPVDGDEPAAKCGEFFGTGDGKQWLDDQFERVGAELEELLDRIGYFAPDAKRVLVGYPRLVPEDTTKCLTAAPGQTQLPFADIPQDALPVLDQIQKRLNDAMKKAAADGGADFVDLYAGTGANTACDGADRGIGGLLEDSQLELLGTKIPWYAHPNDKGRDIQAKQVADKIEEILNR | [[2](#_ENREF_2)] |
| 3. | Lipase 1 from *Streptomyces coelicolor* (LIP1_STRCO) | Q9S2A5 | MRRFRLVGFLSSLVLAAGAALTGAATAQAAQPAAADGYVALGDSYSSGVGAGSYISSSGDCKRSTKAHPYLWAAAHSPSTFDFTACSGARTGDVLSGQLGPLSSGTGLVSISIGGNDAGFADTMTTCVLQSESSCLSRIATAEAYVDSTLPGKLDGVYSAISDKAPNAHVVVIGYPRFYKLGTTCIGLSETKRTAINKASDHLNTVLAQRAAAHGFTFGDVRTTFTGHELCSGSPWLHSVNWLNIGESYHPTAAGQSGGYLPVLNGAA | [[3](#_ENREF_3), [4](#_ENREF_4)] |
| 4. | Lipase 2 from *Streptomyces coelicolor* (LIP2_STRCO) | Q93J06 | MPKPALRRVMTATVAAVGTLALGLTDATAHAAPAQATPTLDYVALGDSYSAGSGVLPVDPANLLCLRSTANYPHVIADTTGARLTDVTCGAAQTADFTRAQYPGVAPQLDALGTGTDLVTLTIGGNDNSTFINAITACGTAGVLSGGKGSPCKDRHGTSFDDEIEANTYPALKEALLGVRARAPHARVAALGYPWITPATADPSCFLKLPLAAGDVPYLRAIQAHLNDAVRRAAEETGATYVDFSGVSDGHDACEAPGTRWIEPLLFGHSLVPVHPNALGERRMAEHTMDVLGLD | [[4](#_ENREF_4)] |
| 5. | Esterase from *Pseudomonas aeruginosa* (EST_PSEAE) | Q9HZY8 | MRALLLSGCLALVLLTQQAAAQTLLVVGDSISAALGLDTSQGWVALLQKRLADEGYDYRVVNASISGDTSAGGLARLPALLAEEKPALVVIELGGNDGLRGMAPAQLQQNLASMAQKARAEGAKVLLLGIQLPPNYGPRYIEAFSRVYGAVAAQEKTALVPFFLEGVGGVQGMMQADGIHPALAAQPRLLENVWPTLKPLL | [[5](#_ENREF_5)] |
| 6. | Rhamnogalacturonan acetylesterase from *Aspergillus aculeatus* (RHA1_ASPAC) | Q00017 | MKTAALAPLFFLPSALATTVYLAGDSTMAKNGGGSGTNGWGEYLASYLSATVVNDAVAGRSARSYTREGRFENIADVVTAGDYVIVEFGHNDGGSLSTDNGRTDCSGTGAEVCYSVYDGVNETILTFPAYLENAAKLFTAKGAKVILSSQTPNNPWETGTFVNSPTRFVEYAELAAEVAGVEYVDHWSYVDSIYETLGNATVNSYFPIDHTHTSPAGAEVVAEAFLKAVVCTGTSLKSVLTTTSFEGTCL | [[6](#_ENREF_6)] |
| 7. | Arylesterase from *Vibrio mimicus* (ESTE_VIBMI) | Q07792 | MIRLLSLVLFFCLSAASQASEKLLVLGDSLSAGYQMPIEKSWPSLLPDALLEHGQDVTVINGSISGDTTGNGLARLPQLLDQHTPDLVLIELGANDGLRGFPPKVITSNLSKMISLIKDSGANVVMMQIRVPPNYGKRYSDMFYDIYPKLAEHQQVQLMPFFLEHVITKPEWMMDDGLHPKPEAQPWIAEFVAQELVKHL | [[7](#_ENREF_7)] |
| 8. | Acyl-CoA thioesterase I from *Escherichia coli* (TESA_ECOLI) | P0ADA1 | MMNFNNVFRWHLPFLFLVLLTFRAAAADTLLILGDSLSAGYRMSASAAWPALLNDKWQSKTSVVNASISGDTSQQGLARLPALLKQHQPRWVLVELGGNDGLRGFQPQQTEQTLRQILQDVKAANAEPLLMQIRLPANYGRRYNEAFSAIYPKLAKEFDVPLLPFFMEEVYLKPQWMQDDGIHPNRDAQPFIADWMAKQLQPLVNHDS | [[8](#_ENREF_8)] |
| 9. | Esterase from *Pseudomonas aeruginosa* (ESTA_PSEAE) | O33407 | MIRMALKPLVAACLLASLSTAPQAAPSPYSTLVVFGDSLSDAGQFPDPAGPAGSTSRFTNRVGPTYQNGSGEIFGPTAPMLLGNQLGIAPGDLAASTSPVNAQQGIADGNNWAVGGYRTDQIYDSITAANGSLIERDNTLLRSRDGYLVDRARQGLGADPNALYYITGGGNDFLQGRILNDVQAQQAAGRLVDSVQALQQAGARYIVVWLLPDLGLTPATFGGPLQPFASQLSGTFNAELTAQLSQAGANVIPLNIPLLLKEGMANPASFGLAADQNLIGTCFSGNGCTMNPTYGINGSTPDPSKLLFNDSVHPTITGQRLIADYTYSLLSAPWELTLLPEMAHGTLRAYQDELRSQWQADWENWQNVGQWRGFVGGGGQRLDFDSQDSAASGDGNGYNLTLGGSYRIDEAWRAGVAAGFYRQKLEAGAKDSDYRMNSYMASAFVQYQENRWWADAALTGGYLDYDDLKRKFALGGGERSEKGDTNGHLWAFSARLGYDIAQQADSPWHLSPFVSADYARVEVDGYSEKGASATALDYDDQKRSSKRLGAGLQGKYAFGSDTQLFAEYAHEREYEDDTQDLTMSLNSLPGNRFTLEGYTPQDHLNRVSLGFSQKLAPELSLRGGYNWRKGEDDTQQSVSLALSLDF | [[9](#_ENREF_9)] |
| 10. | Esterase from *Pseudomonas putida* (ESTP_PSEPK) | Q88QS0 | MRKAPLLRFTLASLALACSQALAGPSPYSTLIVFGDSLADAGQFPDLVGGTPGARFTNRDADGNFAPVSPMILGGRLGVAPGDLNPSTSVGIQPDGNNWAVGGYTTQQILDSITTTSETVIPPGNPNAGLVLRERPGYLANGLRADPNALYYLTGGGNDFLQGLVNSPADAVAAGARLAASAQALQQGGARYIMVWLLPDLGQTPNFSGTPQQNPLSLLSAAFNQSLISQLGQIDAQIIPLNIPLLLSEALASPSQFGLASDQNLVGTCYSGDSCVENPVYGINGTTPDPTKLLFNDSVHPTIAGQQLIADYAYSILAAPWELTLLPEMAHASLRAHQDELRNQWQTPWQAVGQWQAFVASGAQDLDFDGQHSAASGDGRGYNLTVGGSYRLNDAWRLGLAGGANRQKLEAGEQDSDYKLNSYMASAFAQYRQDRWWADAALTAGHLDYSDLKRTFALGVNDRSEKGDTDGEAWAMSGRLGYNLAADTSNWQLAPFISADYARVKVDGYDEKSGRSTALGFDDQERTSRRLGVGLLGSVQVLPSTRLFAEVAQEHEFEDDEQDVTMHLTSLPANDFTLTGYTPHSDLTRASLGVSHELVAGVHLRGNYNWRKSDELTQQGISVGVSVDF | [[5](#_ENREF_5)] |
| 11. | EstA from *Serratia liquefaciens* (Q7X171_SERLI) | Q7X171 | MPLKITCMPRPAALAVALLCSVTLPAQAYDQLYVFGDSLSDTGNNGRFTYDGSQHLLYDEALAQRIGAALVASDNGGENYAAGGAVAVPGLNPADNTQDQVQSYLNRVNGQADGDGLYIHWIGGNDLAAAALNAATAPGVAYNSAAAAAAQVHSLLNAGAGTVIVPTVPNIGSTPQLMELIIQQALSPVQGAAIQAAYATLNSVATPDNASRTQAIHAALAAAAKQGSAIPQVQQAIATQLIAAYDSLSAQAAQLTDFYNQSEDRLLAQGGGNIVRVDVNKLFAEAIANPAQFGFANTAGMACPPGISSASRSDMPASTSASLSVLDHFHPERQAHLLIANYIQAVLDGPAQVVALNQGDGGGMRQGAPRSNSPSKQLPRDNRRGSLGVFGGYAGQHYDYADNRAAGDGNATTHNLTVGVDYQLTDGWLIGALIAGSNDDQHPSSRFDYKARGLLLSAFSSLALFEHGWVNADLHYATMDYDDIRRSMRLGPLTRTENGSTTGKQWGARVTAGYDFPIASYLTTGPVAQFAWDYSRVSGYSEDGDDSTAMRFNDQTYHSQIGALGWRLDTQFGVFNPYAEVSYQHQFGDDVYRAGGGLKSTQTSFTRDSAGQDKNWVDVTLGANMPLTDRVSAFATVSQTGGLSSGEQFMYNVGVSARF | [[10](#_ENREF_10)] |
| 12. | Lipase 1 from *Photorhabdus luminescens* (LIP1_PHOLU) | P40601 | MKRSFIFAPGMLALSISAISNAHAYNNLYVFGDSLSDGGNNGRYTVDGINGTESKLYNDFIAQQLGIELVNSKKGGTNYAAGGATAVADLNNKHNTQDQVMGYLASHSNRADHNGMYVHWIGGNDVDAALRNPADAQKIITESAMAASSQVHALLNAGAGLVIVPTVPDVGMTPKIMEFVLSKGGATSKDLAKIHAVVNGYPTIDKDTRLQVIHGVFKQIGSDVSGGDAKKAEETTKQLIDGYNELSSNASKLVDNYNQLEDMALSQENGNIVRVDVNALLHEVIANPLRYGFLNTIGYACAQGVNAGSCRSKDTGFDASKPFLFADDFHPTPEAHHIVSQYTVSVLNAPYRVMLLTNANNVPVKGALASLDGRLQQLRNVDNEQGKLGVFGGYSGNHSHTLTLGSDYQIMDNILLGGMISRYQDNSSPADNFHYDGRGYVFTAYGLWRYYDKGWISGDLHYLDMKYEDITRGIVLNDWLRKENASTSGHQWGGRITAGWDIPLTSAVTTSPIIQYAWDKSYVKGYRESGNNSTAMHFGEQRYDSQVGTLGWRLDTNFGYFNPYAEVRFNHQFGDKRYQIRSAINSTQTSFVSESQKQDTHWREYTIGMNAVITKDWGAFASISRNDGDVQNHTYSFSLGVNASF | [[11](#_ENREF_11)] |
| 13. | Thermolabile hemolysin from *Vibrio parahaemolyticus* (HLT_VIBPA) | Q99289 | MMKKTITLLTALLPLASAVAEEPTLSPEMVSASEVISTQENQTYTYVRCWYRTSYSKDDPATDWEWAKNEDGSYFTIDGYWWSSVSFKNMFYTNTSQNVIRQRCEATLDLANENADITFFAADNRFSYNHTIWSNDAAMQPDQINKVVALGDSLSDTGNIFNASQWRFPNPNSWFLGHFSNGFVWTEYIAKAKNLPLYNWAVGGAAGENQYIALTGVGEQVSSYLTYAKLAKNYKPANTLFTLEFGLNDFMNYNRGVPEVKADYAEALIRLTDAGAKNFMLMTLPDATKAPQFKYSTQEEIDKIRAKVLEMNEFIKAQAMYYKAQGYNITLFDTHALFETLTSAPEEHGFVNASDPCLDINRSSSVDYMYTHALRSECAASGAEKFVFWDVTHPTTATHRYVAEKMLESSNNLAEYRF | [[12](#_ENREF_12)] |
| 14. | Phosphatidylcholine-sterol acyltransferase from *Aeromonas hydrophila* (GCAT_AERHY) | P10480 | MKKWFVCLLGLVALTVQAADSRPAFSRIVMFGDSLSDTGKMYSKMRGYLPSSPPYYEGRFSNGPVWLEQLTNEFPGLTIANEAEGGPTAVAYNKISWNPKYQVINNLDYEVTQFLQKDSFKPDDLVILWVGANDYLAYGWNTEQDAKRVRDAISDAANRMVLNGAKEILLFNLPDLGQNPSARSQKVVEAASHVSAYHNQLLLNLARQLAPTGMVKLFEIDKQFAEMLRDPQNFGLSDTENACYGGSYVWKPFASRSASTDSQLSAFNPQERLAIAGNPLLAQAVASPMAARSASTLNCEGKMFWDQVHPTTVVHAALSEPAATFIESQYEFLAH | [[13](#_ENREF_13)] |
| 15. | Anter-specific proline-rich protein from *Arabidopsis thaliana* (APG_ARATH) | P40602 | MKRSSLVDSCSYSRIFRSIFCLLSFCIFFLTTTNAQVMHRRLWPWPLWPRPYPQPWPMNPPTPDPSPKPVAPPGPSSKPVAPPGPSPCPSPPPKPQPKPPPAPSPSPCPSPPPKPQPKPVPPPACPPTPPKPQPKPAPPPEPKPAPPPAPKPVPCPSPPKPPAPTPKPVPPHGPPPKPAPAPTPAPSPKPAPSPPKPENKTIPAVFFFGDSVFDTGNNNNLETKIKSNYRPYGMDFKFRVATGRFSNGMVASDYLAKYMGVKEIVPAYLDPKIQPNDLLTGVSFASGGAGYNPTTSEAANAIPMLDQLTYFQDYIEKVNRLVRQEKSQYKLAGLEKTNQLISKGVAIVVGGSNDLIITYFGSGAQRLKNDIDSYTTIIADSAASFVLQLYGYGARRIGVIGTPPLGCVPSQRLKKKKICNEELNYASQLFNSKLLLILGQLSKTLPNSTFVYMDIYTIISQMLETPAAYGFEETKKPCCKTGLLSAGALCKKSTSKICPNTSSYLFWDGVHPTQRAYKTINKVLIKEYLHVLSK | [[14](#_ENREF_14)] |
| 16. | Esterase from *Xanthomonas vesicatoria* (Q7X4K7_9XANT) | Q7X4K7 | MASTLRPIRSLMAVAIALAASPAMADSAFDQTVFFGDSLTDSGYYNPLLPAASRAVTGKFTTNPGWVWAEYVGDHFGTNAAPNGNGQTGDNYAAGGARIQASSVSALGAAPSVTSQVNTYLAANGGQANPNALYTVWGGANDLLAAATAPAQAQTIIGSAVTAQVGAVGALQAAGARYVMVPTIPDVGITPRFRAGGAAAMAQGTAAATAYNTALFNGLQSAGLRVIPVDTFHILQEVVADPGIYGFSNVTGTACNPALALPACNPTSLVAANAPNTYVFADGIHPTTATHQILGQYAISLLEAPRLQQVLTRSAQAGGRARADQVAWHLDGKPEADGLRWWGSVRGDIQRYDDADLYDGMAPAGLFGVDWTAGDLVFGGFAGFGRMDADFGNRNGSFKQDDTTLGGFVGWYTGPVWVNAQVSYSWLSYDVDREVQLGPATRVHSGAPDGSNLTAAVNAGYSLGEGNVKYGPVVGLTWQKLKLDGYTESNASSTALGYADQDIDSLVGRIGFQVRLDGAPVKPYLQATYDHEFKDGTEASAWLQSMPEVGMYTVPGQNFDRNYATVVLGARTGIWGLQSNIGLSTTTAQRSARDATVFVNFSGNF | [[15](#_ENREF_15)] |
| 17. | GDSL-lipase from *Chenopodium rubrum* (Q7Y055_CHERU) | Q7Y055 | MAKITPKTTKMLPTLALFAAILFLSLSPSNAQYKPPLFVFGDSLYDDGMTLHNGVKGAGAEFWPYGETYFKKPAGRYSDGRLIPDFIVQFAGLPFLQPYLLPGIKDFTKGINFASAGACVLVETRPQTINLKRQVDYFLQMVQKLKQQVGDAQANQLLSEAVYLFNIAGNDYVTLLQKNVKKLPLSNFKRNRQMNMILGNLTIHIKTIYNQGGRKFAFQNLGPLGCMPSMKYMLAYKGTCAPEPQELAKMHNAKFAALAKRLQSNLPGFKYSIYDFYTSLYLRVLYGSRYGFRESQTACCGSGSYNGDFTCQKKDQSFSVCSNPNEYLWFDAAHPTDKANQAFSKEFWSGGSNLVSPYNLQNLFAAK | - |
| 18. | Nectar protein 1 from *Jacaranda mimosifolia* (B0FTZ8_JACMI) | B0FTZ8 | KPQVPCFFIFGDSLVDNGNNNNIQSLARANYLPYGVDFPDGPTGRFSNGKTTVDVIAELLGFDDYIPPYASASGDQILRGVNYASAAAGIRSETGQQLGARIDFTGQVNNYKNTVAQVVDILGDEDSAANYLSKCIYSVGVGSNDYLNNYFMPLYYSSGRQYSPEQYSDLLIQQYSEQIRTLYNYGARKFSLIGVGQIGCSPNALAQNSPDGSTCIRRINDANQMFNNKLRALVDELNNGAQDAKFIYINAYGIFQDLIDNPSAFGFRVTNAGCCGVGRNNGQITCLPMQTPCQNRDEYLFWDAFHPTEAANVVVGRRSYRAEKASDAYPFDIQRLAQL | [[16](#_ENREF_16)] |
| 19. | GDSL esterase/lipase 1 from *Arabidopsis thaliana* (GLIP1_ARATH) | Q9FLN0 | MENSQLVSITFLAYTIIISIGSINCIDNNNLVTNQSALFVFGDSVFDAGNNNYIDTLSSVRSNYWPYGQTTFKSPTGRVSDGRLIPDFIAEYAWLPLIPPNLQPFNGNSQFAYGVNFASGGAGALVGTFSGLVINLRTQLNNFKKVEEMLRSKLGDAEGKRVISRAVYLFHIGLNDYQYPFTTNSSLFQSISNEKYVDYVVGNMTDVFKEVYNLGGRKFGILNTGPYDCAPASLVIDQTKIRSCFQPVTELINMHNEKLLNGLRRLNHELSGFKYALHDYHTSLSERMNDPSKYGFKEGKKACCGSGPLRGINTCGGRMGLSQSYELCENVTDYLFFDPFHLTEKANRQIAELIWSGPTNITGPYNLKALFELN | [[17](#_ENREF_17)] |
| 20. | GDSL-lipase protein from *Capsicum annuum* (Q08ET5_CAPAN) | Q08ET5 | MMMGARELVRKWIVMYVVVLLGLNLWGYYGVNAQQVPCYFIFGDSLVDNGNNNNIQSLARANYLPYGIDYPGGPTGRFSNGKTTVDVIAELLGFEDYIPPYADARGEDILKGVNYASAAAGIRDETGQQLGARIPFGGQVNNYRDTVQQVVQILGNEDSAATYLSKCVYPIGLGSNDYLNNYFMPMYYSTGRQYNPEQYADILIQQYTQHLKTLYDYGARKFVLIGVGQIGCSPNALAQNSADGRTCAQNINAANQLFNNRLRGLVDEFNGNTPDAKFIYINAYDIFQDLIDNPSAFGFRVTNAGCCGVGRNNGQITCLPLQNPCPNRDEYLFWDAFHPGEAANTIVGRRSYRAERSSDAYPFDIQHLAQL | [[18](#_ENREF_18)] |
| 21. | GDSL esterase from uncultured marine bacterium (B7X9Y2_9BACT) | B7X9Y2 | MPRFSALKTSVIRCLTAVALCLVFAGAAARDAPVLLVLGDSLSAAYGMPLSRGWVSLLEQRLRDANRPWRVVNASISGDTTSGALKRLPKLLELHAPEVVIIELGGNDGLQGKPLDTIASNLQGLISVVRGAGAQPALVGMRIPPNYGRYYTGEFERLYEQIAEREEVPLLRFGLEGLASARGMMQEDGIHPAPEAQARMLDSLWPDLNAELLPLEQGAPLDH | [[19](#_ENREF_19)] |
| 22. | GDSL esterase/lipase from *Carica papaya* (GDL1_CARPA) | P86276 | MEKPSGQFLGLSLLLLPLLLPISCNAQQLFIFGDSLYDNGNKPFLATDVPSTFWPYGLSIDFPNGRWSDGRIVPDFIAEFLGIPFPPPVLDRSANFSSGVTFATADATILGTPPQTLTLGDQVKAFAQIKSTWTDAQRQKGIYMFYIGANDYLNYTNANLNATAQQQEAFVSQVIAKLKDQLLAIYGLGGRKFAFQNLAPLGCLPIVKQDFKTGNFCLPLASNLAAQHNQLLSETLENLSETLDGFNYIIYDYFNSSLRRMARPNNYGYFTTNLACCGTGSHDAFGCGFKNVHSNLCSYQRGYMFFDGRHNAEKTNEAVAHLIFSADPSVVFPMNLRELFVHP | [[20](#_ENREF_20)] |
| 23. | Lipase 2 from *Brassica napus* (Q3ZFI4_BRANA) | Q3ZFI4 | MASSLKKLITSFLLFFFYTIIVASSEPSCRRYKSIISFGDSIADTGNYLHLSDVNHPPQAAFLPYGETFFSVPTGRDSDGRLIIDFIAEFLGLPYVPPYFGSQNVSFEQGVNFAVYGATALDRAFFIEKGIVSDFTNVSLSVQLNTFKQILPTLCASSSRDCREMLGDSLILMGESGGNDYNYPFFEDKSINEIKELTPLIIKAISDAIVDLIDLGGKTFLVPGSFPVGCSAAYLTLFQTAKEKDYDPLTGCLPWLNDFGKHHDEQLKTEIRRLRKLYPHVNIMYADYYNSLYRLYQKPTKYGFKNRPLAACCGVGGQYNFTIGEECGYEGVGYCQNPSEYINWDGYHITEAAHQKMAHGILNGPYATPAFNWSCLDAASVDNESSFGS | [[21](#_ENREF_21)] |

**References**

1. Vujaklija D, Schroder W, Abramić M, Zou P, Leščić I, Franke P, Pigac J: **A novel streptomycete lipase: cloning, sequencing and high-level expression of the *Streptomyces rimosus* GDS(L)-lipase gene**. *Arch Microbiol* 2002, **178**(2):124-130.

2. Wei Y, Schottel JL, Derewenda U, Swenson L, Patkar S, Derewenda ZS: **A novel variant of the catalytic triad in the *Streptomyces scabies* esterase**. *Nat Struct Biol* 1995, **2**(3):218-223.

3. Bielen A, Četković H, Long PF, Schwab H, Abramić M, Vujaklija D: **The SGNH-hydrolase of *Streptomyces coelicolor* has (aryl)esterase and a true lipase activity**. *Biochimie* 2009, **91**(3):390-400.

4. Côté A, Shareck F: **Cloning, purification and characterization of two lipases from *Streptomyces coelicolor* A3 (2)**. *Enzyme Microb Technol* 2008, **42**(5):381-388.

5. Leščić Ašler I, Ivić N, Kovačić F, Schell S, Knorr J, Krauss U, Wilhelm S, Kojić-Prodić B, Jaeger K-E: **Probing enzyme promiscuity of SGNH hydrolases**. *ChemBioChem* 2010, **11**(15):2158-2167.

6. Kauppinen S, Christgau S, Kofod LV, Halkier T, Dorreich K, Dalboge H: **Molecular cloning and characterization of a rhamnogalacturonan acetylesterase from *Aspergillus aculeatus*. Synergism between rhamnogalacturonan degrading enzymes**. *J Biol Chem* 1995, **270**(45):27172-27178.

7. Shaw J, Chang R, Chuang K-H, Yen Y, Wang Y, Wang F-G: **Nucleotide sequence of a novel arylesterase gene from *Vibro mimicus* and characterization of the enzyme expressed in *Escherichia coli***. *Biochem J* 1994, **298**:675-680.

8. Cho H, Cronan J: ***Escherichia coli* thioesterase I, molecular cloning and sequencing of the structural gene and identification as a periplasmic enzyme**. *J Biol Chem* 1993, **268**(13):9238-9245.

9. Wilhelm S, Tommassen J, Jaeger K-E: **A novel lipolytic enzyme located in the outer membrane of *Pseudomonas aeruginosa***. *J Bacteriol* 1999, **181**(22):6977-6986.

10. Riedel K, Talker-Huiber D, Givskov M, Schwab H, Eberl L: **Identification and characterization of a GDSL esterase gene located proximal to the swr quorum-sensing system of *Serratia liquefaciens* MG1**. *Appl Environ Microbiol* 2003, **69**(7):3901-3910.

11. Wang H, Dowds B: **Phase variation in *Xenorhabdus luminescens*: cloning and sequencing of the lipase gene and analysis of its expression in primary and secondary phases of the bacterium**. *J Bacteriol* 1993, **175**(6):1665-1673.

12. Taniguchi H, Hirano H, Kubomura S, Higashi K, Mizuguchi Y: **Comparison of the nucleotide sequences of the genes for the thermostable direct hemolysin and the thermolabile hemolysin from *Vibrio parahaemolyticus***. *Microb Pathog* 1986, **1**(5):425-432.

13. Thornton J, Howard SP, Buckley JT: **Molecular cloning of a phospholipid-cholesterol acyltransferase from *Aeromonas hydrophila*. Sequence homologies with lecithin-cholesterol acyltransferase and other lipases**. *BBA-Lipids Lipid Met* 1988, **959**(2):153-159.

14. Roberts MR, Foster GD, Blundell RP, Robinson SW, Kumar A, Draper J, Scott R: **Gametophytic and sporophytic expression of an antherspecific *Arabidopsis thaliana* gene**. *Plant J* 1993, **3**(1):111-120.

15. Talker-Huiber D, Jose J, Glieder A, Pressnig M, Stubenrauch G, Schwab H: **Esterase EstE from *Xanthomonas vesicatoria* (Xv_EstE) is an outer membrane protein capable of hydrolyzing long-chain polar esters**. *Appl Microbiol Biotechnol* 2003, **61**(5-6):479-487.

16. Kram BW, Bainbridge EA, Perera MAD, Carter C: **Identification, cloning and characterization of a GDSL lipase secreted into the nectar of *Jacaranda mimosifolia***. *Plant Mol Biol* 2008, **68**(1-2):173-183.

17. Oh IS, Park AR, Bae MS, Kwon SJ, Kim YS, Lee JE, Kang NY, Lee S, Cheong H, Park OK: **Secretome analysis reveals an *Arabidopsis lipase* involved in defense against *Alternaria brassicicola***. *The Plant Cell Online* 2005, **17**(10):2832-2847.

18. Hong JK, Choi HW, Hwang IS, Kim DS, Kim NH, Choi DS, Kim YJ, Hwang BK: **Function of a novel GDSL-type pepper lipase gene, CaGLIP1, in disease susceptibility and abiotic stress tolerance**. *Planta* 2008, **227**(3):539-558.

19. Okamura Y, Kimura T, Yokouchi H, Meneses-Osorio M, Katoh M, Matsunaga T, Takeyama H: **Isolation and characterization of a GDSL esterase from the metagenome of a marine sponge-associated bacteria**. *Mar Biotechnol* 2010, **12**(4):395-402.

20. Abdelkafi S, Ogata H, Barouh N, Fouquet B, Lebrun R, Pina M, Scheirlinckx F, Villeneuve P, Carrière F: **Identification and biochemical characterization of a GDSL-motif carboxylester hydrolase from *Carica papaya* latex**. *BBA-Mol Cell Biol Lipids* 2009, **1791**(11):1048-1056.

21. Ling H, Zhao J, Zuo K, Qiu C, Yao H, Qin J, Sun X, Tang K: **Isolation and expression analysis of a GDSL-like lipase gene from *Brassica napus* L**. *J Biochem Mol Biol* 2006, **39**(3):297.

**Figure S1.** Alignment of seed sequences.

Multiple sequence alignment was generated by PROMALS. Evolutionary conservation of different motifs is shown by different colours:

| > 80 % |
| --- |
| > 60 % |
| > 40% |
| < 40% |


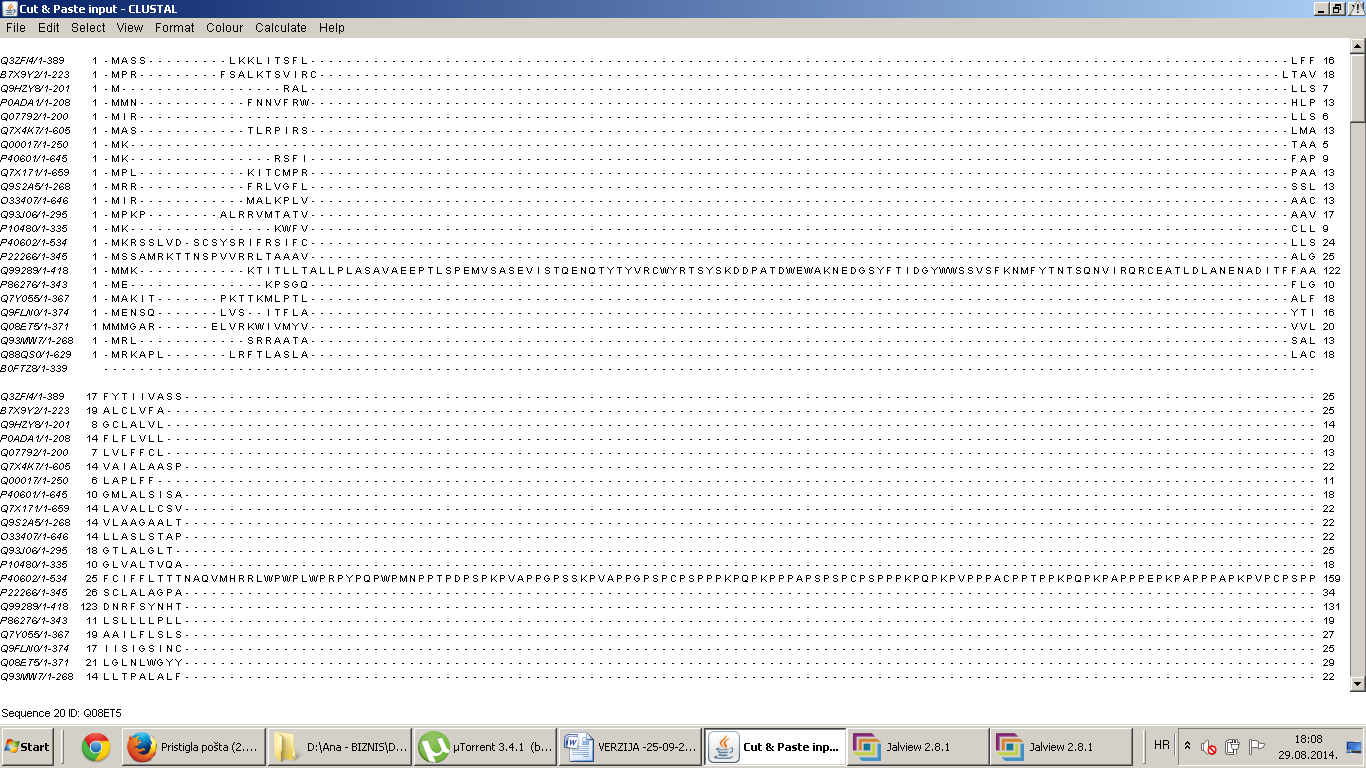


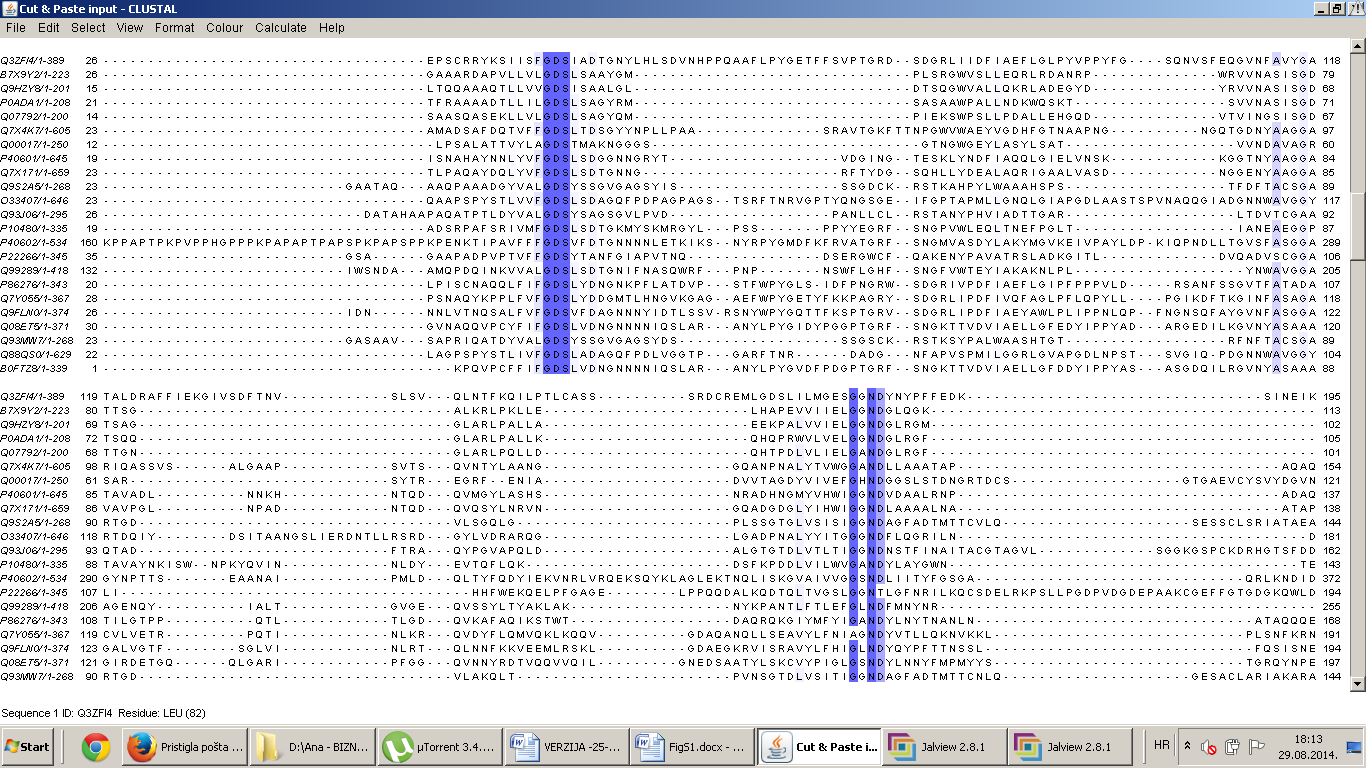


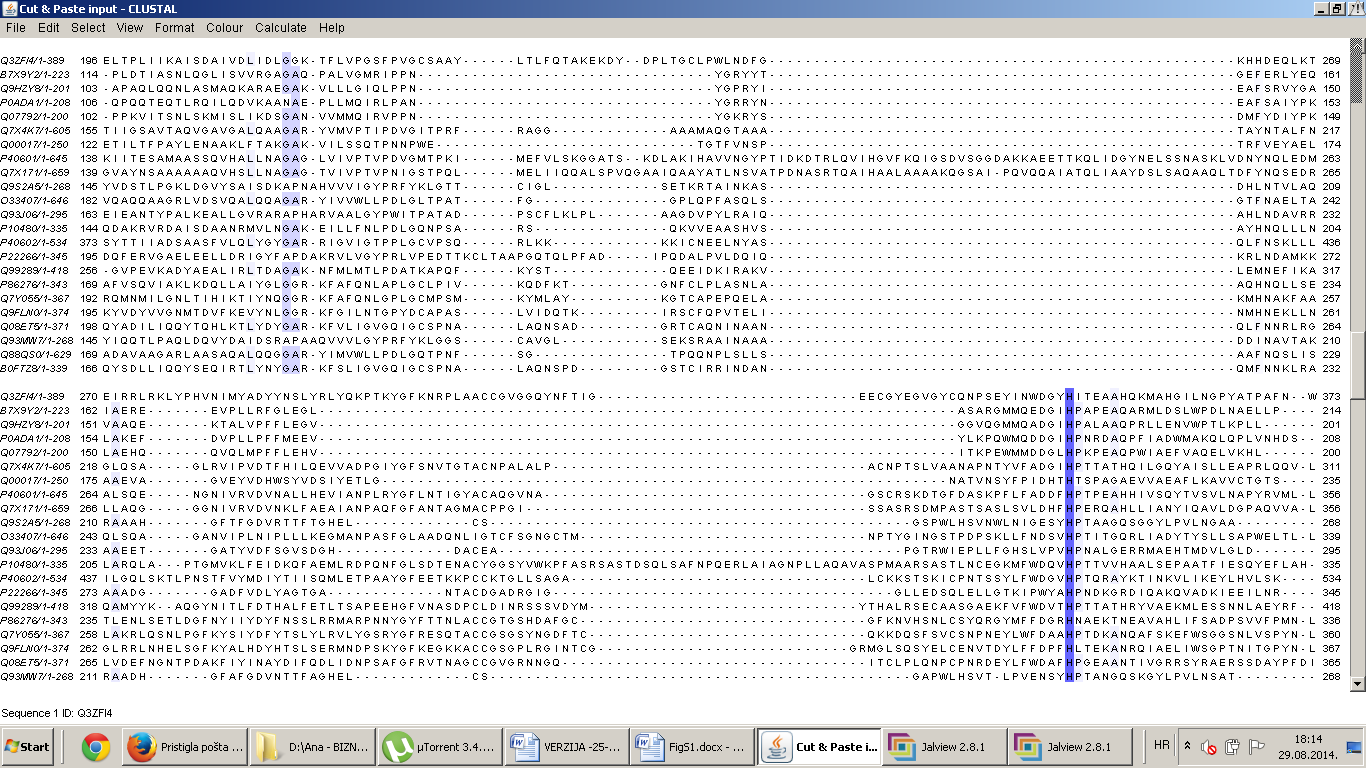


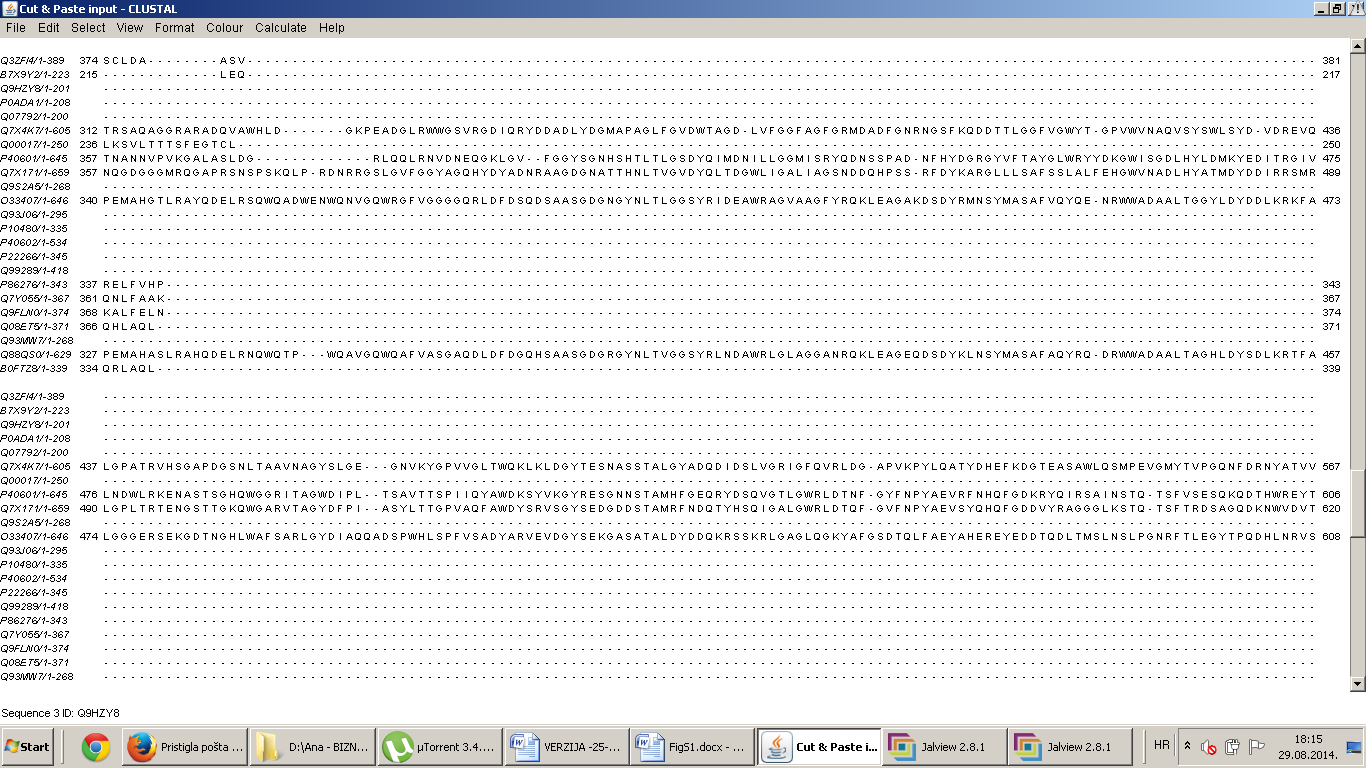


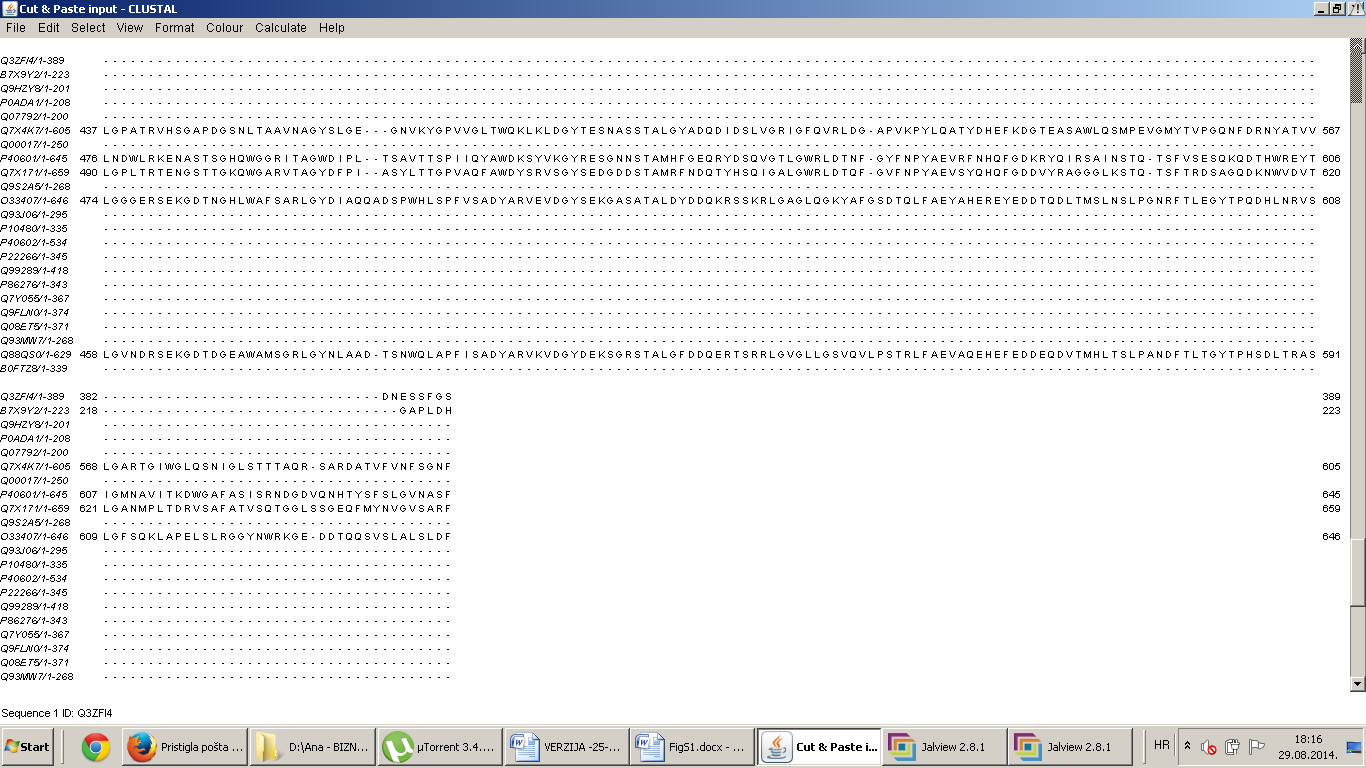


**Figure S2. Conserved Blocks derived from the seed sequences.** Blocks were obtained from MSA of seed sequences and visualized by WebLogo. Polar amino acids (G, S, T, Y and C) are green, neutral (Q and N) are purple, basic (K, R and H) are blue, acidic (D and E) are red, and hydrophobic (A, V, L, I, P, W, F and M) are black.

**
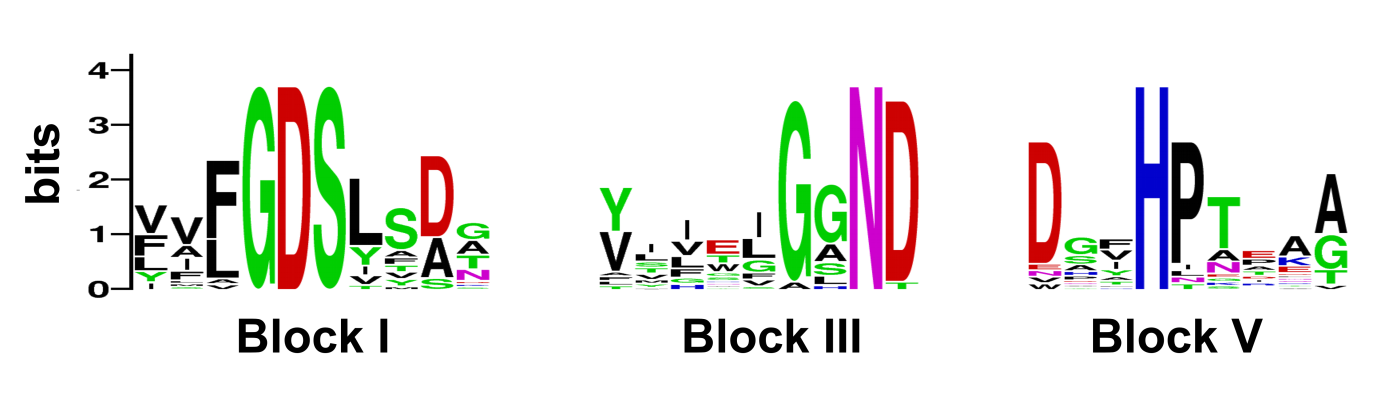
**
