# Supplementary material for: An effective approach for annotation of protein families with low sequence similarity and conserved motifs: identifying GDSL hydrolases across the plant kingdom
Source: BMC Bioinformatics. 2016 Feb 18;17:91. doi: 10.1186/s12859-016-0919-7 (PMC4757993; doi:10.1186/s12859-016-0919-7)
Supplement: Additional file 7: — Secondary structure predictions of selected GDSL hydrolases belonging to CLANS groups A-F. Ali2D program (http://toolkit.tuebingen.mpg.de/ali2d) was used for alignment-based secondary structure predictions. WebLogo was used to visualize positions of conserved Blocks I, III and V with catalytic residues. (PDF 437 kb) [file 12859_2016_919_MOESM7_ESM.pdf]

Secondary structure predictions of selected GDSL hydrolases belonging to CLANS groups A-F.

Ali2D program (http://toolkit.tuebingen.mpg.de/ali2d) was used for alignment-based secondary structure predictions. For the full names of the sequences please refer to Additional file 7. Positions of conserved Blocks I, III and V are shown as WebLogo visualisations. Amino acid residues belonging to the catalytic triad are marked with asterisk.

Confidence:
0123456789
HHHHHHHHHH – α-helices
EEEEEEEEEE – β-sheets

GROUP A

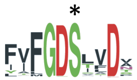

```
A1     ------------------MAKFVCLV--------IVS----IPLLLLLLGTAAKA-------------------------------QLGNSTNATAVFCFGDSTVDAGNNNYLNTYFSIARANHTPYGCDYD--NQ-AP
A2     ------------------------------------------------------------------------------------------ASSVPALFAFGDSLVDSGDNAHVG----------YPYGIDFP--GG--Q
A6     ------------------MRA--------------------LVVLAFFLGMA-------------------------------------SAQIVPALFAFGDSLVDSGNNNML---PTIARANHPPYGYNFD--NH-AA
A7     ------------------MQK--------------------LLALSLIVLLAAAE----TSHAKHRN---------GGR-------GGRGGRGAKAIFIFGDSLVDSGNNNYL---NSLAKANFAPNGEDWP--NH-LG
A8     ------------------MKAAA------------------LLLLCFILSFHA------------------------------------AEAQQVAQFIFGDSLVDSGNNDYI---LSIARANFFPNGIDTQ--NR-VA
A9     ------------------MATRASTSS-------RVSPAFTFLVIFFLLSLTASV----EAAGRGVN-------------------NDKKGGGLGASFIFGDSLVDAGNNNYL---STLSRANMKPNGIDFKASGG-TP
A40    ------------------MSRAAAMA--------AVAAA--VLVCATALGVVGA--------------------------------EVVDEFGGGASFIFGDSLVDAGNNNYI---PTLSRANMTPNGIDFAATGG-AP
A56    ------------------MAFQGCTPS------TQFQ---QWTTILLLCYLGGSF----SCGAQVIV--------NPGP-------QASSSSFPTASFIFGDSLVDAGNNNYI---GSLARANYGGNGVDFP--GG-KA
A11    ------------------MDFTYRCS---------LK---PFNCTFLLLWL-----------------------------------SHFQAAQSFTNFIFGDSLVDVGNNNYI---FTLSKADSSPYGIDFAPSNG-QP
A10    ------------------MESYLRKWC-------LVS----VWVLLLGLGF-----------------------------------KVKAEPQVPCYFIFGDSLVDNGNNNRL---RSIARADYFPYGIDFG-----GP
A12    ------------------MAR--------------------VCVMMMAMAIA-----------MAMN-------------------IAMGDPIAPCYFIFGDSLVDSGNNNRL---TSLARANYFPYGIDFQ---Y-GP
A37    ------------------MGRATMRWP-------QPA----LMLLVVAARLA----------TVAVV-------------------VVRGEPQVPCYFVFGDSLVDNGNNNDI---ASLARANYPPYGIDFA---G-GA
A38    ------------------MEVRR-------------L----LCVVAVVVSCW----------ALAAP-------------------VARCDPQVPCYFIFGDSLVDNGNNNYI---VSLARANYPPYGIDFA---G-GP
A25    ------------------MATFKL-----------FC----MLVIFLVFGVGLGQ----NVDPFEPG-------------------VGRRREMVPAMFIFGDSLIDNGNNNNL---PSFAKANYFPYGIDFN---G-GP
A26    ------------------MATFKL-----------PC----MLVIFFVLGVGLGQ----NVDPFGSQ-------------------VGRRREMVPAMFIFGDSLIDNGNNNNL---PSFAKANYFPYGIDFN---G-GP
A31    ------------------MRSFST-----------DL----VLSVTLILVLL---------ATRAC--------------------AQPQQGQVPCFFIFGDSLVDNGNNNRL---LTLSRANYRPYGIDFP---Q-GV
A36    ------------------MDDYG------------RL----ALAVAVVAAVA---------GTSGGAA------A-WGW-------QQQQPPLAPCMYVFGDSLVDNGNNNDI---LSLARANYRPYGIDFH---E-GP
A48    ------------------MKESYYCN---------SA----ICCFLFILFLV---------SGSVHG-------------------RHDRGQLVPCFFIFGASSFDNGNNNAL---PTLVKSNYPPYGIDFP---A-GP
A13    ------------------MNTNRKKMK------VHIGG--YVLILALTVSVILQQPE-----------------------------LVTGQARVPAMFVLGDSLVDAGNNNFL---QTVARANFLPYGIDMN---Y-QP
A34    ------------------MGHSR---------------------------------------------------------------GAAAASRVPALFVLGDSLVDDGNNGAL------ARADYYPYGVDFPP-LG-AA
A15    ------------------MKFCA------------------IFVLFIVLAI-------------------------------------NGYDCKIVQFIFGDSLSDVGNNKNLP--RSLATANLPFYGIDFG--NG-LP
A29    ------------------MKFEV------------------ALAIWVVVAVLGV--------------------------------TIDGGECKIVQFIFGDSLSDVGNNMYLS--RSLAQASLPWYGIDFG--NG-LP
A23    ------------------MAALAA-----------VA----VLVVAMVISGGGGV-------------------------------AAAVTSKKPVIYIFGDSMSDVGNNNYLI--LSLAKSDYPWYGVDYE--TG-FP
A28    ------------------MAILG------------------LALAAFILAAKA---------------------------------TTLASAASLVTFIFGDSLTEVGNNKYLQ--YSLARSDYPWYGIDFP--GG-RA
A19    ------------------MAKLVGAT--------LYL---YLFLFLLLLHLHCHQCKPAAAASFVVH--------GGGG-------ATARAAAVRAVFVFGSSLVDNGNNNHLN-GSGAVRADYAPYGVDFPL----GA
A27    ------------------MASQVINS--------RLL----LLTIPILACVFL-------CSCYARA-------------------TDKNGARIRGMFVFGSSLVDNGNNNFLK--NSMAKADFLPYGIDFPY----GP
A54    ---------------------------------------------------------------------------------------------VPALFIFGDSLADPGNNNHL---ISLAKSNHPPYGRQFD--TH-MA
A21    M---------------VGMKKKS------------------VGLGRLSLMISMVQ-----VLGAVGG---------GGV-------HPSKMRLVPAVYVLGDSTLDVGNNNHLP-GKDVPRANKPYYGIDFP--GS-KP
A39    ------------------MRIGV------------------LLLPCLGICMQVAL-----IGGTTVA-------------------SGGGGVRAPAMFVFGSSILDVGNNNYLQ-GATVGRANSPYNGVDFP--GS-VP
A43    ------------------M----------------------VV-------------------------------------------LHSADASIPAMFILGDSTADVGTNSLLP--FSFIRADFPFNGIDFP--SS-QP
```

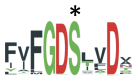

```
A44   ------------------M----------------------VV-------------------------------------------SHSADGPLPALFILGDSTADVGTNTLLP--QSVVRADLPFNGIDFP--HS-RP
A18   MHTRC-----ERRSERATMGSCTLSS---------GG----VCVVVVLLCLAACC-------------------------------LTCARGAVPAIYVLGDSQADVGNNNYLL--HSLLKANFPHNGIDYP--GG-KP
A5    ------------------M---------------------------------------VNETSSIAT-------------------PPFTSPLVPGFFVYGDSTVDVGNNNYL---QTIARANLAPYGRDFD--TH-LP
A4    ------------------MKFNIYTSA--------------FFLFYFIIRIV--------------------------------------SHNISGTLTFGDSLLDVGINNYLN-ATPTSHCNNPPYGRIFD--TG-KP
A16   ------------------MQRNRVLA---------FL----LLAAQLLVKIP------------------------------------ETCAKFPALIVFGDSTVDSGNNNQI---STVLKSNFQPYGRDYF--DG-KA
A30   ------------------MGYMHVLS--------LFC----MQIILLLVVVA------------------------------------ETTAKVPAIIVFGDSSVDAGNNNAI---STLLKSNFKPYGRDFE--GG-LP
A17   ------------------MASP-------------LV----RLLLLLLLVVAA------ASRGAASA-------------------AKAKAARVTAVIVFGDSTVDTGNNNQI---GTPLRSDFPPYGRDMP--GGARA
A22   ------------------MEHGSV-----------------KLLLLVVFCVSPWQ-----VAATTTA---------NGT-------GGGGRPRVPAVLVFGDSIVDTGNNNAV---LTLTRSNFRPYGKDLN--GG-EP
A32   ------------------MEFLSSRLTIMSYCFYSTS----VLFLTVVCTVSSLV-------------------------------KLPPNVTIPALLVFGDSIVDAGNNNDL---ETLVKSNFPPYGKDFE--GG-IP
A45   ------------------MKMLSS--S------SSTIPL-FVSVFIILCSTEALI-------------------------------KLPDNETVPALLVFGDSIVDPGNNNDL---VTFAKGNFPPYGRDFI--GG-IP
A47   MLQICFKILGSKPKER--MKMLSSSPS------SSTIPL-FVSVFIILCSTEALV-------------------------------KLPDNETVPALIVFGDSIVDPGNNNDL---VSVAKCNFPPYGRDFI--GG-IP
A33   ------------------MW---------------PV----VLVVVLILFGGPVT-----SASSAAGGGNNNATRAAGNNGGGNKPQHQKKPLAPALIVFGDSIVDPGNNNDI---RTIVKANFPPYGNDFQ--NH-RP
A41   ------------------MGFARNAL---------GA----FLLLVLILSVA------------------------------------HGDPLVPALCIFGDSVVDAGNNNNL---ATLVKANFPPYGRDFV--TH-RP
A42   ------------------MGIHCC----------WAL----FLALALALTFATIG-------------------------------EAQDATLVPAIITFGDSAVDVGNNDYL---LTIFKANYPPYGRDFI--NH-QP
A46   ------------------MSDCAFFKMAI---WGGAA----ALVLALALQFATMAD------------------------------AQGTTSIAPAMILFGDSAVDSGNNNYF---PTAFKANYLPYGKDFI--SH-QP
A51   ------------------MGFAA------------VV----VVVAVLVLGVGSCA-------QGIGA-------------------RGPQKPLVPALILFGDSTVDVGNNNFL---NTPARSNFLPYGRDFD--TR-EP
A52   ------------------MELHKLAA---------LV----LWLTCLIAWVQGFE-------------------------------AAAPAVNVPAIILFGDSTVDVGNNNFL---NTIAKSNFLPYGRDFD--TK-TP
A55   -----------------------------------MI----RSFLVLLSSVAVLGT---RRAGFFNN---------TKI-------PDNYRQYTAGVVIFGDSTVDVGNNNHL---VTVVKSNFKPYGRSFQ--GG-KS
A20   ------------------MALKPTLA--------ALL----FSLAAIHGIVVVAV----DAAAAAVS-------------------RGGSARRIPAVFAFGDSTLDAGNNNRL---VTAVRADHPPYGQDFP--GG-AP
A24   ------------------MAPSLATV---------VA----IFLAAILLSSPPPC----AAASSAPA-------------------PAAAGHGVPAVFAFGDSTLDPGNNNRL---ATLVRADHAPYGRDFP--GG-AA
A35   ------------------MAQAH------------PV----LLLLLFLSSTAVSS-------------------------------SKRIQPKFSAIFYFGDSVLDTGNNNHL---PTVAVANHVPYGRDFP--GK-KP
A49   ------------------MERAKASR---------YL----VTLFLSLIQVATA--------------------------------QTTNSPLASALYVFGDNSVDVGNNNYL---NTLFKSNHKPYGRDWH--GYSRP
A50   ------------------MDFRS------------VA----ALLLALCISIP--------ANFCGEA-------------------RLQRRIDVPAYFVFGDSFADVGTNNFLP--YAASRANFPPYGETFF---H-KA
A53   ------------------MDLKT------------AL----TLVAVVLAAIP--------VVPVDAT-------------------RAHKEFDVPAIFAFGDSLGDAGTNSFIP--QATARADFPPYGKTFF---R-KP
A3    ------------------MEAFK------------------VLVIVLVACIMISQ-------------------------------AASTNPPVQGLFVFGNSALDGGQNTYIP--GSKIVSAIPPYGKTYF---S-KP
A14   ------------------MENSQLVS---------IT----FLAYTIIISIGSIN------------------------C----IDNNNLVTNQSALFVFGDSVFDAGNNNYID-TLSSVRSNYWPYGQTTF---K-SP
```

```
A1    TGRFSNALVLPDLIAQYIGVA---RAFPFLHPSA------NGMNLT-QGVNFASGGAAIIDKLSSNLVL-------------QTPYTFSVQVE-WFRNVT-QRLQAVE----G-AT-AAAS---------------RI-
A2    ASRFCNGRLLVEYIASHLGLP---IPPAYLQ---------SGNNIL-KGANFGSAGSGILPQTVMVN---------------GGGQALGSQIN-DFQSLK-QKMVQMI----G-SS-NASD---------------VV-
A6    TGRFCDGKLIPDFLASLLGLP---FPPPYLS---------AGDNIT-QGVSFGSASSGIGRWTGQGF-----------------VLSFANQVD-GFREVQ-SRLVRRL----G-PM-RAMS---------------LI-
A7    TGRFCNGRLVADYISEYMGTE---PVLPILDPKN------TGRNLL-RGANFASAGSGILDDTGAMF---------------VQRLRVSEQYN-LFRRYK-GQLASFV----G-GR-AADR---------------IV-
A8    TGRFCNGLLISDFVSQFLGAQ---PVLPFLDPSA------RGRDLL-RGSNFASAGAGIVADTGSIF---------------LRRITMPEQIG-LFQRYQ-SQVSSLI----G-PQ-ATGR---------------LI-
A9    TGRFTNGRTIGDIVGEELGSA--NYAIPFLAPDA------KGKALL-AGVNYASGGGGIMNATGRIF---------------VNRLGMDVQVD-FFNTTR-KQFDDLL----G-KE-KAKD---------------YIA
A40   TGRFTNGRTIADIIGEMLGQA--DYSPPFLAPNT------SGGAIL-NGVNYASGGGGILNGTGKVF---------------VNRIGMDLQVD-YFNVTR-RQLDALL----G-KE-KARE---------------FLR
A56   TGRFCNGRTVADIIGQLLGIP---FAPVFLNPAA------KGKAIL-RGVNYASGGAGILDFTGYTF---------------VNRIPLWQQIS-MFRNTT-QQIMQLL----G-PE-SGAA---------------LI-
A11   TGRFTNGRTISDIVGEALGAK--SPPPPYLEPNT------EANTIR-NGINYASGAAGILDDTGLLF---------------IGRVPLREQVS-NFEKSR-EYMVRVI----G-EN-GTKE---------------ML-
A10   TGRFSNGRTTVDVLTELLGFD--NYIPAY--STV------SGQEIL-QGVNYASAAAGIREETGAQL---------------GQRITFSGQVE-NYKNTV-AQVVEIL----G-DEYTAAD---------------YL-
A12   TGRFSNGKTTVDVITELLGFD--DYITPY--SEA------RGEDIL-RGVNYASAAAGIREETGRQL---------------GARITFAGQVA-NHVNTV-SQVVNIL----G-DENEAAN---------------YL-
A37   TGRFSNGLTTVDAISRLLGFD--DYIPAY--AGA------SGDQLL-TGVNFASAAAGIRDETGQQL---------------GQRISFGGQLQ-NYQAAV-QQLVSIL----G-DEDSAAN---------------HL-
A38   SGRFTNGLTTVDVIAQLLGFD--NFIPPF--AAT------SGDQLL-GGANFASAAAGIRAETGQQL---------------GGRIPFAGQVQ-NYQTAV-QTLVSIL----G-DQDTASD---------------HL-
A25   TGRFSNGYTMVDEIAEQLGLP---LIPAY--SEA------SGDQVL-NGVNYASAAAGILDITGRNF---------------VGRIPFDQQIR-NFQNTL-DQITNNL----G-AD-DVAR---------------QV-
A26   TGRFSNGYTMVDEIAEQLGLP---LIPAY--SEA------SGDQVL-NGINYASAAAGILDVTGRNF---------------VGRIPFDEQIR-NFQNTL-DQITDTL----G-AD-DVAR---------------QV-
A31   TGRFTNGRTYVDALAQLFGFR--NYIPPY--ART------RGPALL-RGVNYASGAAGIRDETGNNL---------------GGHTSMNQQVA-NFGMTV-EQMRRYF---RG-DNNALTS---------------YL-
A36   PGRFTNGRTMVDFLSDMLRLRP-PLLPPY--ATA------RPEDLP-RGVNFASGASGILPETGNNLLIINQELDDACGNGQGGHYPLSEQVD-HFRAAV-SDMGNTSEF-RG-NATKVAA---------------HL-
A48   TGRFSNGRNIVDIISEFLGFE--DYIPSF-ASTV------GGEDIL-KGVNYASGGSGIRAETGQHN---------------GARISMDAQLR-NHHITV-SRLINRL----GQNESAAKE---------------YL-
A13   TGRFSNGLTFIDLLARLLEIP---SPPPFADPTT------SGNRIL-QGVNYASAAAGILDVSGYNY---------------GGRFSLNQQMV-NLETTL-SQLRTMM----S-PQ-NFTD---------------YL-
A34   TGRFCNGKTVADALCDLLGLQ---YVPPYTSTRA--LNGTAAMQVL-GGVNYASAAGGILDETGQHL---------------GERFSLSQQVL-NLEATL-DGAIRPL-F-GG-DHDGYER---------------HL-
A15   NGRFTNGRTVSDIIGDKIGLP---RPVAFLDPSM------NEDVILENGVNYASGGGGILNETGGYF---------------IQRFSLWKQIE-LFQGTQ-DVVVAKI----G-KK-EADK---------------FF-
```

```
A29  NGRFTNGRTVADIIDTAAIYY--NLPPAFLSPSL------TENLILENGVNYASGGGGILNETGGYF---------------IQRLSLNKQIE-LFQGTQ-QMIISKI----G-KE-KSDE---------------FF-
A23  TGRFTNGRTIGDIMAAKFGVP---PPPPFLSLYM------TDDEVL-GGVNFASGGAGLLNETGIYF---------------VQYLSFDNQIS-SFEEIK-NAMIAKI----G-KK-AAEE---------------VV-
A28  TGRFTNGRTIGDIISAKLGIP---SPPPFLSLSK------NDDALL-TGVNYASGGAGILNDTGLYF---------------IQKLSFYDQIE-CFKKTK-ESIRAKI----G-ED-AANK---------------LC-
A19  TGRFSNGRNVIDALGELLRLPAAGLLPPFADPAT------RGRAAL-HGVNFASGGSGILDLTGKNK---------------GEVLSLKQQIT-NFEAVTLPDLRAHL---QG-AT-TATTTTGHKMKGQDFFDQCYL-
A27  SGRFTNGKNVIDLLCDQLKLP---LVPAFTDPST------KGTKII-HGVNYASGASGILDDTGLLA---------------GNVISLNQQVR-NFEEVT-LPVLEAE----M-GF-QRRE---------------LL-
A54  TGRFTNGRTAVDFLAEELGLP---LVPPFLDSST------KGQKLL-QGVNYASAGSGILNSTGMFF---------------GEIITTWKQLE-YFRDSTQPEIYKLL----G-KK-AGED---------------FF-
A21  TGRFSNGFNAADYVAKNLGFD--KSPPAYLVLKA--RNYLVPAALV-MGVNYASAGAGILDSTNT-----------------GRSIPLSKQVV-YLNSTR-AEMVAKA----G-SG-AVSD---------------LL-
A39  TGRFSNGYNIADYVAKNMGFA--CSPPPYLSMVQSSSGPLVQTALT-SGINYASGGAGILDSTNA-----------------GSTIPLSKEVK-YFGATK-AKMVAAV----G-PN-TANP---------------AI-
A43  TGRFSNGFNTVDFLANLTGFQ--ISPPPFLSLVD--SQSSMNKQFL-KGVSFASGGSGLLDTTGQSL----------------GVIPLGKQIQ-QFATVQ-SNLTAAI----G-SD-ETEK---------------LL-
A44  TGRFSNGFNTADFLAKHIGYR--RSPPPFLSILS--HSSSLSKKFL-RGVNFASGGSGILDTTGQTL----------------GIITLGAQIQ-QFATVH-SNLTAAI----G-PE-ETEK---------------FL-
A18  TGRFSNGYNFVDLIAISLGVP---SPPPYLSISS---KPMNSSVYL-KGVNFASGGAGVSNLTNL-----------------AQCISFDEQIEGDYHRVH-EALGKQL----G-IP-GAKA---------------HL-
A5   TGRFSNGRLSVDYLALFLGLP---FIPPLLSRNF--------TSQM-QGVNFASAGAGILNPSGSDL---------------GQHIPMAEQVE-HIVEIQ-QRLASKI----G-ED-AANA---------------VI-
A4   SGRFSDGELISDIIAKMLGLP---FPLPYLDPTA------NGDNLK-FGISFASGGSGLLNSTSELQ----------------NVAKVNLQIS-WFREYK-DKLKIVL----GTEQ-KATQ---------------FL-
A16  TGRFSNGRIAPDFISEGLGLK--NAVPAYLDPAY------NIADFA-TGVCFASAGTGLDNATSAVL----------------SVMPLWKEVE-YYKEYQ-TRLRSYL----G-EE-KANE---------------II-
A30  TGRFCNGRIPPDFISEAFGLK--PAIPAYLDPLY------SISDFA-TGVCFASAGTGYDNATSNVL----------------NVIPLWKELE-YYKDYQ-NKLRAYV----G-DR-KANE---------------IF-
A17  TGRFGNGRLAPDFMSESLGLP--PLVPAYLDPAY------GIADFA-RGVCFASAGTGLDNATAGVL----------------SVIPLWKEVE-YYREYQ-RRLRAHA----G-AA-AARD---------------VV-
A22  TGRFSNGRIPPDFLASRLGLK--DLVPAYLGTDL------TDGDLL-TGVSFASAGSGYDPLTSTLV----------------AVLPMQEQLN-MFAEYK-EKLAGIA----G-EA-AAAR---------------IV-
A32  TGRFCNGKIPSDIIAKELGIK--DTLPAYLDPAV------LPQDLI-TGVTFASSGSGFDPLTPKLV----------------SVLSLSDQLE-HFKEYI-GKLKAII----G-EE-NTIF---------------TI-
A45  TGRFSNGKIPADFIAEELGIK--EIVPAYLDPTL------QPSDIL-TGVSFASGASGYDPLTSKIP----------------AVYSLSDQLE-MFKEYT-GKLKAMV----G-EE-RTNT---------------IL-
A47  TGRFSNGKIPSDFIAEELGIK--KLLPAYLDPAL------QPSDLL-TGVSFASGASGYDPLTPKIS----------------SVFSLSDQLE-QFKEYI-GKLTAMV----G-EQ-RTNT---------------IL-
A33  TGRFCNGRIPTDFIASRLGIK--DLLPPYLSAQP-----LDKHDLL-TGVSFASGGTGFDPLTPQLA----------------SVISLPDQLT-MFHDYL-AKVRDAAGVGDG-DA-RVSD---------------IL-
A41  TGRFCNGKLATDFTAEYLGFT--SYPPPYLSQEA------QGKNLL-QGANFASASSGYYDRTAQLY----------------RAISLTQQVE-YYKEYQ-AKVVRLV----G-KA-RAHD---------------IF-
A42  TGRFCNGKLATDITADTLGFK--TYPPAYLSPKA------SGKNLL-IGANFASAGSGYDDKTAILS----------------HAIPLSQQLE-YYKEYQ-AKLAKVA----G-SQ-KAAT---------------II-
A46  TGRFCNGKLATDITADILGFE--TYPPAYLSPQA------TGKNLL-VGANFGSAAAGYDDNTAIIN----------------HAIPLSQQLE-YYKEYR-VKLAKVA----G-SK-RAAA---------------IL-
A51  TGRFTDGRMVSDYLATWLGLP---ISLPYLHPNA------TGQNLV-HGINFASAASGYLDTTSQFL----------------HVAPARMQFR-MFEGYK-VKLANVM----G-TT-EASS---------------TI-
A52  TGRFTDGRMVSDFMASKLGLP---MSLPYLHPNA------TGQNLI-YGTNFASAASGYLDTTSVFL----------------NVIPASRQLE-MFDEYK-IKLSKVV----G-PE-KSSS---------------II-
A55  TGRFCDGKITSDRITEIIGYP---YGLPYLSPEA------HGPAIL-TGINFASSASGWYDGTARNF----------------NVKGLTDQFV-WYKNWK-AEVLSLV----G-PE-KGNF---------------II-
A20  TGRFCDGKIMSDFLVEALGVK--GLLPAYHSGSE----VLSDADAA-TGVSFASGGSGLDDRTATNA----------------GVATMASQIA-DFSE-----LVGRM----G-AG-KAGE---------------VV-
A24  TGRFTDGKLITDYIVSSLGIK--DLLPAYHSSGL------AVADAS-TGVSFASGGSGLDDLTANNA----------------LVSTFGSQLN-DFQE-----LLGHI----G-SP-KSDE---------------IA-
A35  TGRFSNGRLIPDLLNEKLQLK--EFSPPFLDTRL------SSNDMV-TGVNFASAGSGLDDQTSQLS----------------NTLPMSKQVG-LFKDYL-LRLRDIV----G-DK-EASR---------------II-
A49  TGRFSNGKLFVDYLAEYLGLP---YPPPNLSP---------GEPKI-KGVNFASAGSGVLNSTASIL----------------RVASLSGQLD-WFRKHL-KTLRAWT----G-EV-WANH---------------II-
A50  TGRFTNGRNIVDLFAQTVGLP---IAPPFLQPNS---------SFI-AGVNFASAGSSLLNST--IF---------------NNAVPLSEQVD-QYKTVR-ILLRNVL----S-PL-EAQK---------------LI-
A53  TGRFTNGRTIVDFIAQKLDLP---LTPPFLEPHA---------SFT-KGVNFASGGSGLLDSTSADD----------------FSVPMSAQVQ-QFAIAK-ATLEKQL----D-AH-RAGS---------------LI-
A3   TGRWTDGRTIADFLAQALGLP---LLPPFLEP---------GANFS-SGVNFASAGAGLLDETNAHQ----------------GVISMKQQLR-QFRNVT-NEYKKEK----G-VE-FTNQ---------------LL-
A14  TGRVSDGRLIPDFIAEYAWLP---LIPPNLQPFN------GNSQFA-YGVNFASGGAGALVGTFSGL-----------------VINLRTQLN-NFKKVE-EMLRSKL----G-DA-EGKR---------------VI-
```

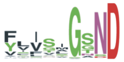

```
A1   RNAFCLISIGSNDFSYKSM-------DTTTSSLSDA--------DFRSLLVNT-LSTR--------------IQDIY-SIGCRRFIVSAIGPLGCTPITLTLMCGPYNA--TC--------------------------
A2   AKSIFYICSGNNDINNMYQRTKR------------------ILQSDEQIVINT-FINE--------------LQTLY-NLGARKFVIVGLSAVGCIPLNIV--------------------------------------
A6   SRSIFYICTANNDV-NNFVL--------RFRTELPI--------DLRDGLLVE-FALQ--------------LERLY-RLGARKFVVVNLSAVGCIPMNQRF-------------------------------------
A7   AAGLYSFTIGGNDYINNYLQPL----SARARQYTPP--------QYNTLLVST-FKQQ--------------LKDLY-NMGARKISVGNMGPVGCIPSQITQ-----RG----V-------------------------
A8   ANSLVSVTVGGNDYINNYLLPG----SARRAQLSPF--------QFNSLLVST-LRDQ--------------LQQIS-NLGARKIVVSNMGPIGCIPSQKSM-----RP----P-------------------------
A9   KKSIFSITIGANDFLNNYLFPLLS--VGTRFTQTPD--------DFIGDMLEH-LRDQ--------------LTRLY-QLDARKFVIGNVGPIGCIPYQKTI-----NQ--LD--------------------------
A40  KKAIFSITVGSNDFLNNYLMPVLS--TGTRIRQSPD--------AFVDDLIFH-LRDQ--------------LTRLH-TLDARKFVVANVGPLGCIPYQKTI-----NR--VG--------------------------
A56  RNSIYSVTMGSNDFLNNYLVVG----SPSPRLFTPK--------RFQERLINT-YRSQ--------------LTALV-NLGARKLVISNVGPLGCIPYRMAV-----SS--TT--------------------------
A11  KNAMFTITIGSNDILN-YIQPSIP--FFSQDKLPTD--------VLQDSMVLH-LTTH--------------LKRLH-QLGGRKFVVVGVGPLGCIPFARAL-----NL--IP--------------------------
A10  KRCIYSVGMGSNDYLNNYFMPQF---YSTSRQYTPE--------QYADDLISR-YRDQ--------------LNALY-NYGARKFALVGIGAIGCSPNALAQ-----GS--QD--------------------------
```

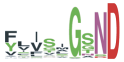

```
A12   SKCIYSIGLGSNDYLNNYFMPVY---YSTGSQYSPD--------AYANDLINR-YTEQ--------------LRIMY-NNGARKFALVGIGAIGCSPNELAQ-----NS--RD--------------------------
A37   SQCIFTVGMGSNDYLNNYFMPAV---YSTSQQYTPA--------QYADVLIDQ-YSQQ--------------VRTLY-NYGARKVALMGVGQVGCSPNELAQ-----HS--AD--------------------------
A38   SRCIFSIGMGSNDYLNNYFMPAF---YNTGSRYTPE--------QYADSLIAD-YRRY--------------LQTLY-SYGARKVVMIGVGQVGCAPNELAR-----YS--AD--------------------------
A25   GRSIFFVGMGSNDYLNNYLMPN----YPTRNQYNGR--------QYADLLTQE-YSRQ--------------LTSLY-NLGARKFVIAGLGVMGCIPSILAQ-----SP------------------------------
A26   GRSLFFVGMGSNDYLNNYLMPN----YPTRNRYNGR--------QFADLLTQE-YSRQ--------------LTKLY-NLGARKFVIAGLGVMGCIPSILAQ-----SP------------------------------
A31   SKCIFYSGMGSNDYLNNYFMSDF---YSTSHDFTSK--------AFAAVLLQD-YTRQ--------------LTQLY-ALGARKVIVTAIGQIGCIPYELAR-----YN--GT--------------------------
A36   GRCIFFVGMGSNDYLNNYFMPDY---YDTARRYSPR--------DYAALLLQG-YSDQ--------------LTQLY-GLGARKFVVAGVGLIGCIPYELAR-----MD--DDHGPSSRPSNQSAVSSQDIAISIGIGG
A48   NKCIYAAGLGTNDYVSNYFLPLL---YPTSRIYTPE--------QYALVLAQQ-YSQQ--------------LKTLYTNYGARKIALFGLAQLGCAPSVVAS-----NG-ATN--------------------------
A13   ARSLVVLVFGSNDYINNYLMPNL---YDSSIRFRPP--------DFANLLLSQ-YARQ--------------LLTLY-SLGLRKIFIPGVAPLGCIPNQRAR-----GI--SP--------------------------
A34   ARSIAVVVIGGNDYLNNYLLTPLGIGYDSGDRYRPG--------EYADLLLDQYYARQ--------------ILALH-SLGLRKFLLAGVGPLGCTPGLRAS-----AG-MGP--------------------------
A15   QDARYVVALGSNDFINNYLMPV----YSDSWKYNDQ--------TFVDYLMET-LESQ--------------LKVLH-SLGARKLMVFGLGPMGCIPLQRAL-----SL------------------------------
A29   KESQYVVALGSNDFINNYLMPV----YSDSWKYNDQ--------SFIDYLMET-LEGQ--------------LRKLH-SFGARKLMVFGLGPMGCIPLQRVL-----ST------------------------------
A23   NGAIFQVGLGSNDYINNFLRPF----MADGIVYTHE--------EFIGLLMDT-MDRQ--------------LTRLY-DLGARNVWFSGLAPLGCIPSQRVL-----SD------------------------------
A28   NEAMYFIGLGSNDYVNNYLQPF----LADGQQYTPD--------EFVELLIST-LDKQ--------------LSMLY-QLGARKVVFHGLGPLGCIPSQRVK-----SK------------------------------
A19   PKSLFIIGTGGNDYLLNYFNAGS-----GPTRAPLS--------EFTSSLLTK-LSNH--------------LQRLY-DLGARKFVLFSIQPLGCTPVVRTF-----LN--AT--------------------------
A27   PKYLFVVGTGGNDYSFNYFLRQ------SNANVSLE--------AFTANLTRK-LSGQ--------------LQKLY-SLGGRKFALMAVNPIGCSPMVMAN--------RRT--------------------------
A54   RKSIFYLISGSNDFVNGYYFLI----PTTPHGISIQ--------DLMQLLIST-VSSQ--------------LKVLY-DLGVRKVGVAGLAPLGCCPSQITK-----YN--LT--------------------------
A21   AKSFFLFGVGSNDMFAFAAAQQK-----LNRSATPSE-----VEAFYTSLISN-YSAA--------------ITELY-GMGARKFGIINVGPVGCVPSVRVA-----NA------------------------------
A39   SQSIFLIGMGNNDLYVFAASERA-----RNRSAADDERSDAAAAALYAGLISN-YSAA--------------VTELY-TLGARKFAVINVWPLGCVPGQRVL-----SP------------------------------
A43   SKSLFLISTGGNDILGHFPLNGG-----LTKEDNKI--------ELELFFIEC-HSKYCPRNLILELKLLLIWQNLF-ELGARKFAIVGVPPIGCCPLSRLA-----DI------------------------------
A44   SKSLFVISTGSNDIINYFQSN--------NRTLPKE--------EFIQNLGYA-YENH--------------LRTLF-DLGARKFGILSVPPIGCCPSLRTL-----DP------------------------------
A18   AKSLFVVAIGGNDIINDLLLSPV-----SELLRSRD--------EIVSNLENT-LKRQ--------------LQTLY-DLGMRRLFFVGIAPLGCCPLIREL-----NP------------------------------
A5    SNSIHYISIGSNDFIHYYLRNV----SDVQNKMTNF--------EFNQLLISS-LVGH--------------IEDMY-ARGIRKVVTIGLGPLGCVPFFLYT-----FN--QT--------------------------
A4    NDALYFIGEGSNDYAFKSLNL-------AESLTSIE--------EFRNKLISN-YKTY--------------IEDIY-SIGGRKFVIYGLTPIGCSPGLITV-----HN--PL--------------------------
A16   SESLYLISIGTNDFLENYYLLPR-----KLRKYSVN--------EYQYFLIGI-AADF--------------VTDIY-RLGARKMSLSGLSPFGCLPLERTT-----QL--FY--------------------------
A30   SEALYLMSLGTNDFLENYYTIPT-----RRSQFTVR--------QYEDFLVGL-ARNF--------------ITELY-HLGGRKISLSGVPPMGCLPLERTT-----NI--MG--------------------------
A17   RGALHVVSIGTNDFLENYYMLAT----GRFARYSVG--------EYEDYLVAA-ARAF--------------LAAIH-RLGARRVTFAGLSPMGCLPLERTAGA-L-LG--GG--------------------------
A22   SESLFLVCAGSDDIANNYYLAPV-----RPLQFDIS--------SYVDFLANL-ASDF--------------VKQLH-RQGARRIAVLGMPPIGCVPSQRRSVA-VDAA--GG--------------------------
A32   RNSLFLVVAGSDDIANTYFTLRA-----RKLQYDVP--------AYTDLMANS-ASSF--------------AQELY-ELGARRIVVFSAPPVGCVPSQRTL-----AG--GA--------------------------
A45   SKSLFLVVQSSNDIASTYFTV-------RRVQYDFS--------SYADLLVTW-ASSF--------------FKELY-GLGARRIAVFGAPPLGCLPSQKSI-----AG--GI--------------------------
A47   SKSLFLVVQSSNDIATTYFDI-------RKVQYDFA--------SYADLLVTW-ASSF--------------FKELY-GLGARRIAVFSAPPLGCLPSQRSL-----AA--GI--------------------------
A33   SRGVFAICAGSDDVANTYFTMR------ARSNYDHA--------SYADLLVHH-ATAF--------------VENLI-RAGARRVAFIGIPPIGCVPSQRTM-----SG--GL--------------------------
A41   SGGIHLLSAGSSDFVQNYYINPL-----LNRAYSAD--------QFSDLLMKS-YTTF--------------VQNLY-GLGVRKIGVTTLPPTGCLPAAITL-----FS--SG--------------------------
A42   KDALYVVGAGSSDFIQNYYVNPF-----LNKVYTPD--------QYASILVGI-FSSF--------------IKDLY-GLGARRIGLTSLPPLGCLPATKTL-----FG--FH--------------------------
A46   KGALYLVGFGTADFLQNYYVNPS-----LKKLYTPD--------QYSTYLATI-FSSF--------------IKDLY-GLGARKIGVVPLPPLGCFPETITM-----FR--YR--------------------------
A51   TNALYVVSSGSNDFILNYFISPE-----MQNRYSTT--------QFSSLVMSD-QKEF--------------VQNLY-KAGARKMAILGFPAIGCIPAQITL-----FG-GLE--------------------------
A52   SQALYFVSSGSNDFILNYFVNPA-----LQSSYSPT--------EFNAALMST-QTEF--------------VQKLY-QAGARKIGIFGFPPIGCIPAQITL-----FGIDVN--------------------------
A55   STSLYIFSTGANDWVNNYYLNPV-----LMKKYNTD--------EYITFLIGL-ARGY--------------IQELY-DLGGRNIAVLGLPPLGCLPSQITL-----HG--KG--------------------------
A20   NKSLFLVSAGTNDMIMNYYLLP--------SKYTLD--------QYHALLIGK-LRSY--------------IQSLY-NLGARRLLVAGLPPVGCLPVQMTLAA-LRQP--PR--------------------------
A24   GKSLYVISAGTNDVTMYYLLPFR-----ATNFPTVD--------QYGDYLIGL-LQSN--------------LNSLY-KMGARKMMVAGLPPLGCLPVQKSL-----RG--AG--------------------------
A35   ASSLIFISSGTNDFSHYYRSS-------KKRKMDIG--------DYQDIVLQM-VQVH--------------VKELY-DLGGRQFCLAGLPPFGCTPIQITL-----SR--DP--------------------------
A49   GDAVYVISTGSNDYSNNYLLDPS-----QHDNVDED--------TFVELIYNE-MVSF--------------VHELL-AVGARKIMVVSFTPSHCDPSQLLTQL-P-DP--ST--------------------------
A50   SKSVFLILSGSDDLLE-YLSNFE-----IQNRMNAT--------QFMSNVVEA-YRTT--------------LTDLY-KGGARKALLVGLTPLGCSPSARAT-----NP--RN--------------------------
A53   SKSIFLFISGSNDLSA-FLRDAQ-----LQQQVNAT--------QFVASLIDV-YQKS--------------LLAVY-HAGARKAIVVGVGPLGCSPLARAS-----NT--AN--------------------------
A3    RNSVALFSMGANDIANAVPSS-----------------------FLFQEMIQA-YSSA--------------IQEIY-NYGIKHIIILLAPPIGCTPNLRAV-----SA--QSR-------------------------
A14   SRAVYLFHIGLNDYQYPFTTNSS-----LFQSISNE--------KYVDYVVGN-MTDV--------------FKEVY-NLGGRKFGILNTGPYDCAPASLVI-----DQ--TK--------------------------
```

```
A1    ------------------------------------------------RSMCNETTNGIVYAFDVAVENML-RNLS-------ASLSG----FRYYYNYDAFNITRDAIRNPATYG----YTIVDRGCCGS--GTTEI-
A2    ------------------------------------------------GGQCASIAQQGAQTYNNLLQSAL-QNLR-------NSLKD----AQFVMT-NFYGLMVDVHNNPQSYG----FTDSSSACCPQ--GSH---
A6    -------------------------------------------------GRCGSAGMNAALSFNLGLASVL-DSLR-------ISMRG----ARIVTA-NMEGLMLQVKSNPRAYG----FSNTVQGCCPL----NQP-
A7    ------------------------------------------------NGQCVQNLNEYARDYNSKLKPML-DELN-------RELRG----ALFVYV-NAYDILSDLVSNPGKNG----FTVSNSACCGQ--GNYNG-
A8    ------------------------------------------------SGLCLPDLQQYAQHFNSLLRPML-SQLT-------QQNPG----SVFLYS-NGYDMLMDIMANGGSYG----LSNVRDACCGQ--GAFNG-
A9    ------------------------------------------------ENECVDLANKLANQYNVRLKSLL-EELN-------KKLPG----AMFVHA-NVYDLVMELITNYDKYG----FKSATKACCGNG-GQYAG-
A40   ------------------------------------------------EDECVKLPNQLAAQYNARLRELI-VELN-------GNLPG----ARFCLA-NVYDLVMELITNYPNYG----FETASVACCGNG-GSYDG-
A56   ------------------------------------------------KGQCVQSDNSLVMSFNSALKSLV-DELN-------GKYPN----AKFILA-NSFNVVSQIISNPGGFG----FATKDQACCGVPIGFHRG-
A11   ------------------------------------------------AGKCSEQVNQVVRGYNMKLIHSL-KTLN-------NELRSEDYNTTFVYA-NSYDLFLKLVLNYQLFG----LKNADKPCCGG----YFP-
A10   ------------------------------------------------GTTCVERINSANRIFNNRLISMV-QQLN-------NAHSD----ASFTYI-NAYGAFQDIIANPSAYG----FTNTNTACCGI--GRNGG-
A12   ------------------------------------------------GVTCDERINSANRIFNSKLVSLV-DHFN-------QNTPG----AKFTYI-NAYGIFQDMVANPSRYG----FRVTNAGCCGV--GRNNG-
A37   ------------------------------------------------GATCVPEINGAIDIFNRKLVALV-DQFN--------ALPG----AHFTYI-NVYGIFEDILRAPGSHG----LTVTNRGCCGV--GRNNG-
A38   ------------------------------------------------GVTCVDRIDDAIQMFNRRLVGLV-DEFN--------ALPG----AHFTFI-NAYNIFDDILANAASYG----FTVTNAGCCGV--GRNNG-
A25   ------------------------------------------------AGICSDSVNQLVQPFNENVKAML-SNFNA------NQLPG----AKSIFI-DVARMFREILTNSPAYG----FSVINRGCCGI--GRNRG-
A26   ------------------------------------------------AGNCSDSVNKLVQPFNENVKAML-KNFNA------NQLPG----AKFIFI-DVAHMFREILTNSPAYG----FSVINRGCCGI--GRNRG-
A31   ------------------------------------------------NSRCNEKINNAISLFNSGLLKLV-QNFNN------GRLPG----AKFVYL-DSYKSSNDLSLNGTSF---------DKGCCGV--GKNNG-
A36   GGGGGGIGIGGGRVGGRLPSTMPLPYTDGDGGNGNGNGNGNTNPAPNNGGGCNETINSAIDIYNRGLLAMV-KRFNS-----RGGLRG----AKFVFL-DAVQSGKDLVANAAAHG----FTVLDRGCCGV--GRNNG-
A48   ------------------------------------------------GSACVDYINDAVQLFNNRLKELV-GELN-------RNLTD----AKFIYV-NVYEIASEATSYPS-------FRVIDAPCCPV--ASNNT-
A13   ------------------------------------------------PDRCVDSVNQILGTFNQGLKSLV-DQLN-------QRSPG----AIYVYG-NTYSAIGDILNNPAAYG----FSVVDRACCGI--GRNQG-
A34   ------------------------------------------------QGQCVEQVNQMVGLFNQGLRSLV-DQLNA------DHHPV----ATFVYG-NTYAAVQDMINNHSKYG----FTVVDSGCCGV--AQIVT-
A15   ------------------------------------------------DGNCQNKASNLAKRFNKAATTML-LDLE-------TKLPN----ASYRFG-EAYDLVNDVITNPKKYG----FDNSDSPCCSF--YRIRP-
A29   ------------------------------------------------TGKCQEKTNKLAIAFNRASSKLL-DNLS-------TKLVN----ASFKFG-EAYDVVNDVISNPTKYG----FDNADSPCCSF--GQIRP-
A23   ------------------------------------------------DGGCLDDVNAYAVQFNAAARNLL-ERLN-------AKLPG----ASMSLA-DCYSVVMELIEHPQKYG----FKTSHTSCCDV--DTTVG-
A28   ------------------------------------------------TGRCLKRVNEYVLEFNSRVKKLI-ATLN-------RRFPN----AKLTFA-DAYGDVLDLIDNPTAYGNNFCLKISNTSCCNV--DTTIG-
A19   ------------------------------------------------SDACIEPMNHAALLFNSGLRSIV-KNHNGGV---RSHMPA----ASFVYV-NSYKIISDIIQHPAKYG----IRKTSRACCEV--SR----
A27   ------------------------------------------------RNGCIEGLNKAAHLFNAHLKSLV-DVSK-------EQMPG----SNVIFV-NSYKMIRDIIKNPVSRG----FKDTNSACCEV--MSLNEG
A54   ------------------------------------------------AGNCVEFLNDVSEKYNDALKNML-LQLR-------EELED----FHLVYS-NLYDPLMEAINNPAMYG----FNFTHAACCGV--GKLNG-
A21   ------------------------------------------------TGGCNDGMNQLAAGFDAALRGHM-SGLA-------ARLPG----LAYSIA-DSYALTQLTFADPGAAG----YANADSACCGG--GRLGA-
A39   ------------------------------------------------TGACSDTLNEVAAGFNAALGSLL-VDLA-------ARLPG----LVYSLG-DAFGFTEDVLADPAASG----YTDVAGTCCGG--GRLGA-
A43   ------------------------------------------------NDHCHKEMNEYARDFQTILSALL-QKLS-------SEYGG----MKYSLG-NAYEMTMNVIDDPPAFN----LKDVKSACCGG--GRLNA-
A44   ------------------------------------------------SYGCLEEMNEYATFFYTTIQALM-QRLS-------SEYQG----MKYSLG-NAYDMAMYVVNNPVAFN----FTDVKSACCGG--GKLNA-
A18   ------------------------------------------------TKECDAQANYMATRLNDAAVVLL-RDMS-------ETHPD----FTYSFF-DTYTAVLQSIRYPEAHG----YKEVKAACCGL--GDNNA-
A5    ------------------------------------------------GAGCVDSINFMIAEFNNALRVTA-QSLA-------MKHRN----LRIIYC-DVFQSLMPIVRTPLQYG----FVTSRSACCGA--GRFGG-
A4    ------------------------------------------------TRNCVDFLNNQAQEFNAYLVQLL-NNIT-------KELPG----SQFIYL-DKYAIFMDIIQNKFKYG----FQVINRGCCGT--GLIEF-
A16   ------------------------------------------------GSKCIEEYNIVARDFNIKMEEKV-FQLN-------RDLNG----IQLVFS-NPYDLVSEIIYHPEAFG----FENVRSACCGT--GYYEM-
A30   ------------------------------------------------HHDCLQEYNDVAMEFNGKLECLA-SQLK-------RELPG----LRLLYTRTAYDTFDQIIRTPAAYG----FQVTRRACCAT--GTFEM-
A17   ------------------------------------------------GGGCVEEYNRVAREYNGKVEAMV-RSLR-------AELPR----LKVAFI-PVYDNMLDLITHPEKYG----LENVEEGCCAT--GRFEM-
A22   ------------------------------------------------GRECDAAQNRAARLFNAKLEQEI-GCLR-------ETLQL----QSIGYV-DIYGVLDDMIADPGKYG----FDVSTRGCCGT--GEFEV-
A32   ------------------------------------------------ERECAENFNEAAKLFNSKLSKKL-DSLA-------SSLPN----SRLVYI-DVYNLLLDIIQKPQKYG----FQVADKGCCGT--GNLEV-
A45   ------------------------------------------------ERECVENYNEACKLFNTKLSSGL-DSLN-------TNFPL----AKFVYI-DIYNPLLDIIQNPQKSG----FEVANKGCCGT--GLIEV-
A47   ------------------------------------------------ERECVEKYNEASKLFNTKLSSGL-DSLN-------TNFPL----AKFVYV-DIYNPLLDIIQNPQKSG----FEVVNKGCCGT--GLIEV-
A33   ------------------------------------------------DRGCSQGHNEVAVAYNAGMVQQL-AALR-------AKYPG----TRLVFM-DIYGFLYDMMMHPQSYG----FTQSTRGCCGT--GLLEV-
A41   ------------------------------------------------SNQCVARLNQDAINFNSKLNITS-QVLQ-------NKLPG----LKLVVF-DIYQPLLNLITKPTDNG----FFESRKACCGT--GTIET-
A42   ------------------------------------------------QSGCVSRLNTDAQGFNKKINSAV-SSLQ-------KQLSG----LKIAVF-DIYKPLYDIIKSPSDYG----FAEASRGCCGT--GTIET-
A46   ------------------------------------------------KHGCIARINKNAQGFNNKINTTA-ISLQ-------KKLPA----LKIVVF-DIFKPLHDVFTSPSDYG----FAEARKGCCQT--RKTGT-
A51   ------------------------------------------------QEKCVETQNAVALEYNKVLQDEV-PKWQ-------ASLPG----SQFLYL-DAYSLLYEIFYNPAKYG----FTSTRRACCGH--GLIST-
A52   ------------------------------------------------QKTCVEEQNAIASAYNSDLAAAI-PKWQ-------SNLSG----SLLLYL-DAYSMLYDIFNNPTKYG----YTEARRACCGE--GLLST-
A55   ------------------------------------------------NQGCVEDYNAVSRKFNDQLKNVINNELK-------PKFSG----GRLIYI-DIYTTLYAIRTNSSAYG----ITEVRTGCCGT--GVIET-
A20   ------------------------------------------------PQGCIAEQNAEAEKYNAKLRKML-TKFQ-------STSPG----AKAVYA-DIYTPLTDMVDHPQKYG----FAETGKGCCGT--GLLEM-
A24   ------------------------------------------------SGGCVTEQNEAAERYNAALQKAL-SKLE-------ADSPG----AKIAYV-DIYTPLKDMAENPKKYG----FTQASLGCCGT--GMMEM-
```

```
A35   ------------------------------------------------DRACVDEQNWDAQVYNSKFQKLL-TTLQ-------GSLHG----SRIVYL-DAYRALMEILEYPAKHG----FTETTRGCCGT--GLREV-
A49   ------------------------------------------------DLFCQDPYNMLPRRFSEALRYRIQSSLTNGMLPRRSSLSGAP--TYVVYG-NIYDTIFAFVTN-----------SSNSPCCGP--TSEEDD
A50   ------------------------------------------------PGECLVEGNELAMRFNNDVRQLV-DELH-------VTFPD----YNVIFG-ESYNLIEAMINDKKSSG----LDNVNAACCGA--GFLNA-
A53   ------------------------------------------------PGECVEVANQLALGFNAALKQMV-DGLR-------AALPG----FNLVLA-NTFDTVSAMITDGKAFG----LDNVTAACCGA--GFLNA-
A3    ----------------------------------------NTNLT---PEGCIGIINTFVDSYNTKLLNLA-VKLH-------NDYRD----LNIATL-NPSPIILNVLRNPQKYG----FKEAEKACCGG--GPFNA-
A14   ------------------------------------------------IRSCFQPVTELINMHNEKLLNGL-RRLN-------HELSG----FKYALH-DYHTSLSERMNDPSKYG----FKEGKKACCGS--GPLRG-
```

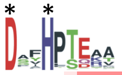

```
A1    ----GDGCQ--------------SYFG-LCFDRSKYIFFDAIHPGGKLISLLANRLSTS----------------------------------LSS--------------------------------------------
A2    ----TLNCR--------------PGAT-ICGDRTKYAFWDGIHQTDAFNSMAAQRWWTGG---------------------------------TSG---DVSPISISELA------------------------------
A6    ----WRWCF--------------DGGE-FCEKPSNFMFWDMVHPSQAFNSIAAHRWWNG----------------------------------TLE---DVYPVNIRTLASI----------------------------
A7    ----LFICT--------------AFST-ICNDRTKYVFWDPYHPTEKANILIAQQTLFG----------------------------------GTN---VISPMNLRQLLALP---------------------------
A8    ----NAICT--------------GAST-LCADRSSFLWWDPYHPTEAVNKIITDRLLDG----------------------------------PPS---DISPMNLRQVLRL----------------------------
A9    ----IIPCG--------------PTSS-LCEERDKYVFWDPYHPSEAANVIIAKQLLYG----------------------------------DVK---VISPVNLSKLRDM----------------------------
A40   ----LVPCG--------------PTTS-LCDDRDKHVFWDPYHPSEAANVLLAKYIVDG----------------------------------DTK---YISPINLRKLYSL----------------------------
A56   ----LSPCF--------------PGVP-FCRNRKSYFFWDPYHPTDAANVIIGNRFFSG----------------------------------SPS---DAYPMNIKQLAALQ---------------------------
A11   ----PFACFKG--------PNQNSSQA-ACEDRSKFVFWDAYHPTEAANLIVAKALLDG----------------------------------DQT---VATPFNIRYLNDL----------------------------
A10   ----QLTCL--------------PGEP-PCLNRDEYVFWDAFHPSAAANTAIAKRSYNAQ---------------------------------RSS---DVYPIDISQLAQL----------------------------
A12   ----QITCL--------------PGQA-PCLNRDEYVFWDAFHPGEAANVVIGSRSFQRE---------------------------------SAS---DAHPYDIQQLARLYWFKWRILIRGSLCIRVRFLNNNNYILS
A37   ----QVTCL--------------PFQT-PCANRNEYLFWDAFHPTEAANVLVGRRAYSAA---------------------------------QPS---DVHPVDLRTLAQL----------------------------
A38   ----QVTCL--------------PYQA-PCANRDQHIFWDAFHPSEAANIIVGRRSYRAE---------------------------------SPN---DVYPMDISTLASI----------------------------
A25   ----QITCL--------------PFQT-PCPNREQYVFWDAFHPTEAVNVLMGRKAFNG----------------------------------DLS---MVYPMNIEQLANLD---------------------------
A26   ----QITCL--------------PFQT-PCPNREQYVFWDAFHPTEAVNVLMGRKAFNG----------------------------------DLS---KVYPMNIEQLANLE---------------------------
A31   ----QITCL--------------PLQQ-ICQDRSKYLYWDAFHPTEVANILLAKVTYNSQ-----------------------------------T---YTYPMSIQQLTML----------------------------
A36   ----QITCL--------------PLQR-PCDDRSKYMFWDAFHPTEAVHRIYAAKAFSSN---------------------------------STA---EVYPINVSQLAAI----------------------------
A48   ----LILCT--------------INQT-PCPNRDEYLYWDALHLSEATNMFIANRSYNAQ---------------------------------SPT---HTCPIDISDLAKL----------------------------
A13   ----QITCL--------------PLQT-PCPNRNQYVFWDAFHPTQTANSILARRAFYG----------------------------------PPS---DAYPVNVQQMTLLH---------------------------
A34   ----CGLCV--------------PFVA-PCGERERYVFWDAYHPTQAANLVLAQMAFAG----------------------------------TPE---HVYPLNLRQLAEL----------------------------
A15   ----ALTCI--------------PAST-LCKDRSKYVFWDEYHPTDKANELVANILIKRFDFMRADDGI----------------------SHAPSPAPDISPSSDNN--------------------------------
A29   ----ALTCL--------------PAST-LCEDRSKYVFWDEYHPSDSANELIANELIKKFGFTRVDGTN----------------------APPPS---APSPVTAPSPVIAP---------------------------
A23   -----GLCL--------------PTAQ-LCDDRTAFVFWDAYHTSDAANQVIADRLYADMVSAGAVQGNGNATTAAAASTPAPRVVVGGGASPST---HAAP-------------------------------------
A28   -----GLCL--------------PNSK-LCSNRKDYVFWDAFHPSDAANAILAEKLFSTLFS-------------------------------GPP---SVAPT------------------------------------
A19   ---GGVLCQ--------------KGGA-ICSDRTKYAFFDGLHPTDVVNARLARKAYGSN---------------------------------SPD---KVYPINVKKLAML----------------------------
A27   G--NGILCK--------------KEGQ-ACEDRNIHVFFDGLHPTEAVNIQIATKAYNSN---------------------------------LTS---EVYPINVKQLSML----------------------------
A54   ----KFICI--------------PYSR-PCDDPQHHIFFDYYHPTSRMYDLIFRKVYFNG---------------------------------PP---------------------------------------------
A21   ----EGPCQ--------------RGAA-LCGDRDRFVFWDSVHPSQQANKLGAKAYFHG----------------------------------PPQ---FTSPINFNQLANYNS--------------------------
A39   ----EAWCS--------------RNST-LCVNRDQHVFWDRVHPSQRTAFLIARALYDG----------------------------------PSK---YTTPINFMQLAKSN---------------------------
A43   ----LLPCLK-------------PLAT-VCSNRDDYLFWDLVHPTQHVSKLAAQTLYSG----------------------------------PPR---LVSPINFSQLVEDN---------------------------
A44   ----QSPCV--------------PTAA-LCSDRDKYLFWDLFHPTKHACKLAAFTLYTG----------------------------------EPV---FVSPINFSQLAMDN---------------------------
A18   ----MFLCS--------------PASV-YCDNRTSYMFWDVVHPTQAAVEKLMKIAFDG----------------------------------SAP---LVSPKNIKQLTES----------------------------
A5    ----WMMCM--------------FPQM-ACSNASSYLWWDEFHPTDKANFLLARDIWSGN---------------------------------------VCEPGGLQDLAKAS---------------------------
A4    ----GQLCN--------------PLVG-ACDDGSLYVYFDAAHGSLATYNITATKLRAQL---------------------------------------------ESEFGVMF---------------------------
A16   ----SYLCD--------------KMNPFTCSDASKYVFWDSFHPTEKTNAIVANHVLKYD---------------------------------------------LSRFQ------------------------------
A30   ----SYLCN--------------EHSI-TCRDANKYVFWDSFHPTEKTNQIISQKLIPIL---------------------------------------------LAEFQ------------------------------
A17   ----GFMCN--------------DESPLTCDDASKYLFWDAFHPTEKVNRIMAQHTLDVC---------------------------------------------YQQGV------------------------------
A22   ----TLLCN--------------QLTATTCADDRKFVFWDSFHPTERAYSIMVDYLYQRY---------------------------------------------VDKLL------------------------------
A32   ----AVLCN--------------QHTSETCADVSDYVFWDSYHPTEKAYKALVYPLLGKY---------------------------------------------LTKFF------------------------------
```

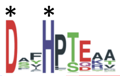

```
A45   ----ALLCN--------------RLNPFTCNDVTKYVFWDSYHPTERVYKILIGRIIQEY--------------------------------------------VGSFL------------------------------
A47   ----AVLCN--------------QFNPFTCNDVTKYVFWDSYHPTERLYKILIGEIIQEY--------------------------------------------VDSFF------------------------------
A33   ----SVLCN--------------AVTSAVCQDVGDYLFWDSYHPTEKAYKVLADFVFDNY--------------------------------------------VKLILV-----------------------------
A41   ----SLLCN--------------ARSVGTCSNASQYVFWDGFHPSESANQLLAGSLLEQG--------------------------------------------INLIGHHVLW-V-----------------------
A42   ---TSLLCN--------------PKSIGTCPNATQYVFWDSVHPSQAANQVLADALILQG--------------------------------------------IGLIG------------------------------
A46   ---VPILCD--------------PKSPGTCRNASQYVFWDDVHLSQATNQMLAESMLLQG--------------------------------------------ISLI-------------------------------
A51   ----AEFCN--------------EATSGTCSDASKFVFFDSLHPTQSVYKRLADEYIAKF--------------------------------------------ISFFKLDGGY-------------------------
A52   ----AGFCN--------------KDSVGTCTDASKYVFFDSLHPTSSVYRLVAEAYHEKV--------------------------------------------ISYLL------------------------------
A55   ----AIACN--------------QASIGTCEDANSYLWWDSFHPTEHAYNILADDLFNQA--------------------------------EAT---LRGPA------------------------------------
A20   ----GPLCT--------------DLMP-TCTTPAQFMFWDSVHPTQATYKAVADHFLRTN--------------------------------------------MLQFDD-----------------------------
A24   ----GALCT--------------SALP-QCQSPSQYMFFDSVHPTQATYKALADEIVKSH--------------------------------------------VPQLMQ-----------------------------
A35   ----ALFCN--------------ALTP-ICKNVSSYVFYDAVHPTERVYMLVNDYIVKYV--------------------------------------------IPQF-------------------------------
A49   DFPGFLNCN--------------EFTATACSNADDYVYWDSLHYTQRVQEYLANRLWNGT--------------------------------FGV---DCHPFCLQELAALP---------------------------
A50   ----QVRCGLP-----MPSGMLDVGQP-LCKHPSKFLFWDVVHPTEQVVRLLFKSFWAG---------------------------------NSS---TSYPMNIKALVSL----------------------------
A53   ----QVQCGKP-----VPPSLPGAVQD-FCRRPFKSLFWDVLHPTEHVVRILFNMLFTG---------------------------------DAT---AAYPINLRALAQL----------------------------
A3    ----AEFCGDADKHDWKPDHKN-KYTKFVCNNPKDYLYFDSNHFTEAGYWFVMKNFWHG---------------------------------SYN---IARPSNLNFFFQGFNIP------------------------
A14   ----INTCGGR-----MGLS---QSYE-LCENVTDYLFFDPFHLTEKANRQIAELIWSG---------------------------------PTN---ITGPYNLKALFELN---------------------------


A1    ----------------------
A2    ----------------------
A6    ----------------------
A7    ----------------------
A8    ----------------------
A9    ----------------------
A40   ----------------------
A56   ------------------LPG-
A11   ----------------------
A10   ----------------------
A12   LAKKIVGPGLDGNGC-CVPYKI
A37   ----------------------
A38   ----------------------
A25   ------------------IESN
A26   ------------------MESN
A31   ----------------------
A36   ----------------------
A48   ----------------------
A13   ----------------------
A34   ----------------------
A15   ----------------------
A29   ------------------SPVG
A23   ------------------PPKP
A28   -------------------PSH
A19   ----------------------
A27   ----------------------
A54   ----------------------
A21   ----------------------
A39   ----------------------
A43   ----------------------
```

```
A44    ----------------------
A18    ----------------------
A5     ----------------------
A4     ----------------------
A16    ----------------------
A30    ----------------------
A17    -----------------L----
A22    ----------------------
A32    ----------------------
A45    ----------------------
A47    ----------------------
A33    ----------------------
A41    ------------NACFNL----
A42    ----------------------
A46    ----------------------
A51    ----------------------
A52    ----------------------
A55    ------------------HP--
A20    ----------------------
A24    ----------------------
A35    ----------------------
A49    ----------------------
A50    ----------------------
A53    ----------------------
A3     ----------------NPPPTP
A14    ----------------------
```

GROUP B

```
B1     ------------------------------------------------------MA-------------------------------------MVIV-------------SVIAALISLEF---TD-------------
B4     ------------------------------------------------------MS--------------------------------------------------------LFLLLAVIL---CA-------------
B2     -------------------------------------------------------------------------------------------------------------------------------------------
B3     -------------------------------------------------------------------------------------------------------------------------------------------
B35    ------------------------------------------------------M-------------------------------------------------------VELALLLSLML---VM-------------
B34    MPYGCWAATDISSISKEQAQLSICKASCKREFKQYNNPEKAAKRPEQGQKKRSTMS---------------------SAP-----------ERQFLQ-------------SLRSAISDLLY---VAHEQFRAQYAGAIN
B8     ------------------------------------------------------MA-------------------------------------SDINRR-----------RSFSLLVLIIV---ML-------------
B24    ------------------------------------------------------MS---------------------PPPF-----STHRH-AMQIGCR--------CTMRTVWPLLLVAA---AL-------------
B10    ------------------------------------------------------MN---------------------ITK------------------------------MKLFYVILFFI---SS-------------
B16    ------------------------------------------------------MA------------------------------------------------------FIFSFLPTVII------------------
B29    ------------------------------------------------------MA---------------------------------------LK-------------ILIPWIPTLIL---LH-------------
B17    ------------------------------------------------------MG---------------------FAS-------INVS-VITLG-------------YFLMLFVTLTS---IL-------------
B26    ------------------------------------------------------ME---------------------SPS-------TIRL-ATSLC-------------CICVLLSFTAT---VI-------------
B27    ------------------------------------------------------MSFIAEPALHLLNYYCFTDMNKPSPS-------TTRL-ATSLC-------------CICVLLSFTTT---VI-------------
B28    ------------------------------------------------------MA---------------------AA--------------AVIG-------------VVLVALLSVFY---VN-------------
B30    ------------------------------------------------------MN---------------------EKG------------VGSIW-------------PAVIFVVAVSL---CF-------------
B33    ----------------------------------------------------------------------------------------------------------------------MLS---CC-------------
```

```
B6   ------------------------------------------------------MA---------------------SLD------------SHVLM-------------KLGSLFLSTLF---VS-------------
B7   ------------------------------------------------------MA---------------------S----------------SLE-------------KLISSFLLVLYSTTII-------------
B15  ------------------------------------------------------MA---------------------FAG----------------------------DARIVVVAVAAFV---LV-------------
B11  ----------------------------------------------------------------------------------------------------------------MKLLRVVLV---LL-------------
B13  DIYN--------------------------------------------------FSS---------------HRHR-RAP------------YSTYH-------------TTMKLLRVVLV---LL-------------
B12  --------------------------------------------------------------------------------------------------------------SLFSSLSSLHA---AG-------------
B21  --------------------------------------------------------------------------------------------------------------MLRAVVFIVFL---LS-------------
B25  ------------------------------------------------------MR---------------------SGR-----------------------------RMAAALVAVVWS---SW-------------
B22  ------------------------------------------------------MR---------------------VGPGSCLLLPTTAT-TMAVAHAPQRRRGVLLLVLVAALVAALPA---TC-------------
B23  ------------------------------------------------------MPG---------------D----GGA-----------------------------TGRATWVCAVLA---AF-------------
B14  ------------------------------------------------------MA--------------------------------------LLQ-------------RLLVALLRVLV---AG-------------
B9   ------------------------------------------------------MS-------------------------------------SSIS-------------PLLTTAISVAI---LL-------------
B18  ---------------------------------------------------------------------------------------------MVFS-------------RVLIVTCSLLV---LV-------------
B19  ------------------------------------------------------MA---------------------SKN-------------MAFF-------------QVLV--SSIFL---LV-------------
B20  ------------------------------------------------------MA---------------------IRF-------------LALF-------------QVLIIYSTFLH---II-------------
B31  MVFNCGSGTD--------------------KYREYDS-----------------MD---------------------ARR--------------------------VAGCAKLHLSLVLLI---LV-------------
B5   ----------------------------------------------------------------------------------------------------------------------MFQ---VN-------------
B32  ------------------------------------------------------MG--------------------------------------SLHLL-----------RFGALLLCTLH---LV-------------
```

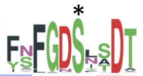

```
B1   -----------------AGSSS----------------------------------------------SWELGGFTEKI----------------------------PAIFQFGDSLSDTGNSLIAFPQAY----KRLN
B4   --------------------------------------------------------------------IQFHLGVLCDH----------------------------RVVFQFGDSLSDAGNSLLAFPGLN--GSGILG
B2   -----------------------------------------------------------------------------------------------------------DGIIIFGDSLSDTGASANVFPSNP------GN
B3   ------------------------------------------------------------------------------------------------------------AVFWFGDSFADTGNAQAASP-----FISAAE
B35  -------P------------------------------------------------------------IASAAGNGFKC----------------------------PKAFWTFGDSLSDTGNSQTTFPSAS-----RL
B34  SITDVDPPSAETKISRLSCTQSAVDAARAVGSLVSENELCRHRLGNYARSGNNVGKFGQSNFILKPDAVDVISTLKNSYSHRQILKYAFEEGGSIYRWIVAAVDGCRKAILAFGGSSTDTGEAQSFTGERELDFVTASQ
B8   --------------------------------------------------------------------YGHKGDSKCDF----------------------------EAIFNFGDSNSDTGGFWAAFPAQ---------
B24  -----------------CCASP----------------------------------------------AASAGTGRCKF----------------------------PAVFNFGDSNSDTGGFWAAFPAQ---------
B10  --------------------------------------------------------------------LQISNSIDFNY----------------------------PSAFNFGDSNSDTGDLVAGLGIR--------L
B16  -------------------------------------------------------------------------PLKFSY----------------------------PAVFNFGDSNSDTGGLVAGLAFP--------V
B29  --------------------------------------------------------------------LPVISPSNFTY----------------------------PAVFNFGDSNSDTGGLAAGVAFP--------V
B17  --------------------------------------------------------------------NPIFASRICEF----------------------------PAIFNLGDSNSDTGTLSAAFTAL---------
B26  --------------------------------------------------------------------NPVVALENCKF----------------------------PAIFNFADSNSDTGGYAAAFSQP---------
B27  --------------------------------------------------------------------NPVVALENCKF----------------------------PAIFNLGASSSDTGGYAAAFSQP---------
B28  --------------------------------------------------------------------WRLDRSSGCHF----------------------------PAIYNFGDSNSDTGSVSAVLRRV---------
B30  --------------------------------------------------------------------ASNVEGGCSRS----------------------------PVIFNMGDSNSDTGSVLNGFGFV--------R
B33  --------------------------------------------------------------------WAALAKEEC-----------------------------PQAIFAFGASMSDTGNSEAAFPYQS----VAQS
B6   --------------------------------------------------------------------IVSSESQCRNF----------------------------ESIISFGDSIADTGNLLGLSDHNN---LPMSA
B7   --------------------------------------------------------------------VASSESRCRRF----------------------------KSIISFGDSIADTGNYLHLSDVNH---LPQSA
B15  --------------------GVAVEGKGE---------------------------------------GGGGGGVGVCF----------------------------ERIFSFGDSLTDTGNFLLSVPED---FPDPAR
B11  ----------------LPAVSSC---------------------------------------------LPCRRRDDYDY----------------------------DSIFSFGDSFADTGNGAVVFAEHS--LFSPAT
B13  ----------------LPAVSSC---------------------------------------------LPCRRRDDYDY----------------------------DSIFSFGDSFADTGNGAVVFAEHS--LFSPAT
B12  ------------------------------------------------------------------AGAGASSLSVRRY----------------------------DAIFSFGDSFADTGNNPVVFGWYS--VFDPVT
B21  --------------------------------------------------------------------VTRRYGCSQSY----------------------------NAIYSFGDSIADTGNLCTGSGGCP--SWLTTG
B25  --------------------------------------------------------------------ALALAAAAQSY----------------------------NAVFNFGDSITDTGNLCT--NGRP--SSITFT
B22  --------------------------------------------------------------------AAARSKSKKSY----------------------------TAIFSFGDSLSDAGNLIV--NGTP--KALTTA
B23  --------------------------------------------------------------------PFLAAAAAGRY----------------------------HAVFNFGDSLVDAGNLVT--EGIP--DYLATA
```

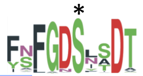

```
B14   ----------------EHAAGDAMPSPAS-------------------------------------GGGGAAVNVEARY----------------------------ARVFCFGNSLTDTGNNPLLPATAG----GPST
B9    ----------------FSTIST----------------------------------------------AATIPNIHRPF----------------------------NKIYAFGDSFTDTGNSRSGEGPAG---FGHLS
B18   ----------------LSNSSS----------------------------------------------CDATKHKNCGF----------------------------DAIYNFGTSMSDTGNAMHLTPNAS------EF
B19   ----------------FPRCSCE-------------------------------------------AYDDVAKLKQCRF----------------------------NAIYNFGASLSDTGNQIIEIPQVW------ST
B20   ----------------LPQNSCN-------------------------------------------ASNIVPKLKQCGF----------------------------DAIYNLGTSISDTGNSAIDNPSIW------QA
B31   ----------------FGQSATA--------------------------------------------------------------------------------FVCPDYIYVFGDSLTDVGNAHAELP-----IFNTVT
B5    --------------------------------------------------------------------------ARQNF----------------------PSVLCPTAIFSLTDSLSDTGNRNLEALASG--NTSPSG
B32   --------------------------------------------------------------------SAQTALPNCSY----------------------------PAVYGFGDSLTDVGNGIAAFPEK----FQHCE

B1    TSPYGET--FFHGPS--GRECDGRLIVDFLASSYGL-PLLEPYL-----RRFKGQDWRHGVSFAACGASALGRSFFHD----HNI--SIGATFQLDIQLQW-FREFKTVSAMRS-------SKRGRRTHPSADDFSQ-A
B4    LPPYGET--FFKRAT--GRVTDGRLIIDFLASGMGV-PFLDPYL-----DKAS-ANFVYGANFATVGATALSIRDFYRK---RNIM-PRRPTFSFDTQLQW-FHSFQEQALMN---------GSSAYSVPNLRQFRE-A
B2    QSPYGIT--YPGSPT--GRFSDGRLIIDYISAGLKF-KYPEPYF-----VTIN-PDYRTGINFAQAGSTALNTVF--------------QNPIYFSYQLQQ-FLQFKQRLESD----------AYRKSLPPLKFYQT-F
B3    YLPYGMT--HFGKPS--NRYSDGRLVTDFFAQAFRHKSSPGPIL-----QSLN-SNYEHGIVFAVSGATALNTSY--------------VVPFYLPVQLGFIFPSLPDRKTKL-----------------PRKLRS-V
B35   YPPYSTSFTFRDKPG-FNRFSDGRLIVDFISLAFGH-PYYGTYA-----HALNGANYVRGANFAYAGATANATTF--------------VTPIHLNLQVDN-FLNFKSKALDTG--F----YFPDRPYQPVWNAFSD-G
B34   FLPYGIT--YFGHPA--DRYSDGRLIIDFLSQAFGL-RLLDPYF-----DNIA-PDFRQGINFATGGANVRRVES------------IDVVPIYLGLQVNQ-AIRFYHKSLDV----------PSGALVPAPSSFGNLG
B8    SGPWGMT--YFKKPA--GRASDGRLIIDFLAKSLGM-PFLSPYL-----QSIG-SDFRHGANFATLASTVLLPNTSLFV--------SGISPFSLAIQLNQ-MKQFKVNVDES----HSL-DRPGLKILPSKIVFGK-S
B24   QGPFGMT--YFGRPA--GRASDGRLVIDFIAQAMGL-PLLSPYL-----QSIG-SDYRHGANSATLASTVLLPNTSVFV--------TGISPFSLGIQLNQ-MKEFRNRVLSS----NGN-----NGQLPRPDIFGK-A
B10   DLPNGQN--SFKTSS--QRFCDGRLVIDFLMDEMDL-PFLNPYL-----DSLGLPNFKKGCNFAAAGSTILPANP------------TSVSPFSFDLQISQ-FIRFKSRAIEL----LSKTGRKYEKYLPPIDYYSK-G
B16   GPPNGQT--YFQQPH--GRFCDGRLIIDFLMDAMDR-QFLNPYL-----DSVGAPNFQKGCNFATGGSTILPANA------------ASTCPFSFGVQVAQ-FVRFKDRVLQL----LAE-DKEFQKYLPLEDYFMQ-G
B29   GAPNGET--YFNKPS--GRFCDGRLIIDFLMDSMDL-PYLNAYL-----DSIGAPSFRTGCNFATGGATILPANA------------ASLSPFSFGFQVAQ-FIRFKARVLEL----LGK-DKKLQKILPLEDYFRD-G
B17   NSPYGDT--YFHMPA--GRFSDGRLIIDFIAKSFNL-PYLSAYL-----NSLG-ASYTNGANFASARATIRFPSPIIPAS-------GGYSPFYLDVQYQQ-FMQFKDRSQII----RKQ-GGKFAKLMPKEDYFRK-A
B26   PWPYGRT--FFRMPA--GRFSDGRLMIDFIANSFGL-PFLSAYL-----NSLG-SNYTNGANFATAAATIRLPTRIIPA--------GGFSPFYLGLQYDQ-FVQFKSRTLKI----RKR-GGVYKDLMPKEEYFPK-A
B27   PWPYGRT--FFRMPA--GRFSDGRLMIDFIANSFGL-PFLSAYL-----NSLG-SNYTNGANFATAASTIRLPTSIIPA--------GGFSPFYLDLQYDQ-FVQFKSRTLKI----RKR-GGVYKDLMPKEEYFPK-A
B28   PFPNGQN---FGKPS--GRYSDGRLIIDFIAENLGL-PYLNAYL-----DSIG-TSFRHGANFAATGSTIQPPHLRMFE--------EVCYPLSLNIQLLQ-FAQFKARTTQLY--PQVQ-NSDIKNTLPRPEDFSK-A
B30   PPPFGR---LFHRYV--GRVSDGRLIIDFLCENLTT-SYLTPYL-----KSMG-SSFTNGANFAVGGGKTFPR----------------FDFFNLGLQSVQ-FFWFQNQSIEL--------TSKGYKDFVKEEDFKR-A
B33   NPPYGNT--FFGRPA--NRFSDGRVVLDFFAQALKI-PLLSPYL-----QSVG-YDFSHGANFAFAGVTTQNITYPA----------TVTAPFYYWVQTKQ-FQLFKERTLAL----------SYVKLLTKPKHFQT-A
B6    FPPYGET--FFHHPT--GRFSDGRLIIDFIAEFLGL-PYVPPYF-----GSTN-GNFEKGVNFAVASATALESSFLEE----KGY--HCPHNFSLGVQLKI-FKQSLPNLCGL--------------PSDCRDMIGN-A
B7    FLPYGES--FFHPPS--GRYSDGRLIIDFIAEFLGL-PYVPSYF-----GSQN-VSFDQGINFAVYGATALDRVFLVG----KGIE-SDFTNVSLSVQLNI-FKQILPNLCTS--------------SSHCREMLGD-S
B15   SLPYGQT--FFGRPS--GRYSDGRNLLDFFAEAFGL-PFVPPYL-------AG-GDFRQGANFAVGGATALNGSFFRD----RGVE-PTWTPHSLDEQMQW-FKKLLTTVSSS--------------ESELNDIMTK-S
B11   KPPYGMT--FFGQPT--GRNSNGRLIIDFIAEKLGL-PFVPPYL------AHN-GSFRQGANFAVAGATSLDASFFSD---IPGVG-KFVLNTSSSVQLGW-FDSLKPLLCSP--------------AQECKGFFHK-S
B13   KPPYGMT--FFGQPT--GRNSNGRLIIDFIAEKLGL-PFVPPYL------AHN-GSFRQGANFAVAGATSLDASFFSD---IPGVG-KFVLNTSSSVQLGW-FDSLKPLLCSP--------------AQECKGFFHK-S
B12   RPPYGST--FFGHPT--GRNCDGRLVVDFVAERLGV-PLLPPFL------AYN-GSFRRGANFAVGAATALDSSIFHAGDPPPGAS-PFPVNTSLGVQLGW-FESLKPSLCST--------------TQECKDFFGR-S
B21   QPPYGNT--HFGHPT--GRCTDGRVIVDFLADHFGL-PLLPPSK------AIGAGDVKKGANMAIIGATTMDFEFFQK----HGLGNSIWNNGPLGTQIQW-FQQLMPSICGT--------------GAECQSYFNN-S
B25   QPPYGET--YFGTPT--CRCCDGRVIPDFLSSKFGL-PFLPPSK------STT-ADFKKGANMAITGATAMDAPFFRS----LGLSDKIWNNGPISFQLQW-FQQISSAVCGN---------------DCKSYLGN-S
B22   RPPYGMT--FFRKPT--GRCSNGRLVVDFLAEHFGL-PLPQPSQ------AKG-KDFKKGANFAITGATALEYSFFKA----HGIDQRIWNTGSINTQIGW-LQDMKPSLCKS--------------DQDCKDYFSK-S
B23   RPPYGQT--YFGYPT--GRCSDGRLVVDFIAQEFGL-PLLPPSK------AKN-ASFAQGANFAITGATALDTDFFQK----RGLGKTVWNSGSLFTQIQW-LRDLKPSLCSS--------------AQECKEFFAK-C
B14   SPPYGMT--FFHRPT--GRSSDGRLLIDFIVKALRA-PQPTPYLA----GKTA-ADLLAGTNFAVGGATALEPAVLAR----MGI--VSAVPVSLSNETRW-FQDALQLLASS--------------INARRRIAET-S
B9    SPPYGMT--FFRRPT--NRYSDGRLTIDFVAESMNL-PFLPPYLSLKTTNANGTATDTHGVNFAVSGSTVIKHAFFVK----NNLS-LDMTPQSIETELAW-FEKYLETLGTN---------------QKVSLFKD-S
B18   NAPYGRS---IKDAK--GRYSDGFLVIDYFAKAACL-PLLNPYL-----NKDV-KDTHGGVNFAVAGATALPREALEK----FNL--QPFINISLDIQLQW-WGNYAKSLCNN-------------SKVDCKEKLKS-S
B19   KLPYGQA---IHKVT--GRSSDGLLIIDYIAKSAGL-PFLEPYLKY---QNAT-SFLSHGVNFAVGGSTVLSTKFLAE----KNIS-NDHVKSPLHVQLEW-LDKYLQGYCHD--------------AKDCQEKLAS-S
B20   MFPYGKT---INEAT--GRPSDGLLIIDYIARSADL-PLVVPYKNS---SALH-LSTSRGVNFAYSGAPALSEEALAK----KNIT-LDWAKPTLSVQLGW-LDDYFKGYCNN-------------VKGDCKEAVSS-S
B31   NYNYGMSYSFPDRPCERTRFSDGRLLIDYTAQAFGV-PFLQPYS-----RHLHSSAYKHGVNFAYSGGTAKFTPI------P-------FPTFFLEREVEN-YFKFRASYSGP-----------------FVNVST-A
B5    SFPYGMT---IGKPT--GRYSDGYLLIDFLTRGLKLGDSARPSL------TYN-GTYFTSLNFGYAGATVCPSNN------------NFSTPHILSAQVSD-FLWHKQQVKDY----------QDGAKVDKNVLYEK-A
B32   EDPYGVT--FPMHAA--DRFTDGKMFIDFLAFGVRR-RPTYAVL-----RGTA-GDFTYGTNFAASGGPARPVKVWNSDD-------KFTTPFSLEVQQQW-FQRYKIRLWFYESPVYNP-NGRLVQSLPKLANISA-S
```

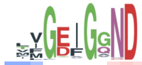

```
B1     LYIVGEIGGNDYGDMMS--TMDYSQM-L--QFVPMVVQTIRDFIQ--------------ARMNFPNPF--------NLY--------------NLGARKFLVTNIPRQGCNPSFLVSRRP-----SDRLD-ELGCIADF
B4     LYVIGEIGGNDYAMLHG-SGVDFLDIIK--FFVPRVVHEIEETIR-------------------------------ELY--------------QAGARNFLVINVPIQGCNVRSLATADW----SKEEMD-ELGCLARF
B2     LYAV-EIGINDIINNIIYNNKSLSYIAN--ITIPQAVAAIKSSLQ-------------------------------LLY--------------NEGGRKFLVFTITPLGCTPQFKTIFAS---PNPTAYD-SYQCLIAF
B3     LHVV-VVGTNDIFGAYIRKLMDPGNVTV--VIVPQVVQAISHAIQ-------------------------------TLS--------------DSGASQILVLNSFPHGCMPLILSVFG------DLPKD-SRGCLSPL
B35    AYYIPEIGGIDLIVATSVLNLPSPVVIA--SFVPAAVAAVKTAIT-------------------------------TLH--------------DSGARLFFIGNTPPQGCNPAQLTQFF------NRTKD-ALLCVDDI
B34    LYFI-FAGVNDICFATM-TNSGVERI-RD-VILPEIVSNVSLAITVVAGEFKTDKNLSRTGKTFHDNFAFFFPCLQRLYN-------------NSTTRQFLVLGISPFGCTAFALGLGLPDLNPAYGPIG-QDGCAQGI
B8     LYTF-YIGQNDFTSNLA--SIGVERV-K--LYLPQVIGQIAGTIK-------------------------------EIY--------------GIGGRTFLVLNLAPVGCYPAILTGYTH----TDADLD-KYGCLIPV
B24    LYTI-DIGQNDFTSNLG--SLGVESV-K--RSLPSIVNQISWTIQ-------------------------------DMY--------------NIGARHFMVFNMAPIGCYPAFLTELPH----NSNDLD-EFGCMKSY
B10    LYMI-DIGQNDIAGAFY--SKTLDQV-L--ASIPSILETFEAGLK-------------------------------RLY--------------EEGGRNIWIHNTGPLGCLAQNIAKFGT----DSTKLD-EFGCVSSH
B16    LYMF-DTGQNDIDGAFY--SKSEDQV-I--ASFPTILSEFEAGIK-------------------------------RLY--------------TAGARNFWVHNTGPLGCLPRIIATFGK----NPSKLD-QPVCVDSH
B29    LYGF-DVGQNDLDGAFY--SKSEDQV-A--AFIPTILSEFEAGVE-------------------------------RLY--------------NEGARNLWIHGMGPLGCLARIIATFGK----DASKLD-QFGCVNSH
B17    LYTF-DIGHNDLGAGFF-SNMSIEEV-K--ATVPDIVNRFSIYVK-------------------------------NIY--------------EVGGRSFWIHSTGPIGCLAYILTGFP------SAEKD-SAGCSKQH
B26    LYTL-DIGQNDLGEGFF-ANMSIQEV-N--ATVPDIINGFSTNVR-------------------------------RIY--------------KSGARSFWIHNTGPIGCLPYILANFQ------AAQRD-SAGCSKPH
B27    LYTL-DIGQNDLGEGFF-ANKSIQEV-N--ATVPDIINGFSTNVR-------------------------------RIY--------------KSGARSFWIHNTGPIGCLAYILANFQ------AAQRD-SAGCSKPH
B28    LYTM-DTGQNDLHDGFT--SMTVEQV-Q--KSIPNIINQFSQAIE-------------------------------QLY--------------QQGAKIFWIHNTGPIGCLPFFVINYPP----KPDNVD-QTGCIKSY
B30    LYMV-DIGQNDLALAFG--NSSYAQV-V--ERIPTFMAEIEYAIV-------------------------------SLY--------------QHGGRKFWVHNTGPLGCLPQQLVNISR----SSDDFD-NHGCLKSR
B33    LYFT-TFGANDFIVPLFRLGLSIQQV-Q--SNVSIISNAMVQNTE-------------------------------ELY--------------NQGARTLMVFNVPPLGCYPAFLASPRI---RNMSTVD-PHGCLATV
B6     LILMGEIGANDYNFPFF-QLRPLDEV-K--ELVPLVISTISSAIT-------------------------------ELI--------------GMGGRTFLVPGGFPLGCSVAFLTLHQT---SNMEEYDPLTGCLKWL
B7     LILMGEIGVNDYNYPFF-EGKSINEI-K--QLVPLVIKAISSAIV-------------------------------DLI--------------DLGGKTFLVPGNFPLGCYPAYLTLFQT---AAEEDHDPFTGCIPRL
B15    LFLVGEVGGNDYNHLIV-RGKSLDEL-H--ELVPKVVGTITSAIT-------------------------------ELI--------------NLGAKKLVVPGNFPIGCVPLYLSIFPS---QKEDYYDEKTGCIKWL
B11    LFFMGEFGVNDYSFSVF--GKTPLEV-R--SMVPDVVKTISSATE-------------------------------RIIK-------------RDGAKAVVVPGIPPLGCMPPNLAMFPS---TDPAGYEPGTGCLRQF
B13    LFFMGEFGVNDYSFSVF--GKTPLEV-R--SMVPDVVKTISSATE-------------------------------RIIK-------------RDGAKAVVVPGIPPLGCMPPNLAMFPS---TDPAGYEPGTGCLRQF
B12    LFFVGEFGFNDYEFFFR--KKSMEEI-R--SFVPYIIETISIAIE-------------------------------RLI--------------KHGAKSLVVPGMTPSGCTPLILAMFADQ--AGPDDYDPVTGCLKVQ
B21    LFVVGEFGGNDYNAPLF-GGTAMAEV-R--SYVPEIVDRIASGVE-------------------------------TLI--------------ELGAVDVVVPGVLPIGCFPLYLTLYQS---SSKDDYD-EIGCLKSF
B25    LFVFGEFGGNDYNAMLF-GNYNADQA-S--TYTPQIVSTIANGVE-------------------------------KLI--------------AMGATDIVVPGVLPIGCFPIYLTIYGT---SNSGDYD-SLGCLKKF
B22    LFVVGEFGGNDYNAPLF-SGVKFSEI-K--TYVPLVTKAIANGVE-------------------------------KLI--------------ELGATDLLVPGVLPIGCFPLYLTLYNT---SSKSDYNARTGCLRRY
B23    LFIVGEFGGNDYNAPLF-AGKDLKEA-Y--KLMPHVIQGISDGVE-------------------------------QLV--------------TEGAKDLIVPGVMPSGCFPVYLTMYTD---PKEGHGS-RTGCLKRF
B14    LFFFGEIGVNDYFLALA-SNHTVEQAAA--TLVPDIVGVIRSAVI-------------------------------DAI--------------VAGARTVVITGMIPLGCEPQLLALFPA---GSAADYDPDTGCNARF
B9     LFWIGEIGVNDYAYTLG-STVSSDTI-R-----ELSISTFTRFLE-------------------------------TLL--------------NKGVKYMLVQGHPATGCLTLAMSLAA------EDDRD-SLGCVQSA
B18    LFSIEAMGANDYLTAML-RGKTIEEL-KKMDLVSQVIKANEEGVR-------------------------------KII--------------GYGATQVLVTGYLHVGCAPSLLAMRSN----SSDARD-QFGCLKDY
B19    LFTT-FAGGNDYGTAFS-QNKTLEEVKN--SLVPACVETLKHVVK-------------------------------KFI--------------HHGARRVLVHGLPPSGCAPLFLTKFSS---NNSAAYD-GFGCLKSY
B20    LFMI-NFGTNDYGYAFS-QNHNIEEI-KKNGLVSDVVEAIKQALQ-------------------------------KII--------------SQGARKVLVFGVALDGCRPISVTMESA---NKSATYD-RFGCVKDN
B31    LHMIPEIGANDYIYAFT-LGLSPAEANA--KLDGLILRAIERTVE-------------------------------KLH--------------AGGARFFYIFNLPPVGCTPFMLTLFSH-----RSPKD-QFGCLSAH
B5     LYFI-EIGGNDINYMMP----RFSDILN--TTIPSVISGIKSSIL-------------------------------SLY--------------ESGARNFLVLNLPRSDCAPGYMSAFTEFADIFNTHTD-QFGCIVEV
B32    LYTV-WAGYQDYFFSLYDKKLTVGQT-L--KIVPDVVKAIEEHIE-------------------------------KMLAVVEYTPPGFPSMLMPPAKEILIQNQLPLGCVPAMLTLYGG----SKAKYD-EYGCLSSL
```

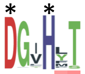

```
B1     NALNAHHNSLLREAVDDLRVSL--AGASIAHADFYSAIEPILRN-PQSY--------------------------GF-TEPRTVCCGTPW-----LT----QVVDCVDGGMINGI--LTKGQTCADPSVHIYWNGVHFT
B4     NEVGYRHKFLLERMVRKLRDEL--PGSAFATGDFLGITKKIFEN-YKHY--------------------------GF-THRFEACCGI-----YNAT----TTVDCGESVFVNGA--RIQGPTCDDPSQYIFWNDNHFT
B2     NNISQYFNSKLVDAVVSLRNQY--TDAKFYIADMYNPYYKILQN-SSAY--------------------------GF-TNIRDACCGTGA--PYNYS----PFQICGT----------PGVSSCLNPSTYISWDGLHYT
B3     NEVAEAFNRSLYKLVQDLSSKL--KNTLLLYADAFKFTLDVMDR-PTDF--------------------------GTNETKTSACCGTGG--AYNFN----STKLCGKDF--------QPESTTLKPSEFVSWDGIHFT
B35    NAINRAYGAALQQALEDLRTSLGGDGTQIFLMDNYNASIEIFTN-PATY--------------------------GF-TNTQQACCGSGG--PYNYN----SAFTCGN---IGSC--CQGQSACATPGSYVSWDGIHYT
```

```
B34   NGFVKELNELLLVELESLRSQL--SETTIVYADTYSIIYDAVIN-PSLY----------------------------------ACCGAGG-PPYNFNA---TLGQCGT----------AAASTYSDRTQFVIWDGIHYT
B8    NKAVKYYNTLLNKTLSQTRTEL--KNATVIYLDTHKILLDLFQH-PKSY--------------------------GM-KHGIKACCGYGG-RPYNFN----QKLFCGNTKVIGNF--STTAKACHDPHNYVSWDGIHAT
B24   NSGVTYYNELLNNSLAEVRKKL--QDASILYVDKHTVTLELFQH-PTAH--------------------------GL-KYGTRACCGYGG-GTYNFN----QDVYCGNSKVVNGK--TATAGACGDPQNYVSWDGIHAT
B10   NQAAKLFNLQLHAMSNKFQAQY--PDANVTYVDIFSIKSNLIAN-YSRFGKHFTKPLIDLNHLENVGYNKILNVLGF-EKPLMACCGVGG-APLNYD----SRITCGQTKVLDGI--SVTAKACNDSSEYINWDGIHYT
B16   NRAANVFNSQLLDLCTKFQGQF--PDANVTYVDIFSIKMKLIAD-FSQY--------------------------GF-KHSLAACCGYGG-PPLNFD----NRIACGQTKVLNGS--KVTGSPCNDTAEYVNWDGNHYT
B29   NRAAKLFNSQLHSLCAKLGSQL--PQVNVTYVDIFAIKLNLIAN-FSQL--------------------------GF-KESIAACCGYGG-PPLNFD----NRIACGQTKSLNGS--LVTAKPCDNTTEYVNWDGNHYT
B17   NEVARYFNYKLKEAVFKLRKDF--PSAAITYVDVYSVKYSLFSD-PKKY--------------------------GF-ELPLIACCGYGG--KYNYS----DAAGCGETITVNNT--KMVVGSCDNPSVRVNWDGAHYT
B26   NEVAQYFNYKLKEAVSQLRKDF--PLAAITYVDVYSVKYSLFSQ-PKKY--------------------------GF-ELPLVACCGYGG--EYNYG----NDAGCGSTITVNGS--QIFVGSCERPSLRVNWDGIHYT
B27   NEVAQYFNYKLKEAVAQLRKDF--PLAAITYVDVYSVKYSLFSQ-PKKY--------------------------GF-ELPLVVCCGYGG--EYNYS----NDAGCGSTITVNGS--QIFVGSCERPSLRVNWDGVHYT
B28   NEVAQEFNRQLKDMVSQLRSKL--GDALLTYVDIYSAKYSLISE-AKIH--------------------------GF-VDPFGQCCGQNG--------------KFRECGKKAVVNGT--EVDGASCTNPSEYVSWDGVHYT
B30   NNAAKKFNKQLKALCKKLRAAM--KDVTIVYVDIFAIKYDLIAN-AKLY--------------------------GF-ENPLMVCCGHGG-PPYNFD----NLIQCGG----------VGFSVCEEGSKYVSWDGIHYT
B33   NEAVETTNSLIRSGLKDLRSKH--PDATIIYADLYTILKDLIVN-GTSY--------------------------GF-KETFKACCGAGG-GAYNLN----PNVSCGLSALVNGQ--LIQGTSCSDPGSYVNWDGVHVT
B6    NKFGEYHSEQLQEELNRLRKLN--PHVNIIYADYYNASLRLGRE-P-----------------------------RFINRHLSACCGVGG--PYNFN----LSRSCGS----------VGVEACSDPSKYVAWDGLHMT
B7    NEFGEYHNEQLKTELKRLQELY--DHVNIIYADYYNSLFRLYQE-PVKY--------------------------GFKNRPLAACCGVGG--QYNFT----IGKECGH----------RGVSCCQNPSEYVNWDGYHLT
B15   NEFTEYHNRLLQEELEKLRNLY--PDVSIIYADYYGAALNIFLA-PLQF--------------------------GF-TVPLNSCCGSDA--PYNCS----PSILCGH----------PGSVVCSDPSKYTSWDGLHFT
B11   NEIAVYHNTLLQDAIKNVQKNH--PDVRVIYADFFTPVIRIVQS-PGTF--------------------------GFTSDILRCCCGGGG--KYNFN----MSAGCGM----------PGATVCEDPSTHLFWDG-HMT
B13   NEIAVYHNTLLQDAIKNVQKNH--PDVRVIYADFFTPVIRIVQS-PGTF--------------------------GFTSDILRCCCGGGG--KYNFN----MSAGCGM----------PGATVCEDPSTHLFWDG-HMT
B12   NELAILHNSLLQQSLRNLQARH--PDASIIYADFFSPIMEMVQS-PGKF--------------------------GFEDDVLTICCGGPG-------------TALCGN----------QGAITCEDPSARLFWDMVHMT
B21   NNLSSYHNELLKQAVAGLQSKHA-AGVRLMYADLYAQVADMVRS-PETF--------------------------GL-KYGLKVCCGAGGQGSYNYN----NNARCGM----------SGSSACGDPEKYLVWDGIHLT
B25   NDLSTNHNNQLQTQISSLQAKY--KSARIMYADFYSAVYDMVKN-PGSY--------------------------GF-STVFQTCCGAGG-GKYNYQ----NSARCGM----------SGASACSNPAAHLSWDGIHLT
B22   NRLAFHHNRELKQQLDELQKKY--PKTKIMYGDYFKAAMQFVVY-PGKF--------------------------GF-STALQACCGAGGQGNYNFN----LKKKCGE----------QGASVCSNPSSYVSWDGIHMT
B23   NTFSWVHNAMLKRALEKLREKH--PGVRIIYGDYFTPIIQFILQ-PKKF--------------------------GFYKQPPRACCGAPGRGPYNFN----LTAKCGE----------PGASACADPTTHWSWDGIHLT
B14   NKLAEVHNRELTRMLRQLRRAF--PAAAVHYADFYRPVTAIIAS-PAKY--------------------------GFGDTPLAACCGGGG-NPYNFD----FAAFCTL----------RASTLCADPSKYVSWDGIHYT
B9    NNQSYTHNLALQSKLKQLRIKY--PSATIVYADYWNAYRAVIKH-PSKY--------------------------GI-TEKFKACCGIGE--PYNFQ----VFQTCGT----------DAATVCKDPNQYINWDGVHLT
B18   NDFIKYHNDLLREAISRLRKEH--PDVHILIGDYYTAMQSVLDN-HQKL--------------------------GF-ESVLVACCGTGG--KYNFD----HRKKCGT----------QGVQSCSDPRKYISWDGLHMT
B19   NDLYNYHNDRLKEAIEELKKEY--PHVDIVYGDLYKAMQWIMDN-SRQL--------------------------GF-KSVTKACCGPKS--EYNFIDN--FHKMCGA----------PNIPVCQKPKQYVYWDSGHWT
B20   NDFCNYHNVLLQEGLKELREQH--PDVQIVYGDLYNAMQSILDN-SQSL--------------------------GF-KSLTEACCDVDV--EIKKKAVLYKDKLCGA----------HGTIVCPKPEEYVFWDNGHCT
B31   NSVIEIANGKLKAAVDEYRRKW--PDTIFLHYDSYGAALEVIQTGPAKY--------------------------GIDADGFRACCGGGG--PYNFN----PFVLCGSG---------KIANVCPDPEHKLFWDFIHPT
B5    TQVFETFNKQLLDMVIDINYQN--DDINIYHFDWFAATDHVIKN-MHHY--------------------------KF-KSYKSACCGIPG-NDYHCE----GLALCGCGQ--------TNGTTCKNPGEHVTWDGTHYT
B32   NKISEAHNTLLGLKVEELRKKY--PDAKLYYGDVYAVYTDILKE-PAKY--------------------------NV-TAPLKACCGVGG--DYNFN----KDVWCGQSGTVEGKFVNLTSTYCADPVSTLSWDGIHTS
```

```
B1    EHLYHIVANAFLTGQYVD--------LV---ANQKLDQ-----------------------
B4    EHFYEIVANAFLSGEFLD--------PP---IFPKLSQTI---------------------
B2    QHYYQTVAEFFLSGIFLD--------P----------------------------------
B3    EAFYEHLSKALLTGKYLD--------PP---LDFS--------------------------
B35   EAFYRQIAKFFLNGQFVT--------PA---LNLAAECGLKFDDFYKKS-------------
B34   EALSKLVAKTILQCKFVD--------PVG--FNFSSLCDLDFNKFDDETAR-----------
B8    EAANHHISMAILDGSISY--------PP---FILNNLCSP----------------------
B24   EAANYKIAYAVISGSYSY--------PP---FDLSKLCSP----------------------
B10   EAANEFVSSQILTGKYSD--------PP---FSDQMPFFLTLKF------------------
B16   EAANRYVSEQILAGNYSNQLPLSINTPA---LTTSMFVDSKLLY------------------
B29   EAANLYVSSQILTGKFSD--------PP---LTEFTISPSVFHTGFRKRFRF---------F
B17   EAANKFVFDRISTGAFSD--------PP---IPLNMACHRNVSFSVLSIS-----------F
B26   EAANKFVFDQISSGAFSD--------PP---LPLRMACHRNTSY------------------
B27   EAANKFVFDQISSGAFSD--------PP---LPLKMACHRNASY------------------
```

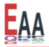


```
B28     DAANQWVAGHILNGSLSD--------PP---LPISEACHKPLHLELK---------------
B30     QLANQFVASKILSTNFST--------PP---LHFDFFCD-----------------------
B33     DAAASFIARAVLQGKHTE--------PV---YKLTELCRLSFEQFSPSPP------------
B6      EAAHKSMADGLVKGPYAI--------PP---FDWSCLSSMIKKKEVVGNTIFFDE-------
B7      EATHQKMAQVILNGTYAS--------PA---FDWSCSGSESVDKEYSFSS------------
B15     EATYKIIIQG-QSAVYDH--------PA---MFSSIS------------------EIGPPVF
B11     EAAYHFIADGWLNSINES--------------------------------------------
B13     EAAYHFIADGWLNSINES--------------------------------------------
B12     EVAYRYIAEDWLRIRVTW--------EQ---NNLSVF--RVHKKIYTESTKF------SVLI
B21     DAAYRSIADAWLKGTYCS--------PG---ILR----------------------------
B25     EAAYKQITDGWLNGPYCR--------PA---ILHS---------------------------
B22     EAAYKKVADGWLNGPYAE--------PP---ILKS---------------------------
B23     EAAYRQIARGWLHGPFGD--------QP---IVQSS--------------------------
B14     EAVNKFVARSMLRRALIP---M----PK---PNPSLSMPLSSSREHTGQETS-----RELAT
B9      EAMYKVMADMFLDGTFTR--------PR---FSDLLIKKLNY-------------------L
B18     QESHKHIAKWYIQDIFSK-------------FQS----------------------------
B19     QNANKHLAKWLIRDIF----------PK---FHCKKV-------------------------
B20     QKANEQLADWIIQDIF----------PK---FQCNA--------------------------
B31     EAFFRVMATFALSGQYVD--------GPPEVANLKAACNLDFSSFAQSIPNSPSCPRDTAVT
B5      QHFYEVSSQFVLHGNFIS--------PR---LNLLPGCGIPMPPSEN---------------
B32     NTVNKALATAFLTGKHIY--------PE---GGLKCNADFKFWEART---------------
```

GROUP C

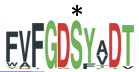


```
C1      MNIT-----------------------KLTPWFLFSCLILLS----------------DYIKVNS--SISPSSEQTQEDGFFGFKPTKLFVFGDSYADTGNTP----FLIVPSWRFPNGITFP--GIPTGRFSDGRVST
C3      MEKR----------------------GSLFLGFLFFSLVTISTGQV------------KEIKNSETLAMCPVPENSSPKG----HSPKLFVFGDSYVDTGNL-------------QPF------------RSSGGCVLT
C5      MDHQ----------------------PLFIYLFLLLS-VTISAAGISLTIIYPFVYMKKCMIVLSLIVPFPITGAYVDPD----RSIKLFVFGGSYVDTGNR-----DSTARSWKEPYGITDP--GRPAGHFSDGQVFT
C6      MNLT-----PNWKS------------------------IAVNVQAT----------------------------------------SLLFAFGDSYADVGNT----LKGISPAWRYPYGITWPL-HDPAGRFSDGKIST
C2      MAAKLSLLLPV-----------------TCFIL-----FVLHAVHVE--------------------ARPDPAAFGDSDN----GFYTLFVFGDSFADTGNLPKRRLSEQSREWYYPYGRDRGN-NRPTGRFSNAMVQS
C4      MD----LIITVSKVIVCKGADHGCKHYKTCFLFIYSTYTLLSVNHV----------------------RQPMRAMARALH----WYNGLFVFGDSFADTGNSPKSDLSEVTRQWYKPYGSSHGFLRDPTGRFSNSLVQS
```

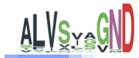


```
C1      DYLAKYIGVR-TPITYKWGKYGRPRLAVKRGMNFAYGGAGAFETMFKLVPTASVQIDSFEQLLMRN-VYSPADLNSSVAFFSIIGNDYL-TYDRRNGSEEGRS-------ALTRKVVKQILLDVKRIKDLGVRKVLVAL
C3      DYIASFLGIN-SPVQYEKRNLAENKAELKNGMNFAYGGSGVLKEAWN-NHSMTIQINNFKQQIKEK-VFTKYDLENSAALVSHAGNDYTYLYLNQSGPIKDVH-------ALAGRVVDQLVKNVKEIHELGVKKIAILG
C5      EYIASRMGIK-SPTPYRFR----NQRPIKHGMNFAYGGTGVFDTWVA-APNMTEQIYLFEQLLQEK-IYTRYDLKFSTALVSASGMDYR-IYLARRGSLRVSA---------LLHGLPLLGLDLQRIHDLGVPQVAVMG
C6      DWIADLLGLPLYPPPYLYT----AGENISYGVNFAVGGSGVFKVLSN--VSLDVQVDNFELFLRTD-PYSKAALENSVTYVSVGGNDYL-AF---RGTTEAVSLKPNERLLYIERVIRGIQANLQRLYDLGLRHVMVAN
C2      DLIARMLGRHEAPPTYRRV----DNYVHPHGMNFAAGGSGVFKLPSG-APTLDKQVDHFRDLVQDG-TITRRNLRNSIALVAVSGNDYA-RLA--NVNDTSKM------IKFVDEVTSEIAKQVHRLKNNGARKILVNN
C4      DFIAKILGRSEAPLTYRRTK---AGYPDKFGVNFAVGGAGVFEVPRE-APTLARQIDDFEKMLDDDRTIGKWQLRQSVALVAISGNDYA-RVAANTSSDGADI------TGFVGNVTDEIARGVDRLRKLGVTKVLVNT
```

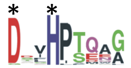

```
C1     SPPQKCLPKLVTP----KGCDTNDTSTYLHNSLLRKGLIKLNDKEINNNDKSFMTLDLYNAFVTIFKN-KGVSG---VSTFPDPFKACC-ATKRGTFCGDRSLSGKKLYTLCDDPKSFFFWDNVHISDQGWRSVFSLLL
C3     SPPRGCWPQLN-PRPRTNCNATWNEESRFHNQLLTEALKNVEES----EKNAFVFLDLYKAMDLALQKNKE------NSDYENPLEPCCDGVSDEYWCGMKDQKGAEMFTVCRQPESSFFWDKVHPSQNGVHAIFLDLI
C5     MQPLGCLPEFTRGYSYEKCNSTGNFAALYHNLLLVKKLK---------PESKFVVLDMYNAFMSAMKKFENKTE---TSELKNPLRPCCTGITSEYRCGDVENKEKK-YNVCRVPKLAFFWDSFHPTQAGWDAIVSELG
C6     IPQPDCLPLFTEKNNWTNCTGETGPLINIHNSFLLVAVENINAR---NPGARFIILDHYSAFSRLLSEADE-------QGFTDGLKPCCTGTTNTTKCGDVDASGKWLYTVCKHRGRALFWDSEHPTMWAWHYIIDLYT
C2     LHPVGCTPWVTRPGNYSGCSSTGNMGAYLHGSNLQQKLSHL---------DYVHHVDLNTAFSNIVNP-DQGSKHKVSSQFEHKMQPCCESLDPNGFCGQKGHDGKDLFSVCNDPEKYFYWDDVHPTEAGWKAVMQQLE
C4     LHPLACTPWQSRPSNYTKCVGRGNMAADLHNDHLRDKLRNATA------SDSVYLLDLNRAFTTIISPSDTDTVPQVAKQFKEKLRPCCESCDPNGYCGQVDKDGGAQYSVCSNPEKHFFWDDVHPTQAGWEAVMEQLE

C1     PDSQF-----------------------------------------
C3     PSLERLLLIC------------------------------------
C5     SSIISLF---------------------------------------
C6     KQPNYILLAGVPTLRDWLQNNNAAPEPTAALMSQPGESILPCQRES
C2     GPIKKFLRIN------------------------------------
C4     RDIKDFLHIS-----------------------------------Y
```

GROUP D

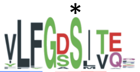

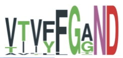

```
D1     MLASVTPGAAAAPTS-------------AGSFVAAARPRIILFGDSLTERGFDQPGGWAAYMAANYTRRADVVNRGMSGYNTRWAVQVLPYVFGQPTASAGSGGSGGGA-GALAGQVLFATLFFGANDAARKEGPEHSA
D5     -MASVHGGKSAAVGA-------------GLSFLSASRPRIILFGDSLTERSFDNPEGWGSSLASFYVRRADVVNRGMSGYNTRWAMETLPYVFG-PTLTPGV----PSP-TAASERVMFATVFFGANDAARLEGPSHSA
D2     ----------------------------------MLRSQFILFGDSLTQKSFDE-GGWGGRLANEYQRKVDIFSRGYSGYNTRWAKYIAPLIFP----------------GSQKVPPQLVTVFFGANDAALPD--RLSA
D6     --------------------------------MGPARPRIVLFGDSITQQSFGP-GGWGAALAHHYCRKADIVLRGYSGYNSSWALFLLHKIFP--------------S-SLEEDAPLAVTIFFGANDAALPD--RGSS
D8     ---MTTPGSCPSSTSAAHSPVCLAPSAAAGESQEMVRPRMVLFGDSITEQSFRP-GGWGAALADTYSRKADVVVRGYGGYNTRWALFLLHQIFP----------------LVGIVPPLATTVFFGANDAALLG--RTGE
D10    -----------------------------------MRPSIVLFGDSITEEAFGE-GGWGASLANHYSRSADVVLRGYSGYNTRWAARVAGRAV-----------------ASIAGPVSAVTVFFGANDAALPD--RACA
D7     -------------------------------MVGPVRPQIVLFGSSIVQYSFTD-RGWGATLADLYSRTADIILRGYAGWNSRFALKVLHQVFP----------------KDAVIQPSLVIVYFGGNDSTHPH--PSGH
D9     -------------------------------MVGPERPQFVLFGSSIVQFSYSN-EGWGALLAHIYARKADIILRGYSGWNSRRAVQILDQVFP----------------KDAAKQPSLVIVYFGGNDSTHPH--ATGL
D11    -------------------------------MVGPPRPQFVLFGSSIVQFSYSN-QGWGAILADLYARKADVVLRGYSGWNSRRALQVLDQVFP----------------KDAAVQPSLVIVYFGGNDSMHPH--PSGL
D12    -------------------------------MAPYARPKFVLFGASMTEYSFCH-GGWGAALANLYCRKADIVLRGYRGWNTRRALEVLDNFFP----------------KDAENQPELVVVFFGANDGAFPM--PSGR
D4     ------------------------------------RESVVLFGDSLTERSFED-GGFGARVQHEFRRFADVRCRGYSGYNTEHALCLLDEVFP-LNEDVDD----DGAFATYKRAPVLVTILFGSNDACAKNS-SAGD
D3     ------------------------------MVASGRRRAFLLFGDSLTQRAFEC-GGWGARLAHLLSRKADIICRGFGAYNTRWCRHVVRHI------------------GSYRDYFSVVTVLLGTNDAALPD---VEP
```

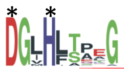

```
D1     RQHVPVDEYGRNLREMVSYMRATGIS-RILLLTPPPVWAPGRRKHMLWRVGE-------ASKDWPLDRTQEATQPYARAAAEAAQELGVPCLDLNTLLQ-QEEDWGERLLCDGLHLTPTGQEKLWSLVREAVWREW-PE
D5     RQHVPLDEYRSNLKEMVRYIKATGVE-KVVIITPPPVSDAGRKAAQIQKMGE-------QARDWPLDRNFATSAQYSKAAADVAKELGVPCLDLFALLQ-EEDRWQERCLCDGLHLTPLGQEKLGNQLRSLLRQEW-PD
D2     RQHVPVEEYRSNLQSLIERIQKIGTK-NILLITPPPLDEAARIRHNQQQNNEV-----SEATSIA-ERTNSITGQYAAAAKQLAGDLGLPVLDLWTAIQ-KHESWQSRYLEDGLHFTPAGNKAVFDLLLETLRGAF-PH
D6     HQHVPLPTYKTNLKRIISHLKAVSKRTHIVLITPPPIDEKARREFAIHTYGR-------DAHELP-ERTNAVAQEYASACKAVAAEENVAVIDLWTLFQ-KNHDWRSIYLSDGLHLTAAGNGVVFDQVVQAFKLA----
D8     RQHVPVAEYKENLKKIVNHLKDCSKSMLIVLITPPPIDEDGRERFARSLYGE-------EARKLP-ERTNEMAGVYASQCIELAREMNIHCIDIWSKMQ-ETAGWQKLYLSDGLHLTPEGNAVVHKEVVQTLRSV----
D10    LQHVPLAEYKDNLRAICALLKKRWPSVVVILITPPPVDEDGRLRYP---YAH-------DFSGLP-ERTNAAAGLYAKACLEVARQCGLRAIDVWSRMQ-RFHGWEKSFLRDGLHLTPRGNRVLFEEVVFALKDA----
D7     GPHVPLSEFIENMRKIGEHLLSLSDKTRVIFLTPPPMNEKQIEIVF-GDAIK--------------GRSNELCRPYAEELLNLCREINVKGIDIWTAIQ-QQDDWLNSCFTDGIHFTAKASEIVVKEILKVLRGADWKP
D9     GPHVPLSEYIENVRKIAIHLKSLSEKTRVIFLTAPPVSDEQIRAHL-GDLLDM-------------VRTNESCRIYSEACLEVCREMNLKAIDLWTATQ-QIDNWETVCLTDGVHFAPEGSKIVVKEILKVIKEANWEP
D11    GPHVPLPEYVENMKKIAVHLKSLSEQTRVIFLSAPPINEAQIRETLSGRLGTL-------------GRTNECCGIYSEACLELCKEVGVKAIDLWTAIQ-QQDDWLNVCFTDGIHLSSEGSKVVVKEILKVLEEAEWEP
D12    GQHVPLPEFEDNLCRISAHLQGLSDKTRVILTTAPPIYEPARLEAGRAKHGE-------KGAKYL-DRTNERACQYAACRRAAHRMGAGIIDLWTSIQ-RQPDWQTSCLTDGMHLSAQGSGVMLEELLKVLKDSPWQP
```

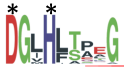

```
D4     VQHVPLPRYEQNLKTIVERVRRMQPSPRILFITPPPVDDEAWLRDCATRAAQPGLGFGSLLNDTAPNRTNAGVKPYAEAMKRVARFYDIPVVDLHAALEFSNGEVDETQFCDGLHFSEAGQRQVASLVIDALRQ-----
D3     VQAVPLDEYVENLDDILKYLRNRSE--FVILFSPPSVGELGRLRAQHHKYVA-------DAHDWL-DRNNLHSAKYASVAKVVAENRALVCVDMFRLTS-VQLFLGENMLIDGIHFTATGHLFLLKSLLHELRAEA--H
```

```
D1     -TRPEALKTQFPAWDAIDFADIASSFLPQ---------GEQQGQDGAAKEK----------------------------------AGGKAAAAAAA----------GVANGAKA
D5     -IRPIDLPTQFPAWDAINFEDVKSSFI-------------------------------------------------------------DTEAAS--------------------
D2     -LRAENLPDDFPSHKDIDEDDPEKAFI-------------------------------------------------------------SYA-----------------------
D6     GIAEAELSLDFPLHSSIDKEHPEIAFST--------------------------------------------------------------------------------------
D8     GLKAEEMPYDFPHHSRIDGSCPEKAFQ---------------------------------------------------------------------------------------
D10    NLSLEALPADLPLFGDMDPDNPAKSFE-------------------------------------------------------------DHE-----------------------
D7     SLYWKSLPVEFP----FDFDAPN---SISLHDLELTRNNHFESPHLVSLCEQELTRNEQLEPPHPVSLCDHELTRNEQLEPPHPVSLCDHELTQNEQLEPPQPT-------ARL
D9     SLHWKAMPTEFSEDSPYDPISPEGK-TVNVSELDLLGSFQWE------------------------------------------------------------------------
D11    SLHWKSLPSEFDDDSPYNPGSPVGKTTLKVADINLHREIEWN------------------------------------------------------------------------
D12    SLHYDAMPEDLLGPHIYNFLHPCEE-DVEEARVSS-------------------------------------------------------------------------------
D4     ------------------------------------------------------------------------------------------------------------------
D3     ILSAENMRPDWPYGPWMQNSAGSWREILTEHELEI---SHISGDKLSALAD----------------------------------AFGIVSVFISAFLFGLSVSCRGL---ATL
```

GROUP E

```
E1     M------------YRANIVVAVCAFALLLS-------------------------------------------------------------------------------------------------------------
E2     MHLKLQTCGKMAILRARSLTAV--LFLSFT-------------------------------------------------------------------------------------------------------------
E3     MSPARRQC-------AAPLAAA--LLLLLAAGCALASASRTDDGFSGNYNKKGGKNPGSRRASPPPARRRYPPPVGSGGLTTSRPSPAAAPGGGDTRERIDLVVFGDSLSDTGNTFRAAGVPQADLYYQGRYSNGPVWI
```

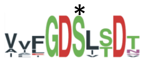

```
E1     ---AAH-----------------------------------------------------------------------AAD----------------KTVYQYVIFGDSVTDTGNVFRDGGVPDPLIYYKGRFTNGPNWV
E2     ---AAHLCN--------------------------------------------AIRIPPS---------------PPASG----------------FTRYNMVFLGDSLTDQGNAFAMLGAPNPSIYYKGRCTNGPNWV
E3     DYLAAEVANNNSATTVLNLIADNLLMFGFWPLQGRRGNTGRRQLTGSRHPPLRAIIPPPGKNVPRVRRAPPPHRHPPASGGGLKRPPPAAASGGDTRERIDLVVFGDSLSDTGNTFRAVGVPQADLYYQGRYSNGPIWV
```

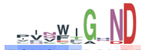

```
E1     DYLNQSLS--AKHKVQIFNYATGGGVACSFNLNNTRYPYARDAVNQTGNFLADLARGKVPTTKY-----TRRISVSWFAANDITVALSR-ALTSGV-DAATAGRQIVSSLATCLSQHVGALAA-SG----------VRE
E2     DYLTKTVKQYPQTRIRVQNYAFPGATACPSPLTRAAAPFITDMSDQIAALTADVASKKVRLSG------SKTLVFQYLGTNDFLFFFENLQATNGT-TTQAEIAQLVRDTITCRVKGAAAIAAIQG----------VTD
E3     DYLAAAVA--NTTQLTVLNYAHGGAAACPENSVATQIRFIRDLPAQTSAFIANISSAPQQQSQQGGGGGRRLLPINFIGNNDVRNVLSA-GISGGVYPTEQSIGALALAITSCRLTWAKQLLA-AGEAVWGQQRGPERV
```

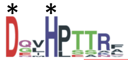

```
E1     VVLLPQSPVQVAPLVPDFLRPSVAQLVDAVDAALVQAMAAVNAQLDAAPAGSPAAGAHVYLLGDSKWIGRIAPAVRPAFKYMGDRSCFTNPDSSMRITPGLADRICTDPNDYAFYDEIHPTTNFHKAYALDALLPRLQL
E2     IVILPIAPLHTSPAAPPEYRPVFLAIEAFLGNETRAAMEQLNKTLAAAPAGTPGAGVKIWVLGNTDWVSNGAYQVKPPFKYIDTAPCFWNPRPVIVITPGIQ--VCSDPEDYFFYDQVHPTTRFHEWFANKGVLPRLQD
E3     LVLLPLARLDLTPSVPKELKPTLKAVTDAINNFLTAAVAALNAELAAAPVDSGSRGARLLVL--PDFTGVVAATVTPPFTNFDEA-CFFHPAYVLDVVPGIV--PCTDPDTHVFYDQLHPTTRLQKAFALSGVLPALQQ

E1     FRLAPRDV
E2     LGVLP---
E3     AGLLPAAA
```

GROUP F

```
F1     MLIVI----------------LAAALIQPSP------------------AFVLSGDAQDRAFEDMSMLARAGKLLTVAVSDAASAIGATDRRSGASEKRPLIISDKKVAKKAHGHGGIPSWFKGAADVNAHPATVLEGN
F3     M------------------------------------------------------------------------------------------------------------------------------------------
F2     MNPPIDPPGGEIEEAPLPHSSSSGADVESGPGAPPKVPPPAKAWTMIIGAFVVL----------MFMLASVRTTTAMRQRVRDSTLEQIPNADGSAEKVPVFGPE------------IPRIRSKGTLCIKHP-------
```

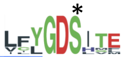

```
F1     RRMLRRQKQHHHSVEDE---------------WLKVHRRYVEEMEAAESAAGRGWDVLFYGDSIMEEWRWDPPPDVPPQLQHQWGSFLGSAWGPFSDVKKVWAEFFATKPYRSLPLGIAGDK----VEQLLWRLQNGEL
F3     ----------------------------------------------------QGYDVVIYGDSISENWR-GTSGGLP---------YIRKDNTSS----------------RPDPNFVAADMRAAFLETLGQRLLNGQT
F2     ---------HHLPIAAEVPPPEPEPQEVNAVAW----RKFADAL--ADEAAIDGSKIVLLGDSITEAWR-GTQLDIRQKQYDRAGDIIRKTLGDM----------------DPRILAIAGDQ----TKHLMWRLEHGGF
```

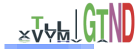

```
F1     PATKH-PKVVTVMIGTNDLVGN------------C--------TLTSAAET-AAGIVEINSLLHAELPTTHILNLAVLPKGE------------VWPNRCSEAILAVNSELEEY-------SRANPS---------FAH
F3     PKVD--PKVAVVLIGTNDLYAATQ----------CAAQDE--DQLKSAVKTIVTGFQQLLAAMRQGMPSTHIVVQALYPRGE-----------------------DMEDNMFEL-------TRSDP----------FMH
F2     PSEKSPPDYVTLMIGTNDVGAAVRTKNGGWQNGMCVSDDNVGDSLDAVDPT-VAGVKAIVEKIRKLAPKARLVVLGLTPRGEKHGKGWSERRSYLQPSLFTAAIDEINRRVEEYVASVEEVNRRRPTKGLFGKRRGVTV
```

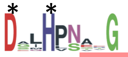

```
F1     YADFGKGFLSDEPVGGRYEVKEALMPDSLHPSAVGMRIIASQLEPLIAS-------LVR--------QPIG-----DAAADADWATSR
F3     YISCGEEFVTPTSD----AIVKEYLPDGLHPNAEGLRLIAECLTPLLQK-------LTSSNATTNAIQPAQNRVDLEASASELW----
F2     FQPCAEGFLKNDGG----EIRKDLMRDALHPSGRGLDVLMRCITDGMDKANVEHDRLVA--------AGEE-----DAGEDAEQP---
```
